# Development of a high-density linkage map and mapping of the three-pistil gene (*Pis1*) in wheat using GBS markers

**DOI:** 10.1186/s12864-017-3960-7

**Published:** 2017-07-31

**Authors:** Zaijun Yang, Zhenyong Chen, Zhengsong Peng, Yan Yu, Mingli Liao, Shuhong Wei

**Affiliations:** Key Laboratory of Southwest China Wildlife Resources Conservation (Ministry of Education), College of Life Science, China West Normal University, Nanchong, Sichuan 637009 China

**Keywords:** Wheat, Genotyping-by-sequencing (GBS), Three-pistil mutation, Kompetitive allele-specific PCR (KASP)

## Abstract

**Background:**

The wheat mutant line three-pistil (TP) exhibits three pistils per floret. As TP normally has two or three seeds in each of the florets on the same spike, there is the possibility of increasing the number of grains per spike. Therefore, TP is a highly valuable mutant for breeding and for the study of floral development in wheat. To map the three-pistil gene (*Pis1*), genotyping-by-sequencing single-nucleotide polymorphism (GBS-SNP) data from an F_2_ mapping population (CM28 × CM28TP) was used to construct a genetic map that is of significant value.

**Results:**

In the present study, a high-density genetic map of wheat containing 2917 GBS-SNP markers was constructed. Twenty-one linkage groups were resolved, with a total length of 2371.40 cM. The individual chromosomes range from 2.64 cM to 454.55 cM with an average marker density of 0.81 cM. The *Pis1* gene was mapped using this high-resolution map, and two flanking SNP markers tightly linked to the gene, *M70* and *M71*, were identified. The *Pis1* is 3.00 cM from *M70* and 1.10 cM from *M71*. In bread wheat genome, *M70* and *M71* were found to delimit a physical distance of 3.40 Mb, which encompasses 127 protein-coding genes. To validate the GBS-generated genotypic data and to eliminate missing marker data in the *Pis1* region, five Kompetitive Allele-Specific PCR (KASP) assays were designed from corresponding GBS sequences, which harbor SNPs that surround *Pis1*. Three KASP-SNP markers, *KM70*, *KM71*, and *KM75*, were remapped to the *Pis1* gene region.

**Conclusions:**

This work not only lays the foundation for the map-based cloning of *Pis1* but can also serve as a valuable tool for studying marker-trait association of important traits and marker-assisted breeding in wheat.

**Electronic supplementary material:**

The online version of this article (doi:10.1186/s12864-017-3960-7) contains supplementary material, which is available to authorized users.

## Background

Wheat (*Triticum aestivum* L.), which is considered to be the second-most consumed crop, is cultivated widely around the world. However, in recent years, levels of wheat production have not satisfied the global demand, triggering price instability and hunger riots. Accordingly, a major goal of wheat production is to increase yield. One effective method of improving wheat yield is to increase the number of grains per spike [1, 2], which can be achieved through a wide-range of genetic variations in the morphological framework of wheat, including superfluous spikelets and multiple spikelets [3] as well as multiple rows of spikes [4]. Described by Peng, three-pistil (TP) mutant wheat has normal spike morphology but exhibits three pistils per floret [5]. As a result, three seeds can be cultivated per floret, increasing the number of seeds per spike. Previous studies have shown that the three-pistil trait is controlled by the *Pis1* gene, a dominant gene located on chromosome arm 2DL [5, 6] between simple sequence repeat (SSR) markers *Xgwm539* and *Xgwm349* [7]. However, at 17.6 cM and 19.5 cM from *Xgwm539* and *Xgwm349,* respectively, *Pis1* is too distant to meet the requirements for fine-mapping [7]. In recent years, our group has explored genes contributing to the three-pistil trait in wheat. Although a large number of relevant genes have been cloned [8–11], the *Pis1* gene has not. Map-based cloning is the best approach for the cloning of *Pis1*, the first step of which is to find markers closely linked to *Pis1* gene. Nonetheless, common wheat (2n = 6× = 42) has a large genome (16 Gb), with highly repetitive sequences; thus, fine-mapping of functional genes is challenging.

Recently, scientists have been able to determine allelic variations corresponding to complicated traits with the help of advances in next-generation sequencing (NGS) technology [12]. Several methods that combine marker discovery and genotyping have been developed, such as reduced-representation sequencing, restriction-site-associated DNA sequencing (RAD-seq), and low-coverage genotyping, which includes multiplexed shotgun sequencing (MSG) and genotyping-by-sequencing (GBS). To some extent, these approaches are similar in technique. By generating the same amount of data per sample with a 96-plex library, GBS is beneficial for improving outcomes and reducing the cost per sample, making this genotyping platform quite appealing [13]. The two- or three-enzyme GBS approach can be applied to genotype species with large and complex genomes, such as barley, or species with polyploid genomes, such as common wheat [13]. Developing high-density GBS markers in hexaploid wheat may promote progress in the identification of the physical locations of genes of interest.

According to latest research, we constructed a high-density linkage map of wheat based on an F_2_ population of 200 individuals derived from cross of Chuanmai 28 (CM28) with its near-isogenic line CM28TP using SNP markers obtained via GBS technology. The *Pis1* gene was then mapped using this high-density linkage map. This work not only lays the foundation for map-based cloning of the *Pis1* gene but can also serve as a valuable tool for the identification and genetic dissection of many other complex and important wheat traits in the future.

## Results

### Sequencing the parental lines and the F_2_ population

The parental lines CM28TP and CM28 were sequenced using GBS at efficient sequencing depths of approximately 23.3-fold and 19.0-fold, respectively. With regard to CM28TP and CM28, 29,464,252 and 21,332,415 reads, respectively, were mapped to the sequence of the bread wheat genome (IWGSC1+popseq.31.pep, ftp://ftp.ensemblgenomes.org/pub/plants/release-31/fasta/triticum_aestivum/pep/). A total of 727,097 and 679,440 SNPs were identified in CM28TP and CM28, respectively. For the F_2_ population, the efficiency of enzyme digestion was quite high, at 94%. A total of 1,862,290,768 reads were produced, with an average of 9,311,454 reads per individual; this is equivalent to approximately 0.23-fold coverage of the bread wheat genome. The average GC content of the sequences is 40.5%, with a Q20 score of 93.9%. Given that the two parents are homozygous inbred lines with *aa* and *bb* genotypes, only the genotype *aa* × *bb*, consisting of 21,584 markers, was used for further analysis. Among the 21,584 markers, the low-coverage sequences of the F_2_ population (coverage less than 75%) were filtered, leaving 16,475 markers. Markers with significant distortion (*p* < 0.001) were filtered, and 3655 markers were retained in total with the purpose of determining bin markers.

### Genetic linkage map with bin markers

**Fig. 1:**
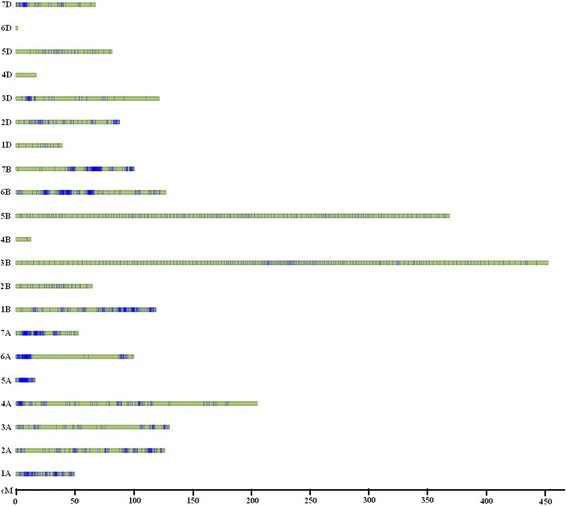
Distribution of the GBS markers on 21 linkage groups. The genetic distances (cM) are shown on the *x*-axis

A total of 2917 SNPs (1987 bin markers) were mapped to 21 linkage maps; 738 SNPs were unanchored to any chromosome (Fig. 1, Additional file 1: Figure S1). These genetic maps spanned a total length of 2371.40 cM, with individual chromosomes ranging from 2.64 cM (6D) to 454.55 cM (3B) (Table 1). The average distance is 0.81. The number of bin markers in the different chromosomes range from 2 (4D and 6D) to 192 (3B) and are not evenly distributed on each chromosome. A total of 846 bin markers were mapped to the A genome, covering a distance of 690.27 cM. For the B genome, 905 bin markers were mapped, covering a distance of 1255.69 cM. However, only 236 bin markers were mapped to the D genome, covering a distance of 425.44 cM (Table 1). A total of 87 gaps from 5 cM to 45 cM were observed, and the largest gap, 44.39 cM, was found on chromosome 4D (Table 1).

**Table 1:** Marker information for the high-density genetic map

Chr.	No. SNP	No. Bin marker	Genetic distance (cM)	Average distance (cM)	Max. gap (cM)	<5 cM gap
1A	119	119	50.43	0.42	2.51	119
2A	132	132	128.02	2.37	10.44	128
3A	75	75	131.82	4.59	10.13	68
4A	121	121	206.66	2.41	5.70	112
5A	242	135	17.60	0.71	12.44	134
6A	125	125	101.49	0.59	18.61	122
7A	239	139	54.25	2.70	5.89	137
1B	286	165	120.18	1.16	9.20	159
2B	38	38	65.78	2.62	18.75	34
3B	388	192	454.55	9.18	18.36	186
4B	3	3	13.77	2.59	12.81	1
5B	267	154	370.51	0.97	15.63	151
6B	345	181	128.90	1.32	2.64	175
7B	301	172	101.99	1.13	10.69	165
1D	15	15	40.50	1.76	29.53	12
2D	77	77	89.07	1.71	28.28	75
3D	47	47	123.16	0.13	1.37	38
4D	2	2	18.36	0.81	44.39	0
5D	32	32	82.96	0.39	6.64	27
6D	2	2	2.64	0.73	8.65	1
7D	61	61	68.76	1.73	5.57	56
total	2917	1987	2371.40	0.81	44.39	1900
A	1053	846	690.27	0.66	18.61	820
B	1628	905	1255.69	0.77	18.75	871
D	236	236	425.44	1.80	44.39	209

To verify this map, we used the sequences of 1987 bin markers in a BLASTn query to search the bread wheat genome (Chinese Spring) (Additional file 1: Table S1). All the markers can hit to the bread wheat genome sequence, and all the sequence of markers can be mapped to the same chromosome in the bread wheat genome sequence.

### Mapping of the *Pis1* gene

**Fig. 2:**
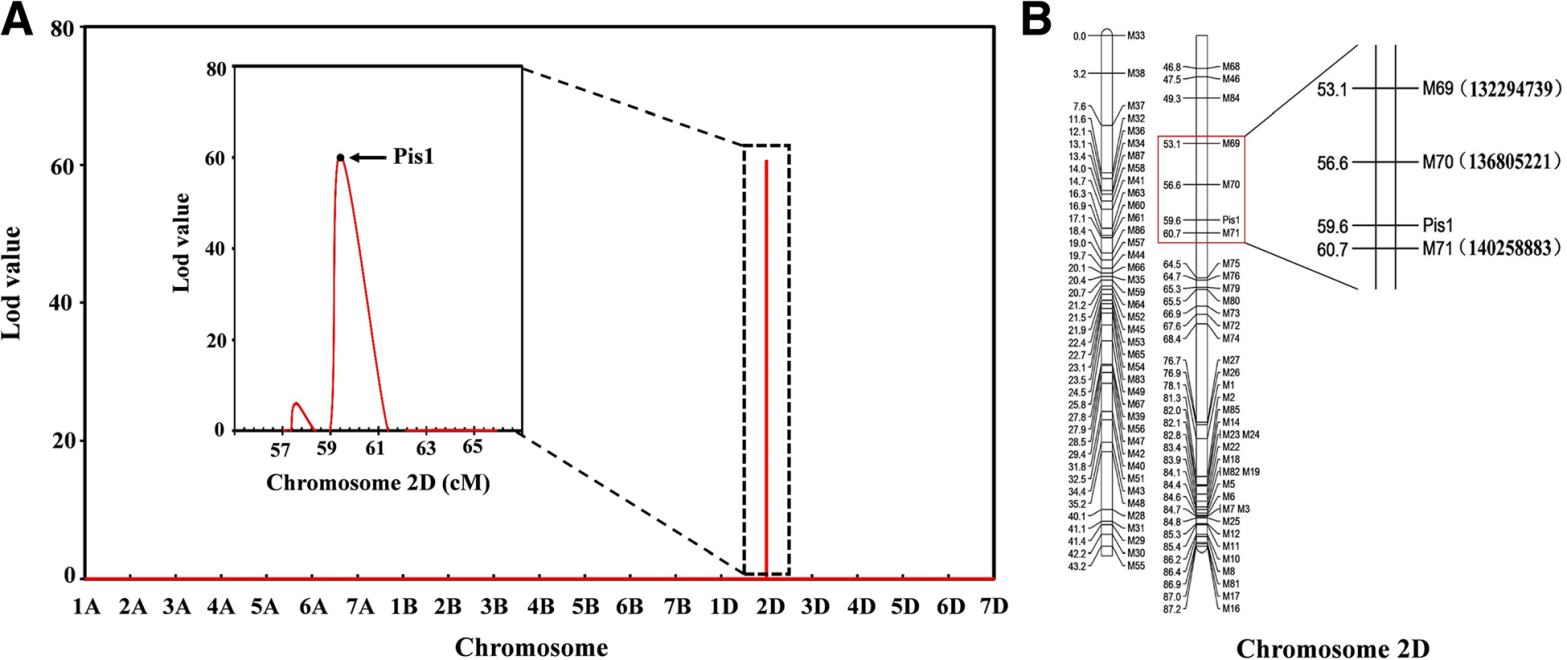
Detailed mapping of the *Pis1* gene on chromosome 2D. **a** The result of the QTL mapping of the three-pistil trait in wheat. Mapping QTL for plant height was conducted in the F_2_ population of CM28 and CM28TP in MapQTL6.0. In the chart, the red curve indicated LOD value at any genetic position. **b** The genetic linkage map of the *Pis1* gene. Genetic distances are indicated on the left side of linkage group in centiMorgans (cM), and the marker names are shown on the right side. The physical distances are shown in the brackets

To map *Pis1*, we recorded the number of pistils per floret for the 200 F_2_ individuals during the flowering period. The pistils were divided into two groups: three pistils or one pistil per floret. The ratio of three pistils to one pistil fit to 3:1 (χ^2^ = 0.96, *p* > 0.05), indicating that a single dominant gene controls the three-pistil trait. A high logarithm of the odds (LOD) score of 60.4 was detected for chromosome 2D (Fig. 2a). The genetic linkage map of chromosome 2D was constructed using pistil phenotypic and SNP data. Two markers were found to be tightly linked to the *Pis1* gene, namely, *M70* and *M71*, with genetic distances of 3 cM and 1.1 cM from *Pis1*, respectively. The physical distance of *M70* and *M71* is 3.4 Mb in the sequence of the bread wheat genome (Chinese Spring) (Fig. 2b).

To verify the GBS-generated genotypic data and to eliminate missing marker data in the *Pis1* region, five KASP markers were designed from corresponding GBS sequences harboring SNPs surrounding *Pis1* (Table 2). To avoid confusion with GBS-SNP markers, we tentatively designated the KASP-SNP markers as *KM69*, *KM70*, *KM71*, *KM75*, and *KM76*, which correspond with *M69, M70, M71, M75,* and *M70* in GBS-SNP markers, respectively. Four KASP-SNP markers were well amplified and polymorphic between the parents and in the F_2_ population. Three markers, *KM70*, *KM71*, and *KM75*, were remapped to the *Pis1* region. The KASP-SNP *KM69* is not linked to *Pis1*. A comparison of the GBS-SNP and KASP-SNP data revealed identical genotypes in the F_2_ population for *KM70*, *KM71*, and *KM75*. In contrast, the KASP-SNP marker *KM69* did not match the GBS-SNP data because of calling errors for GBS-SNP or KASP-SNP data in 114 F_2_ individuals.

**Table 2:** Primer sequences for the KASP assays of the GBS markers linked to the *Pis1* gene

Markers	Allele-specific 1 (FAM)	Allele-specific 2 (HEX)	Common
*KM69*	AACAAAAGCGGGTCCTCTCCG	GAACAAAAGCGGGTCCTCTCCA	CGTGCTGCTTCCTTCCAAGCCAT
*KM70*	ATCGCCAGATGCCACGCACAA	CGCCAGATGCCACGCACAG	AGGGACGTGATCAAATTTTCTTGACGATT
*KM71*	TTTGGACATTATTGGGCTTTATTATACAC	GTTTTGGACATTATTGGGCTTTATTATACAA	CTGGGTTAATAGGTTAGTCCCAAAAGTAA
*KM75*	GGTGCTCGCCCTAAACAATCACA	GTGCTCGCCCTAAACAATCACC	CAGTCCACGTGTCTTTTCTGTAAAACATT
*KM76*	GGCTGGCAGGCTCGCTCG	GGCTGGCAGGCTCGCTCT	TCCGTTGCAGCCGCCAAAGCAA

### Candidate gene prediction and functional annotation

**Fig. 3:**
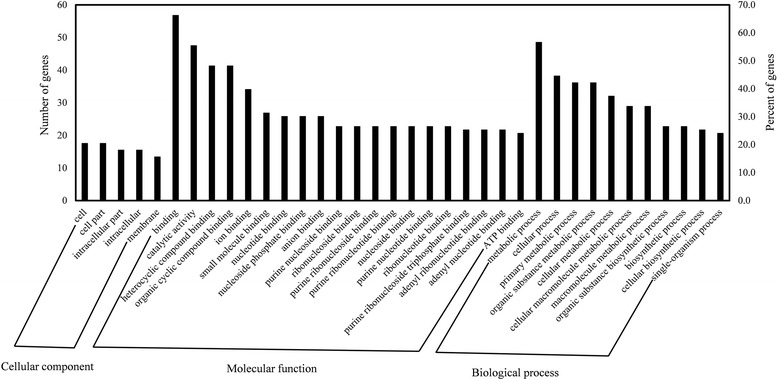
Gene Ontology (GO) annotations of the candidate genes

According to the wheat gene annotation database, Triticum_aestivum.IWGSC1+popseq.31.pep (ftp://ftp.ensemblgenomes.org/pub/plants/release-31/fasta/triticum_aestivum/pep/), the physical intervals of *M70* and *M71* encompass 127 protein-coding genes. Currently, 91 protein functions have been annotated in this region, among which 83 have Gene Ontology (GO) annotations (Additional file 1: Table S2). The results of GO analysis indicated that the number of genes in the different classifications varies: 107 genes were identified in the cellular component category, 552 genes in the molecular function category, and 326 genes in the biological process category. Several genes have multiple functions, and these were categorized into more than one function. In the cellular components category, 17 genes are related to the cell and 17 to cellular ontology. In the molecular functions category, 55 genes are associated with binding. In the biological processes category, 47 genes are relevant to metabolic processes (Fig. 3). Among the candidate genes within a 1.5-LOD decrease on either side of the peak bin that was delimited as the *Pis1* interval (the physical distance ranges from 139,248,092 bp to 139,416,092 bp), 10 genes were identified with the closest proximity to *Pis1*. Of these 10 genes, annotation information is available for 6 (Table 3). Among them, 3 are associated with molecular functions, 2 with cellular components, and only one with biological processes.

**Table 3:** The positions of the candidate genes nearest to *Pis1*

No.	Start position	End position	BLAST matching accession no.	Annotation	Sequence identity (%)	*E* value
1	139,244,852	139,245,784	EMT28401	N/A		
2	139,342,698	139,344,779	ACT22500	plastid glutamine synthetase 2 [*Triticum aestivum*]	98%	9e-66
3	139,348,585	139,351,702	EMS55336	DNA topoisomerase 1 [*Triticum urartu*]	80%	0
4	139,364,799	139,366,357	EMS51822	Transmembrane 9 superfamily member 4 [*Triticum urartu*]	97%	0
5	139,369,519	139,370,195	EMT08392	Tropinone reductase-like protein [*Aegilops tauschii*]	88%	4e-53
6	139,380,317	139,381,181	EMT03631	N/A		
7	139,385,118	139,386,329	EMS51820	N/A		
8	139,393,899	139,394,513	EMT03801	Transmembrane 9 superfamily member 2 [*Aegilops tauschii*]	98%	1e-93
9	139,406,363	139,408,447	EMT09611	U3 small nucleolar ribonucleoprotein IMP3 [*Aegilops tauschii*]	99%	2e-130
10	139,416,283	139,418,780	NP_001150358	N/A		

## Discussion

Although TP, a common wheat mutant, possesses normal spike morphology, it exhibits three pistils per floret; thus, TP has the potential for increased wheat production [6]. It has been demonstrated by genetic analysis that the TP trait is under the control of a single dominant nuclear gene, *Pis1*, which lies between SSR markers *Xgwm539* and *Xgwm349* on chromosome arm 2DL [7]. However, *Pis1* is too genetically distant from *Xgwm539* and *Xgwm349* to meet the requirements for fine-mapping. *Pis1* marker development has presented considerable challenges due to the relatively low level of DNA polymorphisms detected on chromosome 2D between wheat genotypes. Only 61 SSR markers are available for 2D, which is reflected in the low marker density of chromosome 2D linkage maps [14]. After exhaustively testing available SSR markers, none were sufficiently close to *Pis1* to ensure its reliable detection. Therefore, GBS was chosen as a new approach for mapping of the *Pis1* gene.

GBS is a recently developed, NGS-based genotyping approach that is cost-effective and highly accurate for high-density marker development [15]. The use of GBS markers is advantageous in several aspects. For instance, GBS has a general sample preparation method, a highly robust genome complexity reduction strategy to facilitate de novo marker discovery across entire genomes, and a uniform bioinformatics workflow strategy to achieve genotyping goals tailored to individual species that is independent of a reference sequence [12]. The most extraordinary attribute of GBS is the ability to genotype any population structure regardless of the availability of parental data and the ability to co-dominantly score SNP markers segregating in populations [12]. We utilized this novel genotyping method to map *Pis1* in an F_2_ population*.* The development and application of GBS markers in an F_2_ population are examples of the successful development of high-density markers in wheat, for which there is no sequenced genome available, and the determination of the physical location of a major gene without sequencing physical contigs or the complete wheat genome.

In this work, the benefits of the GBS protocol are exemplified by successful genotyping of an F_2_ population and generating abundant parental SNP information with a low cost and effort. The F_2_ population was derived from crossing CM28 with its near-isogenic line CM28TP. CM28 and CM28TP have similar phenotypes and genetic backgrounds, except for the three-pistil phenotype [8]. Polymorphisms were minimal in this F_2_ population; however, we believe that sequencing of this population could be used to identify markers closely linked to *Pis1*. In total, 727,097 and 679,440 SNPs were identified in CM28TP and CM28, respectively, and 2917 selected polymorphic markers were eventually identified for the construction of a high-density linkage map. The 2917 SNPs cover all 21 linkage groups, spanning a total length of 2371.40 cM. To the best of our knowledge, this genetic map represents the densest map in wheat thus far. The mean distance between two neighboring markers, 0.81 cM, is the least compared with the previously reported mean distances of 0.88 cM and 0.86 cM [16, 17]. However, D genome chromosomes were less well represented in this study. In particular, only two markers were found for 4D and 6D. This poor representation possibly resulted from low polymorphisms of the D genome [18, 19].

The development of closely linked markers and fine-mapping is an essential initial step in the map-based cloning of *Pis1*. In the present study, we mapped *Pis1* using a genetic linkage map that we had constructed. *Pis1* is located in a 4.1 cM region and is flanked by *M70 and M71*. Sequencing-based genotyping technology may result in a large amount of missing data, and even GBS cannot avoid this shortcoming. Therefore, it is suggested that the method of imputation be applied to predict genotypes with missing data [12, 13, 20, 21]. Utilizing high-quality SNPs with <20% missing data without imputation is an alternative for improving the data quality [22], though this approach will likely lead to some essential SNPs being overlooked. During this research, we utilized GBS-SNP with <25% missing data to construct a map for delineating *Pis1*. To verify the GBS-SNP data, we then converted GBS-SNPs closely linked to *Pis1* to KASP-SNPs. With this approach, the negative effect caused by missing data and corrected sequencing error can be minimized, therefore improving the accuracy of the results for *Pis1* mapping. Among the five designed KASP assays, were successful in the F_2_ population, three corresponded to GBS-SNP calls (markers *KM70*, *KM71*, and *KM75*), and one had SNP call errors. These errors are likely to occur in sequencing and during the SNP-calling pipeline. Therefore, we can conclude that the *Pis1* gene location is accurate. *M70* and *M71* have a physical distance of 3.4 Mb in the sequence of the bread wheat genome and encompass 127 protein-coding genes. Although the relationship between the 127 identified genes and the three-pistil trait requires further research, our study on mapping of the *Pis1* gene lays the foundation for the map-based cloning of *Pis1.*

## Conclusions

In the present study, a high-density linkage map of wheat was constructed using GBS-SNP data from an F_2_ mapping population (CM28 × CM28TP). The map spans a total length of 2371.40 cM, and individual chromosomes range from 2.64 cM to 454.55 cM. The average distance is 0.81 cM. Using this map, the *Pis1* gene was mapped, and two markers tightly linked to the *Pis1* gene, *M70* and *M71*, were identified. The *Pis1* gene is located between SNP markers *M70* and *M71* and is 3 cM from *M70* and 1.1 cM from *M71*. *M70* and *M71* have a physical distance of 3.4 Mb in the draft sequence of the bread wheat genome, which encompasses 127 protein-coding genes. To validate the GBS-generated genotypic data and to eliminate missing marker data in the *Pis1* region, five KASP markers were designed from corresponding GBS sequences harboring SNPs surrounding *Pis1*. Three KASP-SNP markers, *KM70*, *KM71*, and *KM75*, were remapped to the *Pis1* gene region. This work not only lays the foundation for the map-based cloning of *Pis1* but can also serve as a valuable tool for studying marker-trait association of important traits and marker-assisted breeding in wheat.

## Methods

### Plant materials and DNA isolation

This study utilized a near-isogenic line, CM28TP (carrying the *Pis1* gene), and its recurrent parent Chuanmai 28 (CM28). CM28TP has a similar phenotypic to CM28, except for the three-pistil trait [8]. An F_2_ population of 200 individuals derived from the cross between CM28 and CM28TP was used to construct the map. All plants were maintained at the China West Normal University in Nanchong, China. Fresh leaves from the parents and F_2_ individuals were collected for DNA isolation. Samples were lyophilized and stored at −80 °C until use. Total genomic DNA was isolated with Plant Genomics DNA Kit (TIANGEN Biotech) in accordance with the manufacturer’s recommendations. The quality and concentration of the genomic DNA were assessed by agarose gel electrophoresis and using an ND-2000C spectrophotometer (NanoDrop).

### Genotyping-by-sequencing (GBS) approach

DNA from the 200 F_2_ plants and two parental plants was subjected to GBS according to Elshire et al. [23]. Specifically, 200 ng of DNA from each sample was digested with a combination of *Mse*I, *Hae*III, and *Eco*RI restriction enzymes, and barcoded forward adapters and common reverse adapters were ligated to the digested fragments. A total of 202 samples were included in one 202-plex library. The PCR reactions were performed using Illumina primers with sequences complimentary to the adapters used during the library preparation. The PCR products were sent to Beijing Novogene Bioinformatics Technology Co., Ltd. for next-generation sequencing (NGS) using an Illumina Hi-seq 2000 platform. One library was double-loaded onto two lanes of the Illumina flow cell as technical replicates. Sequencing data from our study were submitted to National Center for Biotechnology Information (NCBI) under the accession number SRP080791. The GBS sequences were analyzed using the bioinformatics pipeline UNEAK [24], after which the tag sequence was converted into genotypic calls. Polymorphic parental GBS-SNPs were classified into eight segregation patterns, lm × ll, ab × cd, hk × hk, ab × cc, ef × eg, cc × ab, aa × bb and nn × np; but aa and bb were considered for the F_2_ population. To ensure linkage map quality, the GBS-SNPs markers used for the final mapping were selected by removing markers with significant distortion (Chi-square 1:2:1 test *p* < 0.001) and more than 25% missing data.

### Genetic map construction and mapping of *Pis1*

JoinMap Version 4.0 was utilized to construct the linkage map with GBS-SNP data [25]. Map construction excluded markers with obvious segregation distortion from the expected Mendelian segregation ratios of 1:2:1. Markers were positioned on linkage groups based on independence LOD threshold values of 2.0–12.0. We selected the JoinMap ‘similarity of loci’ command to identify identical markers (similarity value = 1.000), which should be mapped to the same position on the linkage group. To reduce the calculation burden, only one marker of ‘similar loci’ was retained on the linkage map. Linkage analysis and marker order assignment were carried out using the regression mapping algorithm. Recombination fractions between markers were converted to map distances in cM using the Kosambi mapping function [26]. The final map included bin markers (excluding similar SNPs markers). The linkage maps were drawn using MapChart 2.2 [27]. All bin markers were compared with the bread wheat genome sequence, IWGSC1+popseq.31.pep (ftp://ftp.ensemblgenomes.org/pub/plants/release-31/fasta/triticum_aestivum/pep/), by BLASTn.

To map *Pis1*, we assumed that the *Pis1* locus is a quantitative trait locus (QTL) site and then used QTL analysis to locate *Pis1*. QTLs for the three-pistil phenotype were identified using the Interval Mapping program by MapQTL 6.0 [28]. The LOD threshold for significance for the presence of a putative QTL was 2.5. QTL peak positions were estimated using maximum LOD values. The phenotypic and SNP markers for three pistils on the 2D chromosome were then combined to map *Pis1*.

### Kompetitive allele-specific PCR (KASP) assay and candidate gene identification

KASP genotyping assays were developed for GBS-SNPs surrounding the *Pis1* gene. Two allele-specific forward primers and one common reverse primer were designed for each KASP assay (Table 2). The KASP assays were carried out in 96-well PCR plates in a 5-μL final volume consisting of 2.5 μL KASP 2× Reaction Mix, 0.07 μL assay mix [12 μL each allele-specific forward primer (100 μM), 30 μL reverse primer (100 μM), and 46 μL TRIS (10 mM, pH 8.3)], and 50 ng genomic DNA. The PCR program was performed under the following conditions: 94 °C for 15 min; 10 touchdown cycles of 94 °C for 20 s, 65–57 °C for 60 s (decreasing by 0.8 °C per cycle); and 32 cycles of 94 °C for 20 s, 57 °C for 60 s. Fluorescence detection of the PCR reactions was carried out with a Bio-Rad CFX96 real-time PCR platform (Bio-Rad Laboratories, Hercules, CA, USA). KlusterCaller software (LGC Genomics, Beverly, USA) was applied to analyze the data.

The sequences of the markers flanking the *Pis1* intervals were aligned back to the bread wheat genome sequence IWGSC1+popseq.31.pep (ftp://ftp.ensemblgenomes.org/pub/plants/release-31/fasta/triticum_aestivum/pep/). All genes within the interval were identified as candidate genes based on the position of the flanking markers. All candidate genes were categorized by GO analysis (http://www.geneontology.org/).

## Additional file

Additional file 1: Table S1. GBS makers and their BLAST hit information. **Table S2** Genes located in the intervals of *Pis1*. **Figure S1** High-density genetic map marker information. (DOCX 1044 kb)

## Abbreviations

CM28: Chuanmai 28
GBS: Genotyping-by-sequencing
KASP: Kompetitive allele-specific PCR
MSG: Multiplexed shotgun sequencing
NGS: Next-generation sequencing
RAD-seq: Restriction-site-associated DNA sequencing
SNP: Single-nucleotide polymorphism
SSR: Simple sequence repeat
TP: Wheat mutant line three pistil
