# Supplementary material for: Development of a high-density linkage map and mapping of the three-pistil gene (Pis1) in wheat using GBS markers
Source: BMC Genomics. 2017 Jul 31;18:567. doi: 10.1186/s12864-017-3960-7 (PMC5537994; doi:10.1186/s12864-017-3960-7)
Supplement: Additional file 1: Table S1. — GBS makers and their BLAST hit information. Table S2 Genes located in the intervals of Pis1. Figure S1 High-density genetic map marker information. (DOCX 1044 kb) [file 12864_2017_3960_MOESM1_ESM.docx]

Table S1 GBS makers and their BLAST hit information

| linkage group No. | name of markers | genetic distance | chromosome | physical distance |
| --- | --- | --- | --- | --- |
| lg1 | M186 | 0 | IWGSC_CSS_1AL_scaff_3980640 | 820 |
| lg1 | M43 | 1.481 | 1A | 193745356 |
| lg1 | M18 | 2.975 | IWGSC_CSS_1AL_scaff_3889110 | 1420 |
| lg1 | M38 | 3.666 | 1A | 182655571 |
| lg1 | M42 | 4.323 | 1A | 188142228 |
| lg1 | M37 | 4.908 | 1A | 180271502 |
| lg1 | M39 | 5.724 | 1A | 186804633 |
| lg1 | M24 | 6.468 | IWGSC_CSS_1AL_scaff_3953631 | 2121 |
| lg1 | M196 | 7.231 | IWGSC_CSS_1AL_scaff_3947372 | 2842 |
| lg1 | M20 | 7.53 | IWGSC_CSS_1AL_scaff_3975207 | 1322 |
| lg1 | M49 | 7.824 | 1A | 207467960 |
| lg1 | M53 | 8.038 | 1A | 208206940 |
| lg1 | M44 | 8.327 | 1A | 206431113 |
| lg1 | M45 | 8.339 | 1A | 206431165 |
| lg1 | M46 | 8.465 | 1A | 206431392 |
| lg1 | M54 | 8.844 | 1A | 208207245 |
| lg1 | M50 | 9.034 | 1A | 207468141 |
| lg1 | M19 | 9.389 | IWGSC_CSS_1AL_scaff_3904084 | 325 |
| lg1 | M185 | 9.629 | IWGSC_CSS_1AL_scaff_3980716 | 4327 |
| lg1 | M56 | 9.694 | 1A | 209729603 |
| lg1 | M47 | 9.887 | 1A | 206542936 |
| lg1 | M48 | 9.902 | 1A | 206542978 |
| lg1 | M78 | 10 | 1A | 213320798 |
| lg1 | M77 | 10.05 | 1A | 213320732 |
| lg1 | M79 | 10.22 | 1A | 213871435 |
| lg1 | M88 | 10.552 | 1A | 215207083 |
| lg1 | M89 | 10.698 | 1A | 215207149 |
| lg1 | M87 | 10.75 | 1A | 215206941 |
| lg1 | M60 | 11.094 | 1A | 210038690 |
| lg1 | M61 | 11.239 | 1A | 210038734 |
| lg1 | M57 | 11.468 | 1A | 210038539 |
| lg1 | M58 | 11.567 | 1A | 210038559 |
| lg1 | M55 | 11.887 | 1A | 208336485 |
| lg1 | M64 | 12.394 | 1A | 210482446 |
| lg1 | M66 | 12.971 | 1A | 210482600 |
| lg1 | M65 | 13.136 | 1A | 210482481 |
| lg1 | M74 | 13.776 | 1A | 212789042 |
| lg1 | M73 | 13.838 | 1A | 212788889 |
| lg1 | M72 | 14.024 | 1A | 211275236 |
| lg1 | M90 | 14.249 | 1A | 215379074 |
| lg1 | M82 | 14.453 | 1A | 214499333 |
| lg1 | M81 | 14.467 | 1A | 214499314 |
| lg1 | M83 | 14.66 | 1A | 214499361 |
| lg1 | M92 | 15.193 | 1A | 215994495 |
| lg1 | M1 | 15.834 | IWGSC_CSS_1AL_scaff_3644839 | 701 |
| lg1 | M86 | 16.041 | 1A | 215155789 |
| lg1 | M184 | 16.207 | IWGSC_CSS_1AL_scaff_3974500 | 305 |
| lg1 | M25 | 16.645 | IWGSC_CSS_1AL_scaff_3869162 | 1051 |
| lg1 | M21 | 17.017 | IWGSC_CSS_1AL_scaff_3975710 | 2936 |
| lg1 | M91 | 17.415 | 1A | 215800090 |
| lg1 | M96 | 18.054 | 1A | 219725641 |
| lg1 | M98 | 18.089 | 1A | 219725819 |
| lg1 | M99 | 18.804 | 1A | 220854480 |
| lg1 | M95 | 19.169 | 1A | 218723505 |
| lg1 | M93 | 19.839 | 1A | 218435468 |
| lg1 | M100 | 20.438 | 1A | 220962636 |
| lg1 | M94 | 21.461 | 1A | 218719068 |
| lg1 | M85 | 23.189 | 1A | 214838128 |
| lg1 | M84 | 23.432 | 1A | 214838095 |
| lg1 | M109 | 25.346 | 1A | 225303831 |
| lg1 | M106 | 25.714 | 1A | 224917523 |
| lg1 | M107 | 25.792 | 1A | 224917741 |
| lg1 | M110 | 26.001 | 1A | 225491497 |
| lg1 | M103 | 26.546 | 1A | 224732187 |
| lg1 | M112 | 26.868 | 1A | 225543583 |
| lg1 | M102 | 27.188 | 1A | 223204292 |
| lg1 | M101 | 27.302 | 1A | 223204255 |
| lg1 | M125 | 28.814 | 1A | 228123997 |
| lg1 | M126 | 29.051 | 1A | 228124029 |
| lg1 | M114 | 29.99 | 1A | 227164486 |
| lg1 | M117 | 32.035 | 1A | 227566735 |
| lg1 | M115 | 32.148 | 1A | 227566562 |
| lg1 | M4 | 32.728 | IWGSC_CSS_1AL_scaff_3872633 | 835 |
| lg1 | M3 | 32.97 | IWGSC_CSS_1AL_scaff_3872633 | 790 |
| lg1 | M191 | 33.276 | IWGSC_CSS_1AL_scaff_3917752 | 1379 |
| lg1 | M187 | 33.435 | IWGSC_CSS_1AL_scaff_3976865 | 1562 |
| lg1 | M5 | 33.657 | IWGSC_CSS_1AL_scaff_3872633 | 997 |
| lg1 | M188 | 33.829 | IWGSC_CSS_1AL_scaff_3948301 | 863 |
| lg1 | M145 | 34.012 | 1A | 230681226 |
| lg1 | M143 | 34.246 | 1A | 230632750 |
| lg1 | M118 | 34.387 | 1A | 228108442 |
| lg1 | M22 | 34.557 | IWGSC_CSS_1AL_scaff_3883490 | 4319 |
| lg1 | M23 | 34.581 | IWGSC_CSS_1AL_scaff_3883490 | 4520 |
| lg1 | M194 | 34.858 | IWGSC_CSS_1AL_scaff_3875674 | 2435 |
| lg1 | M195 | 34.879 | IWGSC_CSS_1AL_scaff_3875674 | 2510 |
| lg1 | M113 | 35.037 | 1A | 226975001 |
| lg1 | M190 | 35.086 | IWGSC_CSS_1AL_scaff_3894482 | 582 |
| lg1 | M119 | 35.227 | 1A | 228108617 |
| lg1 | M129 | 35.386 | 1A | 229716486 |
| lg1 | M139 | 35.619 | 1A | 229979744 |
| lg1 | M132 | 36.2 | 1A | 229896419 |
| lg1 | M133 | 36.241 | 1A | 229896583 |
| lg1 | M146 | 36.993 | 1A | 231815904 |
| lg1 | M147 | 37.185 | 1A | 231816147 |
| lg1 | M128 | 38.34 | 1A | 229699840 |
| lg1 | M127 | 38.393 | 1A | 229699584 |
| lg1 | M17 | 39.538 | IWGSC_CSS_1AL_scaff_3943139 | 1858 |
| lg1 | M14 | 39.89 | IWGSC_CSS_1AL_scaff_3943139 | 1690 |
| lg1 | M13 | 40.037 | IWGSC_CSS_1AL_scaff_3943139 | 1633 |
| lg1 | M15 | 40.34 | IWGSC_CSS_1AL_scaff_3943139 | 1805 |
| lg1 | M142 | 41.073 | 1A | 230590974 |
| lg1 | M140 | 41.614 | 1A | 230590727 |
| lg1 | M141 | 41.63 | 1A | 230590764 |
| lg1 | M130 | 42.675 | 1A | 229746975 |
| lg1 | M149 | 45.183 | 1A | 232335751 |
| lg1 | M189 | 45.819 | IWGSC_CSS_1AL_scaff_3904351 | 2686 |
| lg1 | M150 | 46.194 | 1A | 232362003 |
| lg1 | M152 | 46.738 | 1A | 232413709 |
| lg1 | M154 | 47.068 | 1A | 232413946 |
| lg1 | M33 | 47.181 | 1A | 5269076 |
| lg1 | M155 | 47.405 | 1A | 233134723 |
| lg1 | M156 | 47.817 | 1A | 233850054 |
| lg1 | M11 | 48.246 | IWGSC_CSS_1AL_scaff_3874570 | 6447 |
| lg1 | M10 | 48.311 | IWGSC_CSS_1AL_scaff_3874570 | 6201 |
| lg1 | M148 | 49.154 | 1A | 232168657 |
| lg1 | M157 | 49.709 | 1A | 234285476 |
| lg1 | M158 | 50.412 | 1A | 234285626 |
| lg1 | M161 | 50.414 | 1A | 234285718 |
| lg1 | M159 | 50.431 | 1A | 234285652 |
| lg2 | M30 | 0 | 2A | 14914060 |
| lg2 | M31 | 0.248 | 2A | 14914256 |
| lg2 | M8 | 1.36 | IWGSC_CSS_2AS_scaff_5207226 | 1287 |
| lg2 | M20 | 1.924 | 2A | 11720853 |
| lg2 | M19 | 2.487 | 2A | 11238324 |
| lg2 | M25 | 4.397 | 2A | 12696856 |
| lg2 | M27 | 4.461 | 2A | 12697158 |
| lg2 | M23 | 5.143 | 2A | 12497491 |
| lg2 | M22 | 5.327 | 2A | 12497248 |
| lg2 | M21 | 5.908 | 2A | 12042056 |
| lg2 | M28 | 7.131 | 2A | 12822213 |
| lg2 | M29 | 8.034 | 2A | 13404480 |
| lg2 | M186 | 23.66 | IWGSC_CSS_2AS_scaff_5236002 | 2237 |
| lg2 | M33 | 25.447 | 2A | 17432226 |
| lg2 | M7 | 28.966 | IWGSC_CSS_2AS_scaff_5230825 | 3097 |
| lg2 | M48 | 33.889 | 2A | 129068391 |
| lg2 | M86 | 35.288 | 2A | 220965556 |
| lg2 | M85 | 35.471 | 2A | 220965531 |
| lg2 | M71 | 36.568 | 2A | 208874434 |
| lg2 | M70 | 37.031 | 2A | 208835065 |
| lg2 | M76 | 37.722 | 2A | 209094351 |
| lg2 | M77 | 37.762 | 2A | 209094576 |
| lg2 | M87 | 38.656 | 2A | 220978026 |
| lg2 | M69 | 42.142 | 2A | 200556913 |
| lg2 | M43 | 46.338 | 2A | 117465332 |
| lg2 | M56 | 49.139 | 2A | 165650575 |
| lg2 | M58 | 49.139 | 2A | 165650647 |
| lg2 | M57 | 49.188 | 2A | 165650580 |
| lg2 | M59 | 49.239 | 2A | 165650830 |
| lg2 | M67 | 49.77 | 2A | 192905677 |
| lg2 | M68 | 49.843 | 2A | 192905745 |
| lg2 | M49 | 50.174 | 2A | 140776385 |
| lg2 | M65 | 50.767 | 2A | 191103876 |
| lg2 | M66 | 50.788 | 2A | 191103930 |
| lg2 | M54 | 51.081 | 2A | 161974963 |
| lg2 | M52 | 51.121 | 2A | 161974725 |
| lg2 | M35 | 51.683 | 2A | 30786791 |
| lg2 | M37 | 51.956 | 2A | 30786805 |
| lg2 | M44 | 52.522 | 2A | 120471542 |
| lg2 | M46 | 53.336 | 2A | 120471757 |
| lg2 | M47 | 56.926 | 2A | 127990249 |
| lg2 | M61 | 58.337 | 2A | 172606909 |
| lg2 | M64 | 59.626 | 2A | 185598366 |
| lg2 | M50 | 60.008 | 2A | 147042652 |
| lg2 | M51 | 60.695 | 2A | 159223629 |
| lg2 | M42 | 63.463 | 2A | 46531274 |
| lg2 | M78 | 70.306 | 2A | 211979193 |
| lg2 | M41 | 71.913 | 2A | 44179357 |
| lg2 | M83 | 75.982 | 2A | 219801293 |
| lg2 | M79 | 76.494 | 2A | 219801030 |
| lg2 | M81 | 76.591 | 2A | 219801092 |
| lg2 | M82 | 76.622 | 2A | 219801127 |
| lg2 | M96 | 76.919 | 2A | 229030493 |
| lg2 | M97 | 77.063 | 2A | 229030542 |
| lg2 | M94 | 77.611 | 2A | 221938252 |
| lg2 | M98 | 78.499 | 2A | 229850301 |
| lg2 | M99 | 78.639 | 2A | 229850446 |
| lg2 | M91 | 79.83 | 2A | 221102422 |
| lg2 | M89 | 79.88 | 2A | 221102182 |
| lg2 | M107 | 88.653 | 2A | 235050619 |
| lg2 | M100 | 90.574 | 2A | 230818569 |
| lg2 | M110 | 91.08 | 2A | 236030897 |
| lg2 | M5 | 91.902 | IWGSC_CSS_2AL_scaff_6403345 | 1148 |
| lg2 | M6 | 91.934 | IWGSC_CSS_2AL_scaff_6403345 | 1208 |
| lg2 | M2 | 91.993 | IWGSC_CSS_2AL_scaff_6403345 | 959 |
| lg2 | M14 | 92.902 | IWGSC_CSS_2AL_scaff_6436859 | 3297 |
| lg2 | M16 | 93.035 | IWGSC_CSS_2AL_scaff_6436859 | 3454 |
| lg2 | M15 | 93.127 | IWGSC_CSS_2AL_scaff_6436859 | 3349 |
| lg2 | M109 | 93.552 | 2A | 235851835 |
| lg2 | M111 | 93.8 | 2A | 236105031 |
| lg2 | M13 | 93.922 | IWGSC_CSS_2AL_scaff_6434334 | 3121 |
| lg2 | M108 | 94.06 | 2A | 235058475 |
| lg2 | M105 | 94.51 | 2A | 234914823 |
| lg2 | M103 | 94.872 | 2A | 234078273 |
| lg2 | M102 | 95.021 | 2A | 234078039 |
| lg2 | M112 | 95.429 | 2A | 236290084 |
| lg2 | M106 | 95.692 | 2A | 234966888 |
| lg2 | M104 | 96.269 | 2A | 234801536 |
| lg2 | M101 | 97.032 | 2A | 232099966 |
| lg2 | M121 | 100.279 | 2A | 239085188 |
| lg2 | M113 | 101.008 | 2A | 237675424 |
| lg2 | M118 | 101.537 | 2A | 238733540 |
| lg2 | M119 | 101.871 | 2A | 238825383 |
| lg2 | M120 | 102.24 | 2A | 238825466 |
| lg2 | M114 | 102.57 | 2A | 237791965 |
| lg2 | M116 | 103.624 | 2A | 238712155 |
| lg2 | M115 | 103.948 | 2A | 238712110 |
| lg2 | M117 | 104.106 | 2A | 238712358 |
| lg2 | M126 | 107.754 | 2A | 241214546 |
| lg2 | M165 | 110.5 | 2A | 246347524 |
| lg2 | M177 | 111.789 | 2A | 252558487 |
| lg2 | M174 | 112.079 | 2A | 251810617 |
| lg2 | M149 | 112.684 | 2A | 244025117 |
| lg2 | M128 | 113.196 | 2A | 241597406 |
| lg2 | M127 | 113.442 | 2A | 241597356 |
| lg2 | M158 | 113.712 | 2A | 245966585 |
| lg2 | M159 | 113.873 | 2A | 245966690 |
| lg2 | M160 | 113.893 | 2A | 245966756 |
| lg2 | M130 | 114.216 | 2A | 242262699 |
| lg2 | M148 | 114.421 | 2A | 243473176 |
| lg2 | M147 | 114.548 | 2A | 243473112 |
| lg2 | M131 | 114.698 | 2A | 242262794 |
| lg2 | M132 | 114.755 | 2A | 242262839 |
| lg2 | M129 | 114.835 | 2A | 242068179 |
| lg2 | M135 | 115.093 | 2A | 242942017 |
| lg2 | M136 | 115.457 | 2A | 243126997 |
| lg2 | M157 | 115.598 | 2A | 244817968 |
| lg2 | M156 | 115.633 | 2A | 244817743 |
| lg2 | M146 | 115.726 | 2A | 243440917 |
| lg2 | M122 | 115.811 | 2A | 240022253 |
| lg2 | M123 | 115.851 | 2A | 240022447 |
| lg2 | M125 | 115.876 | 2A | 240022480 |
| lg2 | M139 | 116.106 | 2A | 243235387 |
| lg2 | M137 | 116.129 | 2A | 243235346 |
| lg2 | M140 | 116.14 | 2A | 243235621 |
| lg2 | M143 | 116.328 | 2A | 243362544 |
| lg2 | M141 | 116.333 | 2A | 243362493 |
| lg2 | M152 | 116.998 | 2A | 244814542 |
| lg2 | M153 | 117.135 | 2A | 244814681 |
| lg2 | M175 | 118.225 | 2A | 252167232 |
| lg2 | M150 | 118.538 | 2A | 244228896 |
| lg2 | M151 | 118.549 | 2A | 244228938 |
| lg2 | M166 | 119.409 | 2A | 246610335 |
| lg2 | M185 | 120.269 | IWGSC_CSS_2AL_scaff_6366354 | 14795 |
| lg2 | M161 | 120.837 | 2A | 246261220 |
| lg2 | M182 | 123.35 | 2A | 253549323 |
| lg2 | M184 | 123.939 | 2A | 253906458 |
| lg2 | M183 | 124.132 | 2A | 253906295 |
| lg2 | M172 | 124.384 | 2A | 250789136 |
| lg2 | M169 | 124.891 | 2A | 250446907 |
| lg2 | M179 | 126.185 | 2A | 252814870 |
| lg2 | M180 | 128.018 | 2A | 253471033 |
| lg3 | M10 | 0 | 3A | 2404840 |
| lg3 | M16 | 2.06 | 3A | 3489033 |
| lg3 | M13 | 2.716 | 3A | 3488805 |
| lg3 | M14 | 2.782 | 3A | 3488854 |
| lg3 | M103 | 3.717 | IWGSC_CSS_3AS_scaff_3275733 | 2052 |
| lg3 | M11 | 4.851 | 3A | 3488158 |
| lg3 | M12 | 4.929 | 3A | 3488185 |
| lg3 | M5 | 6.039 | 3A | 2108936 |
| lg3 | M4 | 6.599 | 3A | 1913924 |
| lg3 | M3 | 7.078 | 3A | 1913729 |
| lg3 | M17 | 11.484 | 3A | 4137924 |
| lg3 | M32 | 15.815 | 3A | 5203221 |
| lg3 | M31 | 16.076 | 3A | 5203157 |
| lg3 | M33 | 16.175 | 3A | 5203395 |
| lg3 | M23 | 16.935 | 3A | 4892831 |
| lg3 | M20 | 17.229 | 3A | 4892653 |
| lg3 | M24 | 17.306 | 3A | 4892871 |
| lg3 | M19 | 18.571 | 3A | 4512299 |
| lg3 | M18 | 18.61 | 3A | 4512216 |
| lg3 | M27 | 19.544 | 3A | 5075735 |
| lg3 | M30 | 19.974 | 3A | 5075907 |
| lg3 | M25 | 21.138 | 3A | 5068103 |
| lg3 | M36 | 35.073 | 3A | 8017117 |
| lg3 | M35 | 40.629 | 3A | 7979730 |
| lg3 | M37 | 42.184 | 3A | 8440952 |
| lg3 | M38 | 42.24 | 3A | 8440970 |
| lg3 | M34 | 43.448 | 3A | 7979720 |
| lg3 | M2 | 48.966 | IWGSC_CSS_3AS_scaff_3441619 | 2204 |
| lg3 | M1 | 49.003 | IWGSC_CSS_3AS_scaff_3441619 | 2145 |
| lg3 | M40 | 50.174 | 3A | 10712520 |
| lg3 | M43 | 52.876 | 3A | 13867599 |
| lg3 | M41 | 53.648 | 3A | 10874387 |
| lg3 | M39 | 54.433 | 3A | 9145854 |
| lg3 | M42 | 55.483 | 3A | 13043066 |
| lg3 | M105 | 56.309 | IWGSC_CSS_3AS_scaff_3346353 | 2109 |
| lg3 | M47 | 69.499 | 3A | 83881126 |
| lg3 | M46 | 72.956 | 3A | 57937185 |
| lg3 | M44 | 74.38 | 3A | 49183043 |
| lg3 | M45 | 76.613 | 3A | 54219494 |
| lg3 | M108 | 106.142 | IWGSC_CSS_3AL_scaff_4452625 | 1263 |
| lg3 | M114 | 107.06 | IWGSC_CSS_3AL_scaff_4330433 | 1866 |
| lg3 | M111 | 107.433 | IWGSC_CSS_3AL_scaff_4330433 | 1656 |
| lg3 | M113 | 107.719 | IWGSC_CSS_3AL_scaff_4330433 | 1691 |
| lg3 | M48 | 108.536 | 3A | 156266866 |
| lg3 | M49 | 109.388 | 3A | 156947919 |
| lg3 | M50 | 110.136 | 3A | 157661268 |
| lg3 | M56 | 114.329 | 3A | 160966625 |
| lg3 | M107 | 115.221 | IWGSC_CSS_3AL_scaff_4449394 | 556 |
| lg3 | M52 | 115.298 | 3A | 158362452 |
| lg3 | M51 | 115.321 | 3A | 158362403 |
| lg3 | M54 | 115.421 | 3A | 159266985 |
| lg3 | M53 | 116.721 | 3A | 158702206 |
| lg3 | M64 | 116.995 | 3A | 161967705 |
| lg3 | M63 | 117.067 | 3A | 161967548 |
| lg3 | M57 | 117.219 | 3A | 161382170 |
| lg3 | M59 | 117.414 | 3A | 161382454 |
| lg3 | M58 | 117.484 | 3A | 161382397 |
| lg3 | M65 | 118.015 | 3A | 162394628 |
| lg3 | M55 | 118.265 | 3A | 160650872 |
| lg3 | M66 | 118.795 | 3A | 163069403 |
| lg3 | M69 | 118.802 | 3A | 163069678 |
| lg3 | M62 | 124.825 | 3A | 161773528 |
| lg3 | M73 | 126.178 | 3A | 163666943 |
| lg3 | M78 | 126.626 | 3A | 164197365 |
| lg3 | M80 | 126.896 | 3A | 164197551 |
| lg3 | M81 | 126.98 | 3A | 164197622 |
| lg3 | M71 | 127.228 | 3A | 163484058 |
| lg3 | M76 | 127.502 | 3A | 164039163 |
| lg3 | M75 | 127.672 | 3A | 164038986 |
| lg3 | M74 | 128.558 | 3A | 164024396 |
| lg3 | M85 | 129.394 | 3A | 164232538 |
| lg3 | M84 | 129.431 | 3A | 164232473 |
| lg3 | M87 | 130.524 | 3A | 165778249 |
| lg3 | M88 | 131.149 | 3A | 165778417 |
| lg3 | M70 | 131.82 | 3A | 163370966 |
| lg4 | M117 | 0 | 4A | 205234583 |
| lg4 | M130 | 0.688 | 4A | 207599888 |
| lg4 | M100 | 1.525 | 4A | 203225110 |
| lg4 | M109 | 1.819 | 4A | 204686758 |
| lg4 | M99 | 2.168 | 4A | 202431556 |
| lg4 | M128 | 2.485 | 4A | 206101588 |
| lg4 | M129 | 2.496 | 4A | 206101657 |
| lg4 | M127 | 2.683 | 4A | 206101414 |
| lg4 | M95 | 2.945 | 4A | 202295391 |
| lg4 | M132 | 3.286 | 4A | 208045780 |
| lg4 | M120 | 3.477 | 4A | 205502647 |
| lg4 | M107 | 3.574 | 4A | 204440979 |
| lg4 | M114 | 3.814 | 4A | 205007009 |
| lg4 | M115 | 3.869 | 4A | 205007035 |
| lg4 | M116 | 3.971 | 4A | 205007181 |
| lg4 | M102 | 4.092 | 4A | 203287791 |
| lg4 | M111 | 4.191 | 4A | 204750877 |
| lg4 | M110 | 4.191 | 4A | 204750799 |
| lg4 | M101 | 4.268 | 4A | 203287500 |
| lg4 | M96 | 4.368 | 4A | 202295592 |
| lg4 | M108 | 4.479 | 4A | 204592963 |
| lg4 | M124 | 4.653 | 4A | 205811441 |
| lg4 | M104 | 4.905 | 4A | 204311072 |
| lg4 | M106 | 4.955 | 4A | 204311177 |
| lg4 | M123 | 5.274 | 4A | 205564268 |
| lg4 | M122 | 5.476 | 4A | 205564255 |
| lg4 | M126 | 5.759 | 4A | 205957242 |
| lg4 | M125 | 5.873 | 4A | 205956999 |
| lg4 | M93 | 6.121 | 4A | 201281834 |
| lg4 | M146 | 6.416 | IWGSC_CSS_4AL_scaff_7173583 | 249 |
| lg4 | M147 | 6.614 | IWGSC_CSS_4AL_scaff_7173583 | 487 |
| lg4 | M112 | 7.386 | 4A | 204780670 |
| lg4 | M113 | 7.594 | 4A | 204780725 |
| lg4 | M94 | 8.665 | 4A | 201846318 |
| lg4 | M80 | 11.452 | 4A | 188725708 |
| lg4 | M81 | 12.621 | 4A | 189441819 |
| lg4 | M92 | 13.209 | 4A | 198579144 |
| lg4 | M73 | 13.829 | 4A | 182016081 |
| lg4 | M91 | 14.771 | 4A | 197972337 |
| lg4 | M89 | 17.454 | 4A | 194529034 |
| lg4 | M74 | 21.991 | 4A | 182852030 |
| lg4 | M1 | 22.425 | IWGSC_CSS_4AL_scaff_7013321 | 1681 |
| lg4 | M86 | 23.246 | 4A | 193227314 |
| lg4 | M90 | 23.688 | 4A | 195742783 |
| lg4 | M83 | 24.447 | 4A | 191468951 |
| lg4 | M85 | 24.609 | 4A | 191469147 |
| lg4 | M82 | 25.228 | 4A | 191468121 |
| lg4 | M87 | 25.587 | 4A | 194298023 |
| lg4 | M135 | 26.606 | IWGSC_CSS_4AL_scaff_7131501 | 2243 |
| lg4 | M134 | 26.645 | IWGSC_CSS_4AL_scaff_7131501 | 2204 |
| lg4 | M76 | 42.881 | 4A | 185438181 |
| lg4 | M88 | 44.411 | 4A | 194356752 |
| lg4 | M77 | 46.311 | 4A | 185620481 |
| lg4 | M72 | 50.532 | 4A | 176861005 |
| lg4 | M75 | 51.047 | 4A | 182861642 |
| lg4 | M78 | 51.714 | 4A | 188508745 |
| lg4 | M70 | 54.141 | 4A | 174839126 |
| lg4 | M4 | 64.229 | IWGSC_CSS_4AL_scaff_7101026 | 1228 |
| lg4 | M6 | 65.748 | IWGSC_CSS_4AL_scaff_7126694 | 4748 |
| lg4 | M148 | 67.481 | IWGSC_CSS_4AL_scaff_7063515 | 3615 |
| lg4 | M71 | 69.665 | 4A | 175034197 |
| lg4 | M144 | 77.753 | IWGSC_CSS_4AL_scaff_7154602 | 190 |
| lg4 | M137 | 79.773 | IWGSC_CSS_4AL_scaff_7156511 | 4145 |
| lg4 | M136 | 80.372 | IWGSC_CSS_4AL_scaff_7156511 | 4015 |
| lg4 | M69 | 86.461 | 4A | 174757752 |
| lg4 | M61 | 86.833 | 4A | 173447665 |
| lg4 | M57 | 87.511 | 4A | 173271543 |
| lg4 | M58 | 87.71 | 4A | 173271587 |
| lg4 | M60 | 88.366 | 4A | 173281152 |
| lg4 | M59 | 88.412 | 4A | 173280889 |
| lg4 | M63 | 89.387 | 4A | 174043718 |
| lg4 | M66 | 90.07 | 4A | 174043915 |
| lg4 | M62 | 90.684 | 4A | 173745848 |
| lg4 | M55 | 95.244 | 4A | 171538218 |
| lg4 | M52 | 96.258 | 4A | 170211982 |
| lg4 | M54 | 96.617 | 4A | 171366869 |
| lg4 | M56 | 98.278 | 4A | 171887111 |
| lg4 | M53 | 99.667 | 4A | 170501326 |
| lg4 | M149 | 102.345 | IWGSC_CSS_4AL_scaff_7073818 | 7293 |
| lg4 | M41 | 103.937 | 4A | 169724415 |
| lg4 | M39 | 104.83 | 4A | 169421760 |
| lg4 | M38 | 104.895 | 4A | 169421472 |
| lg4 | M35 | 105.285 | 4A | 169371265 |
| lg4 | M36 | 105.326 | 4A | 169371297 |
| lg4 | M37 | 105.411 | 4A | 169371501 |
| lg4 | M44 | 105.808 | 4A | 169771465 |
| lg4 | M48 | 105.828 | 4A | 169771706 |
| lg4 | M47 | 105.829 | 4A | 169771671 |
| lg4 | M49 | 105.833 | 4A | 169771733 |
| lg4 | M46 | 105.886 | 4A | 169771551 |
| lg4 | M32 | 106.46 | 4A | 168801534 |
| lg4 | M31 | 106.664 | 4A | 168801520 |
| lg4 | M34 | 107.191 | 4A | 169346600 |
| lg4 | M43 | 108.103 | 4A | 169737356 |
| lg4 | M42 | 108.25 | 4A | 169737110 |
| lg4 | M139 | 108.664 | IWGSC_CSS_4AL_scaff_7172321 | 1653 |
| lg4 | M143 | 109.888 | IWGSC_CSS_4AL_scaff_7097386 | 5604 |
| lg4 | M141 | 113.046 | IWGSC_CSS_4AL_scaff_7063933 | 3279 |
| lg4 | M9 | 115.378 | IWGSC_CSS_4AL_scaff_7160339 | 1065 |
| lg4 | M10 | 115.654 | IWGSC_CSS_4AL_scaff_7160339 | 1193 |
| lg4 | M7 | 115.759 | IWGSC_CSS_4AL_scaff_7160339 | 995 |
| lg4 | M28 | 116.279 | 4A | 140855561 |
| lg4 | M26 | 117.266 | 4A | 18611845 |
| lg4 | M27 | 130.866 | 4A | 49781350 |
| lg4 | M142 | 132.253 | IWGSC_CSS_4AS_scaff_5948762 | 5323 |
| lg4 | M24 | 160.528 | 4A | 10588768 |
| lg4 | M25 | 160.936 | 4A | 10588827 |
| lg4 | M21 | 163.05 | 4A | 5621050 |
| lg4 | M140 | 164.237 | IWGSC_CSS_4AS_scaff_5986494 | 2236 |
| lg4 | M23 | 165.61 | 4A | 6707666 |
| lg4 | M13 | 168.642 | 4A | 1049852 |
| lg4 | M16 | 169.054 | 4A | 1050144 |
| lg4 | M17 | 170.337 | 4A | 1171830 |
| lg4 | M18 | 171.628 | 4A | 1851272 |
| lg4 | M5 | 178.767 | IWGSC_CSS_4AS_scaff_6013991 | 11967 |
| lg4 | M150 | 179.884 | IWGSC_CSS_4AS_scaff_5997092 | 3454 |
| lg4 | M151 | 179.889 | IWGSC_CSS_4AS_scaff_5997092 | 3483 |
| lg4 | M152 | 179.907 | IWGSC_CSS_4AS_scaff_5997092 | 3674 |
| lg4 | M19 | 180.659 | 4A | 3544901 |
| lg4 | M138 | 181.8 | IWGSC_CSS_4AS_scaff_5988083 | 1883 |
| lg4 | M3 | 206.662 | IWGSC_CSS_4AS_scaff_6010640 | 9786 |
| lg5 | bin49 | 0 | IWGSC_CSS_5AL_scaff_2780998 | 934 |
| lg5 | bin37 | 0.5 | IWGSC_CSS_5AS_scaff_1481114 | 1754 |
| lg5 | bin124 | 0.762 | 5A | 41800569 |
| lg5 | bin119 | 1.799 | 5A | 37051937 |
| lg5 | bin91 | 2.638 | IWGSC_CSS_5AS_scaff_1544586 | 7027 |
| lg5 | bin17 | 3.012 | IWGSC_CSS_5AS_scaff_1503891 | 15421 |
| lg5 | bin75 | 3.325 | IWGSC_CSS_5AS_scaff_1531328 | 1842 |
| lg5 | bin77 | 3.584 | IWGSC_CSS_5AS_scaff_1508641 | 8584 |
| lg5 | bin51 | 3.796 | IWGSC_CSS_5AS_scaff_1525686 | 1338 |
| lg5 | bin87 | 3.908 | IWGSC_CSS_5AS_scaff_1509967 | 16134 |
| lg5 | bin29 | 4.059 | IWGSC_CSS_5AS_scaff_1515022 | 9127 |
| lg5 | bin72 | 4.179 | IWGSC_CSS_5AS_scaff_1540674 | 5506 |
| lg5 | bin86 | 4.283 | IWGSC_CSS_5AS_scaff_1546094 | 3148 |
| lg5 | bin27 | 4.391 | IWGSC_CSS_5AS_scaff_1539133 | 4853 |
| lg5 | bin140 | 4.529 | 5A | 62794519 |
| lg5 | bin9 | 4.579 | IWGSC_CSS_5AS_scaff_1552135 | 1836 |
| lg5 | bin14 | 4.719 | IWGSC_CSS_5AS_scaff_1514499 | 4093 |
| lg5 | bin92 | 4.81 | IWGSC_CSS_5AS_scaff_1544586 | 7276 |
| lg5 | bin30 | 4.88 | IWGSC_CSS_5AS_scaff_1553153 | 2212 |
| lg5 | bin123 | 4.959 | 5A | 39582728 |
| lg5 | bin57 | 5.027 | IWGSC_CSS_5AS_scaff_1541843 | 8785 |
| lg5 | bin40 | 5.101 | IWGSC_CSS_5AS_scaff_1528973 | 5508 |
| lg5 | bin44 | 5.235 | IWGSC_CSS_5AS_scaff_1505346 | 4700 |
| lg5 | bin13 | 5.299 | IWGSC_CSS_5AS_scaff_1543559 | 2937 |
| lg5 | bin58 | 5.359 | IWGSC_CSS_5AS_scaff_1533520 | 4982 |
| lg5 | bin22 | 5.457 | IWGSC_CSS_5AS_scaff_1540200 | 3962 |
| lg5 | bin70 | 5.534 | IWGSC_CSS_5AS_scaff_1552662 | 3346 |
| lg5 | bin118 | 5.602 | 5A | 33419349 |
| lg5 | bin39 | 5.669 | IWGSC_CSS_5AS_scaff_1504022 | 14280 |
| lg5 | bin94 | 5.736 | IWGSC_CSS_5AS_scaff_1525301 | 9598 |
| lg5 | bin68 | 5.806 | IWGSC_CSS_5AS_scaff_1551865 | 22965 |
| lg5 | bin95 | 5.844 | IWGSC_CSS_5AS_scaff_1503213 | 1293 |
| lg5 | bin134 | 5.896 | 5A | 52497961 |
| lg5 | bin36 | 5.952 | IWGSC_CSS_5AS_scaff_1463904 | 1127 |
| lg5 | bin1 | 6.028 | IWGSC_CSS_5AS_scaff_1544786 | 1732 |
| lg5 | bin129 | 6.089 | 5A | 47515979 |
| lg5 | bin2 | 6.147 | IWGSC_CSS_5AS_scaff_1544786 | 1927 |
| lg5 | bin11 | 6.205 | IWGSC_CSS_5AS_scaff_1552277 | 2830 |
| lg5 | bin122 | 6.274 | 5A | 39582372 |
| lg5 | bin31 | 6.318 | IWGSC_CSS_5AS_scaff_1551917 | 8544 |
| lg5 | bin23 | 6.4 | IWGSC_CSS_5AS_scaff_1463811 | 3269 |
| lg5 | bin43 | 6.465 | IWGSC_CSS_5AS_scaff_1549142 | 5201 |
| lg5 | bin78 | 6.557 | IWGSC_CSS_5AS_scaff_1524030 | 1044 |
| lg5 | bin110 | 6.578 | 5A | 22806563 |
| lg5 | bin19 | 6.639 | IWGSC_CSS_5AS_scaff_1544115 | 3892 |
| lg5 | bin61 | 6.689 | IWGSC_CSS_5AS_scaff_1552952 | 1072 |
| lg5 | bin73 | 6.734 | IWGSC_CSS_5AS_scaff_1540674 | 5706 |
| lg5 | bin114 | 6.763 | 5A | 26340160 |
| lg5 | bin138 | 6.804 | 5A | 60582590 |
| lg5 | bin76 | 6.848 | IWGSC_CSS_5AS_scaff_1551746 | 2823 |
| lg5 | bin33 | 6.947 | IWGSC_CSS_5AS_scaff_1510347 | 3320 |
| lg5 | bin100 | 6.985 | 5A | 12302566 |
| lg5 | bin84 | 7.044 | IWGSC_CSS_5AS_scaff_1550077 | 5828 |
| lg5 | bin41 | 7.092 | IWGSC_CSS_5AS_scaff_1516325 | 1488 |
| lg5 | bin117 | 7.179 | 5A | 32764769 |
| lg5 | bin97 | 7.24 | 5A | 95646 |
| lg5 | bin38 | 7.295 | IWGSC_CSS_5AS_scaff_1548337 | 5508 |
| lg5 | bin20 | 7.34 | IWGSC_CSS_5AS_scaff_1530944 | 8188 |
| lg5 | bin15 | 7.393 | IWGSC_CSS_5AS_scaff_1535197 | 8053 |
| lg5 | bin8 | 7.434 | IWGSC_CSS_5AS_scaff_1551157 | 1233 |
| lg5 | bin111 | 7.466 | 5A | 23176692 |
| lg5 | bin79 | 7.494 | IWGSC_CSS_5AS_scaff_1535791 | 3209 |
| lg5 | bin69 | 7.531 | IWGSC_CSS_5AS_scaff_1501523 | 312 |
| lg5 | bin90 | 7.544 | IWGSC_CSS_5AS_scaff_1550633 | 2708 |
| lg5 | bin26 | 7.56 | IWGSC_CSS_5AS_scaff_1549124 | 3400 |
| lg5 | bin128 | 7.575 | 5A | 46266948 |
| lg5 | bin63 | 7.588 | IWGSC_CSS_5AS_scaff_1533293 | 2033 |
| lg5 | bin65 | 7.614 | IWGSC_CSS_5AS_scaff_1519319 | 8571 |
| lg5 | bin130 | 7.635 | 5A | 47653261 |
| lg5 | bin71 | 7.667 | IWGSC_CSS_5AS_scaff_1529571 | 5782 |
| lg5 | bin55 | 7.718 | IWGSC_CSS_5AS_scaff_1521491 | 6393 |
| lg5 | bin93 | 7.75 | IWGSC_CSS_5AS_scaff_1528605 | 12055 |
| lg5 | bin56 | 7.773 | IWGSC_CSS_5AS_scaff_1521928 | 1074 |
| lg5 | bin25 | 7.797 | IWGSC_CSS_5AS_scaff_1509086 | 9477 |
| lg5 | bin6 | 7.815 | IWGSC_CSS_5AS_scaff_1539868 | 668 |
| lg5 | bin7 | 7.85 | IWGSC_CSS_5AS_scaff_630562 | 1434 |
| lg5 | bin108 | 7.871 | 5A | 21176545 |
| lg5 | bin53 | 7.901 | IWGSC_CSS_5AS_scaff_1504149 | 11088 |
| lg5 | bin42 | 7.959 | IWGSC_CSS_5AS_scaff_1516325 | 1721 |
| lg5 | bin89 | 8.016 | IWGSC_CSS_5AL_scaff_2754229 | 1259 |
| lg5 | bin16 | 8.079 | IWGSC_CSS_5AS_scaff_1535769 | 2310 |
| lg5 | bin32 | 8.12 | IWGSC_CSS_5AS_scaff_1528939 | 9722 |
| lg5 | bin126 | 8.156 | 5A | 44023613 |
| lg5 | bin54 | 8.185 | IWGSC_CSS_5AS_scaff_1553610 | 8318 |
| lg5 | bin46 | 8.21 | IWGSC_CSS_5AS_scaff_1517413 | 9789 |
| lg5 | bin132 | 8.269 | 5A | 50531716 |
| lg5 | bin133 | 8.327 | 5A | 51141808 |
| lg5 | bin88 | 8.35 | IWGSC_CSS_5AS_scaff_1552638 | 2852 |
| lg5 | bin99 | 8.393 | 5A | 11407684 |
| lg5 | bin18 | 8.42 | IWGSC_CSS_5AS_scaff_1539745 | 1652 |
| lg5 | bin64 | 8.498 | IWGSC_CSS_5AS_scaff_1524559 | 2894 |
| lg5 | bin59 | 8.561 | IWGSC_CSS_5AS_scaff_1506472 | 2250 |
| lg5 | bin35 | 8.615 | IWGSC_CSS_5AS_scaff_1530022 | 6744 |
| lg5 | bin34 | 8.684 | IWGSC_CSS_5AS_scaff_1530290 | 5479 |
| lg5 | bin62 | 8.73 | IWGSC_CSS_5AS_scaff_1518657 | 2949 |
| lg5 | bin120 | 8.779 | 5A | 38392135 |
| lg5 | bin21 | 8.867 | IWGSC_CSS_5AS_scaff_1553326 | 727 |
| lg5 | bin109 | 8.916 | 5A | 22791351 |
| lg5 | bin127 | 9.026 | 5A | 44111068 |
| lg5 | bin5 | 9.081 | IWGSC_CSS_5AS_scaff_1546292 | 2192 |
| lg5 | bin82 | 9.121 | IWGSC_CSS_5AS_scaff_1520466 | 3721 |
| lg5 | bin67 | 9.17 | IWGSC_CSS_5AS_scaff_1549426 | 7540 |
| lg5 | bin12 | 9.246 | IWGSC_CSS_5AS_scaff_1523396 | 4547 |
| lg5 | bin96 | 9.335 | IWGSC_CSS_5AS_scaff_1530042 | 1287 |
| lg5 | bin139 | 9.432 | 5A | 62467506 |
| lg5 | bin47 | 9.53 | IWGSC_CSS_5AS_scaff_1535290 | 4837 |
| lg5 | bin98 | 9.611 | 5A | 1910000 |
| lg5 | bin113 | 9.69 | 5A | 25381104 |
| lg5 | bin106 | 9.77 | 5A | 19529614 |
| lg5 | bin102 | 9.813 | 5A | 16044429 |
| lg5 | bin80 | 9.925 | IWGSC_CSS_5AS_scaff_1507670 | 1775 |
| lg5 | bin45 | 10.012 | IWGSC_CSS_5AS_scaff_1510209 | 8234 |
| lg5 | bin60 | 10.079 | IWGSC_CSS_5AS_scaff_1545224 | 2368 |
| lg5 | bin131 | 10.225 | 5A | 48825330 |
| lg5 | bin125 | 10.448 | 5A | 43393283 |
| lg5 | bin4 | 10.68 | IWGSC_CSS_5AS_scaff_1520993 | 2854 |
| lg5 | bin48 | 10.805 | IWGSC_CSS_5AS_scaff_1503984 | 1902 |
| lg5 | bin107 | 11.027 | 5A | 19588818 |
| lg5 | bin137 | 11.348 | 5A | 58058919 |
| lg5 | bin85 | 11.794 | IWGSC_CSS_5AL_scaff_2738930 | 3851 |
| lg5 | bin121 | 11.999 | 5A | 38874119 |
| lg5 | bin135 | 12.371 | 5A | 54204561 |
| lg5 | bin104 | 12.855 | 5A | 17942642 |
| lg5 | bin83 | 13.156 | IWGSC_CSS_5AL_scaff_2751910 | 4690 |
| lg5 | bin101 | 13.36 | 5A | 14327891 |
| lg5 | bin136 | 13.732 | 5A | 54371771 |
| lg5 | bin81 | 14.188 | IWGSC_CSS_5AL_scaff_2797553 | 1707 |
| lg5 | bin103 | 14.313 | 5A | 17537649 |
| lg5 | bin3 | 14.44 | IWGSC_CSS_5AL_scaff_2736665 | 1992 |
| lg5 | bin115 | 14.625 | 5A | 29040889 |
| lg5 | bin52 | 14.818 | IWGSC_CSS_5AL_scaff_2792621 | 3003 |
| lg5 | bin105 | 15.452 | 5A | 18993731 |
| lg5 | bin24 | 15.868 | IWGSC_CSS_5AL_scaff_2747785 | 766 |
| lg5 | bin116 | 16.232 | 5A | 29732470 |
| lg5 | bin112 | 17.604 | 5A | 23545040 |
| lg6 | M30 | 0 | 6A | 176530501 |
| lg6 | M29 | 0.333 | 6A | 176239668 |
| lg6 | M26 | 1.154 | 6A | 171680988 |
| lg6 | M19 | 1.533 | 6A | 159005355 |
| lg6 | M13 | 1.779 | 6A | 153378970 |
| lg6 | M12 | 2.092 | 6A | 152285353 |
| lg6 | M23 | 2.409 | 6A | 166898467 |
| lg6 | M22 | 2.65 | 6A | 166898355 |
| lg6 | M14 | 2.821 | 6A | 154296170 |
| lg6 | M15 | 3.004 | 6A | 154974873 |
| lg6 | M24 | 3.227 | 6A | 170576943 |
| lg6 | M31 | 3.362 | 6A | 177673904 |
| lg6 | M28 | 3.504 | 6A | 171862067 |
| lg6 | M20 | 3.714 | 6A | 162342140 |
| lg6 | M17 | 4.137 | 6A | 158113997 |
| lg6 | M21 | 4.512 | 6A | 163951853 |
| lg6 | M16 | 4.695 | 6A | 157389242 |
| lg6 | M18 | 4.793 | 6A | 158492629 |
| lg6 | M90 | 5.387 | 6A | 187984889 |
| lg6 | M102 | 5.583 | 6A | 189707592 |
| lg6 | M101 | 5.657 | 6A | 189707533 |
| lg6 | M99 | 5.783 | 6A | 189707364 |
| lg6 | M100 | 5.87 | 6A | 189707409 |
| lg6 | M65 | 6.108 | 6A | 185898578 |
| lg6 | M62 | 6.202 | 6A | 185898290 |
| lg6 | M63 | 6.217 | 6A | 185898343 |
| lg6 | M25 | 6.356 | 6A | 171271449 |
| lg6 | M89 | 6.531 | 6A | 187984725 |
| lg6 | M91 | 6.622 | 6A | 187984925 |
| lg6 | M80 | 6.757 | 6A | 186900099 |
| lg6 | M104 | 7.002 | 6A | 189963040 |
| lg6 | M114 | 7.189 | 6A | 191164365 |
| lg6 | M50 | 7.352 | 6A | 183039707 |
| lg6 | M121 | 7.383 | 6A | 191493107 |
| lg6 | M118 | 7.563 | 6A | 191336145 |
| lg6 | M60 | 7.7 | 6A | 184784147 |
| lg6 | M41 | 7.84 | 6A | 182309643 |
| lg6 | M94 | 7.947 | 6A | 188384320 |
| lg6 | M110 | 8.039 | 6A | 190212261 |
| lg6 | M109 | 8.045 | 6A | 190212226 |
| lg6 | M59 | 8.126 | 6A | 184784108 |
| lg6 | M66 | 8.214 | 6A | 186007365 |
| lg6 | M85 | 8.238 | 6A | 187392773 |
| lg6 | M34 | 8.278 | 6A | 181845893 |
| lg6 | M96 | 8.38 | 6A | 188768522 |
| lg6 | M95 | 8.403 | 6A | 188768272 |
| lg6 | M161 | 8.464 | IWGSC_CSS_6AL_scaff_5741009 | 1012 |
| lg6 | M61 | 8.514 | 6A | 184784356 |
| lg6 | M67 | 8.62 | 6A | 186007512 |
| lg6 | M57 | 8.645 | 6A | 184332588 |
| lg6 | M72 | 8.699 | 6A | 186060662 |
| lg6 | M71 | 8.721 | 6A | 186060446 |
| lg6 | M69 | 8.739 | 6A | 186060379 |
| lg6 | M70 | 8.8 | 6A | 186060413 |
| lg6 | M32 | 8.884 | 6A | 180831719 |
| lg6 | M126 | 8.978 | 6A | 192416471 |
| lg6 | M125 | 9.04 | 6A | 192416201 |
| lg6 | M3 | 9.126 | IWGSC_CSS_6AL_scaff_5812633 | 286 |
| lg6 | M7 | 9.17 | IWGSC_CSS_6AL_scaff_5812633 | 373 |
| lg6 | M113 | 9.223 | 6A | 190757380 |
| lg6 | M119 | 9.263 | 6A | 191336605 |
| lg6 | M98 | 9.328 | 6A | 188788618 |
| lg6 | M45 | 9.432 | 6A | 182357523 |
| lg6 | M86 | 9.486 | 6A | 187526382 |
| lg6 | M54 | 9.51 | 6A | 183419838 |
| lg6 | M75 | 9.608 | 6A | 186599000 |
| lg6 | M78 | 9.629 | 6A | 186599048 |
| lg6 | M36 | 9.67 | 6A | 182047846 |
| lg6 | M35 | 9.689 | 6A | 182047818 |
| lg6 | M39 | 9.693 | 6A | 182047891 |
| lg6 | M47 | 9.791 | 6A | 182825806 |
| lg6 | M97 | 9.815 | 6A | 188788343 |
| lg6 | M40 | 9.897 | 6A | 182181060 |
| lg6 | M44 | 9.996 | 6A | 182357248 |
| lg6 | M79 | 10.066 | 6A | 186628982 |
| lg6 | M48 | 10.202 | 6A | 182830300 |
| lg6 | M53 | 10.282 | 6A | 183077273 |
| lg6 | M74 | 10.389 | 6A | 186598856 |
| lg6 | M10 | 10.476 | IWGSC_CSS_6AL_scaff_5819818 | 1395 |
| lg6 | M58 | 10.562 | 6A | 184505534 |
| lg6 | M81 | 10.704 | 6A | 187101791 |
| lg6 | M82 | 10.721 | 6A | 187101857 |
| lg6 | M83 | 10.824 | 6A | 187102010 |
| lg6 | M117 | 10.957 | 6A | 191258361 |
| lg6 | M116 | 11.065 | 6A | 191258157 |
| lg6 | M46 | 11.344 | 6A | 182531382 |
| lg6 | M156 | 11.468 | IWGSC_CSS_6AL_scaff_5828797 | 8980 |
| lg6 | M108 | 11.629 | 6A | 190191317 |
| lg6 | M112 | 11.803 | 6A | 190271317 |
| lg6 | M155 | 12.096 | IWGSC_CSS_6AL_scaff_5835385 | 3430 |
| lg6 | M56 | 12.327 | 6A | 183529675 |
| lg6 | M55 | 12.339 | 6A | 183529441 |
| lg6 | M92 | 12.456 | 6A | 188152536 |
| lg6 | M93 | 12.591 | 6A | 188152577 |
| lg6 | M107 | 12.825 | 6A | 190158352 |
| lg6 | M124 | 13.025 | 6A | 191535858 |
| lg6 | M105 | 13.171 | 6A | 190140798 |
| lg6 | M160 | 13.42 | IWGSC_CSS_6AL_scaff_5780494 | 531 |
| lg6 | M106 | 13.715 | 6A | 190140897 |
| lg6 | M33 | 14.419 | 6A | 181106480 |
| lg6 | M127 | 58.812 | 6A | 198997660 |
| lg6 | M158 | 62.228 | IWGSC_CSS_6AL_scaff_5827863 | 4493 |
| lg6 | M128 | 87.923 | 6A | 204746520 |
| lg6 | M148 | 88.746 | 6A | 206071981 |
| lg6 | M152 | 89.272 | 6A | 206887272 |
| lg6 | M151 | 89.757 | 6A | 206887100 |
| lg6 | M150 | 89.77 | 6A | 206887039 |
| lg6 | M140 | 90.06 | 6A | 205112428 |
| lg6 | M153 | 90.261 | 6A | 207853395 |
| lg6 | M145 | 90.622 | 6A | 205592951 |
| lg6 | M141 | 90.928 | 6A | 205277360 |
| lg6 | M143 | 90.936 | 6A | 205277627 |
| lg6 | M146 | 91.658 | 6A | 206017973 |
| lg6 | M138 | 91.854 | 6A | 204869513 |
| lg6 | M139 | 91.91 | 6A | 204869553 |
| lg6 | M134 | 92.11 | 6A | 204869300 |
| lg6 | M137 | 92.379 | 6A | 204869355 |
| lg6 | M144 | 92.769 | 6A | 205360268 |
| lg6 | M132 | 93.628 | 6A | 204844165 |
| lg6 | M131 | 93.798 | 6A | 204844132 |
| lg6 | M149 | 94.495 | 6A | 206396953 |
| lg6 | M157 | 95.023 | IWGSC_CSS_6AL_scaff_5797521 | 555 |
| lg6 | M130 | 95.962 | 6A | 204811855 |
| lg6 | M2 | 99.642 | IWGSC_CSS_6AL_scaff_5809741 | 3106 |
| lg6 | M1 | 101.488 | IWGSC_CSS_6AL_scaff_5809741 | 2882 |
| lg7 | bin10 | 0 | 7A | 4921256 |
| lg7 | bin12 | 1.124 | 7A | 28228368 |
| lg7 | bin42 | 3.604 | 7A | 48859608 |
| lg7 | bin32 | 4.777 | 7A | 43888741 |
| lg7 | bin15 | 5.393 | 7A | 30001350 |
| lg7 | bin13 | 5.676 | 7A | 28828677 |
| lg7 | bin31 | 6.146 | 7A | 43888720 |
| lg7 | bin19 | 6.338 | 7A | 36909071 |
| lg7 | bin43 | 6.73 | 7A | 48859815 |
| lg7 | bin33 | 6.916 | 7A | 44146599 |
| lg7 | bin37 | 7.102 | 7A | 46753952 |
| lg7 | bin53 | 7.267 | 7A | 56551070 |
| lg7 | bin27 | 7.452 | 7A | 43663744 |
| lg7 | bin23 | 7.598 | 7A | 42485364 |
| lg7 | bin34 | 7.67 | 7A | 44865749 |
| lg7 | bin26 | 7.831 | 7A | 43224219 |
| lg7 | bin50 | 7.951 | 7A | 54370262 |
| lg7 | bin46 | 8.087 | 7A | 49975731 |
| lg7 | bin29 | 8.218 | 7A | 43818920 |
| lg7 | bin14 | 8.274 | 7A | 29418894 |
| lg7 | bin49 | 8.428 | 7A | 54171229 |
| lg7 | bin35 | 8.462 | 7A | 45295798 |
| lg7 | bin30 | 8.539 | 7A | 43888508 |
| lg7 | bin28 | 8.661 | 7A | 43663934 |
| lg7 | bin51 | 8.742 | 7A | 54522367 |
| lg7 | bin44 | 8.792 | 7A | 49100428 |
| lg7 | bin65 | 8.856 | 7A | 67842716 |
| lg7 | bin48 | 8.899 | 7A | 53007328 |
| lg7 | bin47 | 8.949 | 7A | 51906197 |
| lg7 | bin38 | 9.022 | 7A | 46921998 |
| lg7 | bin41 | 9.104 | 7A | 48492972 |
| lg7 | bin18 | 9.18 | 7A | 35281256 |
| lg7 | bin36 | 9.23 | 7A | 45474991 |
| lg7 | bin45 | 9.293 | 7A | 49669756 |
| lg7 | bin22 | 9.403 | 7A | 41350013 |
| lg7 | bin17 | 9.467 | 7A | 35226569 |
| lg7 | bin52 | 9.571 | 7A | 56300393 |
| lg7 | bin25 | 9.679 | 7A | 42711306 |
| lg7 | bin40 | 9.868 | 7A | 47162227 |
| lg7 | bin24 | 10.116 | 7A | 42612762 |
| lg7 | bin39 | 10.353 | 7A | 47162000 |
| lg7 | bin16 | 10.61 | 7A | 31943554 |
| lg7 | bin64 | 10.763 | 7A | 67265313 |
| lg7 | bin66 | 11.041 | 7A | 68139450 |
| lg7 | bin21 | 11.216 | 7A | 40176276 |
| lg7 | bin57 | 11.341 | 7A | 59273075 |
| lg7 | bin74 | 11.866 | 7A | 74999462 |
| lg7 | bin61 | 12.12 | 7A | 64721155 |
| lg7 | bin105 | 12.258 | 7A | 101108578 |
| lg7 | bin97 | 12.348 | 7A | 96265385 |
| lg7 | bin113 | 12.535 | 7A | 109428935 |
| lg7 | bin114 | 12.786 | 7A | 109635319 |
| lg7 | bin112 | 13.011 | 7A | 108770649 |
| lg7 | bin115 | 13.388 | 7A | 109783853 |
| lg7 | bin94 | 13.607 | 7A | 94807798 |
| lg7 | bin128 | 14.009 | 7A | 125104557 |
| lg7 | bin11 | 15.226 | 7A | 11927810 |
| lg7 | bin98 | 15.499 | 7A | 96995242 |
| lg7 | bin111 | 15.84 | 7A | 107781208 |
| lg7 | bin92 | 15.992 | 7A | 93292299 |
| lg7 | bin70 | 16.192 | 7A | 73131388 |
| lg7 | bin71 | 16.398 | 7A | 73137949 |
| lg7 | bin116 | 16.527 | 7A | 110045646 |
| lg7 | bin76 | 16.624 | 7A | 75567099 |
| lg7 | bin58 | 16.722 | 7A | 60148482 |
| lg7 | bin104 | 16.946 | 7A | 100229399 |
| lg7 | bin60 | 17.051 | 7A | 61626364 |
| lg7 | bin95 | 17.178 | 7A | 95308547 |
| lg7 | bin73 | 17.268 | 7A | 73676004 |
| lg7 | bin96 | 17.331 | 7A | 95807129 |
| lg7 | bin62 | 17.368 | 7A | 65694106 |
| lg7 | bin75 | 17.415 | 7A | 75121177 |
| lg7 | bin69 | 17.457 | 7A | 71350348 |
| lg7 | bin107 | 17.529 | 7A | 102054521 |
| lg7 | bin117 | 17.548 | 7A | 114294124 |
| lg7 | bin93 | 17.64 | 7A | 93823672 |
| lg7 | bin99 | 17.681 | 7A | 97710763 |
| lg7 | bin103 | 17.751 | 7A | 100229128 |
| lg7 | bin56 | 17.866 | 7A | 57757722 |
| lg7 | bin101 | 17.986 | 7A | 98952403 |
| lg7 | bin86 | 18.05 | 7A | 86026767 |
| lg7 | bin88 | 18.119 | 7A | 87815030 |
| lg7 | bin89 | 18.22 | 7A | 88072135 |
| lg7 | bin90 | 18.277 | 7A | 88499518 |
| lg7 | bin20 | 18.323 | 7A | 38770372 |
| lg7 | bin67 | 18.416 | 7A | 69285772 |
| lg7 | bin85 | 18.494 | 7A | 84863578 |
| lg7 | bin106 | 18.587 | 7A | 102054059 |
| lg7 | bin100 | 18.707 | 7A | 98603091 |
| lg7 | bin83 | 18.805 | 7A | 82388311 |
| lg7 | bin91 | 19.046 | 7A | 90497498 |
| lg7 | bin72 | 19.264 | 7A | 73339269 |
| lg7 | bin108 | 19.729 | 7A | 106594938 |
| lg7 | bin84 | 19.874 | 7A | 82562834 |
| lg7 | bin110 | 20.2 | 7A | 107698357 |
| lg7 | bin87 | 20.542 | 7A | 87043122 |
| lg7 | bin79 | 20.899 | 7A | 80733079 |
| lg7 | bin109 | 21.019 | 7A | 107530164 |
| lg7 | bin81 | 21.074 | 7A | 81854070 |
| lg7 | bin118 | 21.285 | 7A | 114663070 |
| lg7 | bin80 | 21.429 | 7A | 80795816 |
| lg7 | bin54 | 21.669 | 7A | 56827407 |
| lg7 | bin82 | 22.069 | 7A | 82035053 |
| lg7 | bin63 | 22.471 | 7A | 66860178 |
| lg7 | bin78 | 22.609 | 7A | 78023504 |
| lg7 | bin126 | 22.943 | 7A | 122614270 |
| lg7 | bin59 | 23.315 | 7A | 61210492 |
| lg7 | bin55 | 23.57 | 7A | 56939508 |
| lg7 | bin77 | 23.88 | 7A | 75898691 |
| lg7 | bin123 | 24.026 | 7A | 119022922 |
| lg7 | bin68 | 24.346 | 7A | 70549688 |
| lg7 | bin102 | 25.281 | 7A | 99415699 |
| lg7 | bin119 | 31.917 | 7A | 117342866 |
| lg7 | bin135 | 32.381 | 7A | 136833761 |
| lg7 | bin136 | 32.602 | 7A | 136833957 |
| lg7 | bin127 | 33.018 | 7A | 122914789 |
| lg7 | bin132 | 33.214 | 7A | 128171776 |
| lg7 | bin131 | 33.488 | 7A | 127870219 |
| lg7 | bin129 | 33.838 | 7A | 126723319 |
| lg7 | bin124 | 34.182 | 7A | 119333752 |
| lg7 | bin134 | 34.376 | 7A | 135980406 |
| lg7 | bin121 | 34.547 | 7A | 118862504 |
| lg7 | bin125 | 34.805 | 7A | 119455228 |
| lg7 | bin120 | 35.046 | 7A | 118612198 |
| lg7 | bin140 | 35.422 | 7A | 143151228 |
| lg7 | bin122 | 35.84 | 7A | 118947928 |
| lg7 | bin133 | 36.362 | 7A | 133068551 |
| lg7 | bin141 | 37.116 | 7A | 144578168 |
| lg7 | bin130 | 37.739 | 7A | 127204732 |
| lg7 | bin137 | 38.271 | 7A | 138449804 |
| lg7 | bin139 | 39.066 | 7A | 142951942 |
| lg7 | bin138 | 42.918 | 7A | 141158229 |
| lg7 | bin142 | 44.562 | 7A | 147196456 |
| lg7 | bin144 | 46.455 | 7A | 150192381 |
| lg7 | bin146 | 47.708 | 7A | 151987227 |
| lg7 | bin147 | 50.049 | 7A | 152368960 |
| lg7 | bin149 | 50.833 | 7A | 154882095 |
| lg7 | bin148 | 52.342 | 7A | 154786631 |
| lg7 | bin145 | 54.247 | 7A | 150274967 |
| lg8 | bin132 | 0 | 1B | 289119545 |
| lg8 | bin135 | 4.805 | 1B | 290776708 |
| lg8 | bin133 | 6.06 | 1B | 289490182 |
| lg8 | bin138 | 13.594 | 1B | 291683080 |
| lg8 | bin141 | 14.864 | 1B | 292129678 |
| lg8 | bin142 | 15.292 | 1B | 292726890 |
| lg8 | bin136 | 15.706 | 1B | 290920701 |
| lg8 | scaf3 | 16.365 | IWGSC_CSS_1BL_scaff_3750518 | 555 |
| lg8 | bin134 | 16.828 | 1B | 290448674 |
| lg8 | bin140 | 17.157 | 1B | 291901421 |
| lg8 | bin137 | 17.551 | 1B | 291038394 |
| lg8 | bin139 | 18.481 | 1B | 291858563 |
| lg8 | bin146 | 19.291 | 1B | 294831201 |
| lg8 | bin145 | 22.701 | 1B | 294761299 |
| lg8 | bin144 | 23.669 | 1B | 294611335 |
| lg8 | bin143 | 29.455 | 1B | 293598756 |
| lg8 | bin122 | 36.662 | 1B | 284605502 |
| lg8 | bin125 | 38.493 | 1B | 285544448 |
| lg8 | bin129 | 39.22 | 1B | 285791365 |
| lg8 | bin131 | 39.514 | 1B | 286762390 |
| lg8 | bin123 | 39.991 | 1B | 285298527 |
| lg8 | bin128 | 40.613 | 1B | 285791302 |
| lg8 | bin127 | 40.921 | 1B | 285744578 |
| lg8 | bin130 | 41.532 | 1B | 286121310 |
| lg8 | bin124 | 42.28 | 1B | 285521450 |
| lg8 | bin120 | 44.206 | 1B | 283994899 |
| lg8 | bin119 | 44.569 | 1B | 283994622 |
| lg8 | bin126 | 45.409 | 1B | 285714236 |
| lg8 | bin121 | 46.934 | 1B | 284230982 |
| lg8 | scaf2 | 52.795 | IWGSC_CSS_1BL_scaff_3918029 | 8152 |
| lg8 | bin118 | 53.795 | 1B | 280941347 |
| lg8 | bin115 | 54.823 | 1B | 280037083 |
| lg8 | bin111 | 56.177 | 1B | 277408094 |
| lg8 | bin114 | 56.924 | 1B | 279727318 |
| lg8 | bin110 | 57.673 | 1B | 277407892 |
| lg8 | bin117 | 58.149 | 1B | 280555571 |
| lg8 | bin112 | 58.616 | 1B | 279520201 |
| lg8 | bin116 | 58.928 | 1B | 280104182 |
| lg8 | bin106 | 59.309 | 1B | 276105818 |
| lg8 | scaf13 | 59.837 | IWGSC_CSS_1BL_scaff_3854674 | 797 |
| lg8 | bin113 | 60.549 | 1B | 279707059 |
| lg8 | bin104 | 69.203 | 1B | 274365542 |
| lg8 | scaf22 | 70.507 | IWGSC_CSS_1BL_scaff_3915358 | 1300 |
| lg8 | scaf21 | 70.514 | IWGSC_CSS_1BL_scaff_3915358 | 1277 |
| lg8 | scaf23 | 70.911 | IWGSC_CSS_1BL_scaff_3915358 | 1432 |
| lg8 | bin109 | 71.446 | 1B | 276790576 |
| lg8 | bin108 | 72.345 | 1B | 276645185 |
| lg8 | bin105 | 73.277 | 1B | 274471263 |
| lg8 | bin95 | 74.086 | 1B | 272199798 |
| lg8 | bin99 | 74.3 | 1B | 272779141 |
| lg8 | bin97 | 74.537 | 1B | 272311854 |
| lg8 | bin100 | 74.918 | 1B | 273043936 |
| lg8 | bin96 | 75.581 | 1B | 272287186 |
| lg8 | bin103 | 75.793 | 1B | 274187067 |
| lg8 | bin98 | 76.141 | 1B | 272405299 |
| lg8 | bin102 | 76.665 | 1B | 273886341 |
| lg8 | bin101 | 78.113 | 1B | 273520098 |
| lg8 | bin94 | 79.53 | 1B | 270404928 |
| lg8 | bin87 | 80.482 | 1B | 264071946 |
| lg8 | bin89 | 81.466 | 1B | 264748322 |
| lg8 | scaf20 | 82.137 | IWGSC_CSS_1BL_scaff_3898264 | 1531 |
| lg8 | scaf5 | 82.636 | IWGSC_CSS_1BL_scaff_3892496 | 4541 |
| lg8 | bin84 | 82.865 | 1B | 263728772 |
| lg8 | bin90 | 83.224 | 1B | 266778195 |
| lg8 | bin91 | 83.758 | 1B | 268467261 |
| lg8 | bin68 | 85.545 | 1B | 254842300 |
| lg8 | scaf12 | 86.662 | IWGSC_CSS_1BL_scaff_3818917 | 1080 |
| lg8 | bin85 | 87.044 | 1B | 263997068 |
| lg8 | bin86 | 87.186 | 1B | 263997277 |
| lg8 | bin93 | 87.863 | 1B | 270061679 |
| lg8 | bin92 | 88.037 | 1B | 269070234 |
| lg8 | bin88 | 88.158 | 1B | 264188995 |
| lg8 | scaf8 | 88.387 | IWGSC_CSS_1BL_scaff_3880795 | 485 |
| lg8 | bin83 | 88.519 | 1B | 262788231 |
| lg8 | bin79 | 88.663 | 1B | 259549967 |
| lg8 | bin74 | 88.949 | 1B | 257774220 |
| lg8 | bin82 | 89.02 | 1B | 262289173 |
| lg8 | bin81 | 89.267 | 1B | 260133932 |
| lg8 | bin77 | 89.396 | 1B | 258867544 |
| lg8 | scaf11 | 89.716 | IWGSC_CSS_1BL_scaff_3818917 | 883 |
| lg8 | bin80 | 89.964 | 1B | 259672034 |
| lg8 | bin78 | 90.113 | 1B | 259064732 |
| lg8 | scaf15 | 90.501 | IWGSC_CSS_1BL_scaff_3916799 | 10485 |
| lg8 | bin55 | 91.044 | 1B | 251438247 |
| lg8 | bin53 | 91.781 | 1B | 251313904 |
| lg8 | bin70 | 92.112 | 1B | 256032314 |
| lg8 | bin61 | 92.438 | 1B | 252843545 |
| lg8 | bin62 | 92.664 | 1B | 253181309 |
| lg8 | bin52 | 92.859 | 1B | 251313798 |
| lg8 | bin63 | 93.046 | 1B | 253181510 |
| lg8 | bin60 | 93.283 | 1B | 252539422 |
| lg8 | bin69 | 93.437 | 1B | 256007070 |
| lg8 | bin58 | 93.539 | 1B | 252323947 |
| lg8 | bin72 | 93.686 | 1B | 256369129 |
| lg8 | bin54 | 93.874 | 1B | 251329610 |
| lg8 | bin56 | 93.984 | 1B | 251888408 |
| lg8 | bin59 | 94.08 | 1B | 252366638 |
| lg8 | bin66 | 94.216 | 1B | 253859790 |
| lg8 | bin73 | 94.473 | 1B | 256796789 |
| lg8 | bin65 | 94.588 | 1B | 253859596 |
| lg8 | bin64 | 95.136 | 1B | 253583273 |
| lg8 | bin57 | 95.692 | 1B | 252186487 |
| lg8 | bin51 | 96.238 | 1B | 251001568 |
| lg8 | bin67 | 96.464 | 1B | 253970152 |
| lg8 | bin45 | 97.24 | 1B | 246653439 |
| lg8 | bin71 | 97.919 | 1B | 256294666 |
| lg8 | bin76 | 98.474 | 1B | 257939847 |
| lg8 | bin75 | 98.726 | 1B | 257939672 |
| lg8 | bin49 | 99.007 | 1B | 249013229 |
| lg8 | bin36 | 99.301 | 1B | 243013009 |
| lg8 | bin43 | 99.624 | 1B | 245045919 |
| lg8 | scaf16 | 99.836 | IWGSC_CSS_1BL_scaff_3877443 | 1695 |
| lg8 | scaf18 | 99.886 | IWGSC_CSS_1BL_scaff_3877443 | 1867 |
| lg8 | scaf17 | 99.956 | IWGSC_CSS_1BL_scaff_3877443 | 1755 |
| lg8 | bin33 | 100.116 | 1B | 241714106 |
| lg8 | bin46 | 100.186 | 1B | 246737364 |
| lg8 | bin40 | 100.278 | 1B | 243419896 |
| lg8 | bin37 | 100.339 | 1B | 243092767 |
| lg8 | scaf27 | 100.451 | IWGSC_CSS_1BL_scaff_3919698 | 16023 |
| lg8 | scaf9 | 100.525 | IWGSC_CSS_1BL_scaff_3895936 | 3030 |
| lg8 | bin38 | 100.594 | 1B | 243206324 |
| lg8 | bin50 | 100.798 | 1B | 249515329 |
| lg8 | bin39 | 100.902 | 1B | 243273775 |
| lg8 | bin41 | 101.108 | 1B | 243980855 |
| lg8 | bin48 | 101.203 | 1B | 248722577 |
| lg8 | scaf10 | 101.357 | IWGSC_CSS_1BL_scaff_3895936 | 3170 |
| lg8 | bin35 | 101.663 | 1B | 242980810 |
| lg8 | bin47 | 101.916 | 1B | 246912128 |
| lg8 | bin44 | 102.325 | 1B | 246251520 |
| lg8 | bin11 | 103.025 | 1B | 221404561 |
| lg8 | bin34 | 103.2 | 1B | 242693381 |
| lg8 | bin9 | 103.393 | 1B | 221395453 |
| lg8 | bin10 | 103.576 | 1B | 221398603 |
| lg8 | bin42 | 104.09 | 1B | 244079951 |
| lg8 | bin22 | 104.763 | 1B | 229627683 |
| lg8 | bin15 | 105.095 | 1B | 226950822 |
| lg8 | bin30 | 105.467 | 1B | 239956893 |
| lg8 | bin5 | 106.287 | 1B | 217197726 |
| lg8 | scaf24 | 107.262 | IWGSC_CSS_1BL_scaff_3861376 | 4278 |
| lg8 | bin32 | 108.72 | 1B | 240944934 |
| lg8 | scaf4 | 110.354 | IWGSC_CSS_1BL_scaff_3879088 | 479 |
| lg8 | bin28 | 111.572 | 1B | 235331031 |
| lg8 | bin14 | 112.536 | 1B | 226696688 |
| lg8 | bin24 | 113.382 | 1B | 231044649 |
| lg8 | bin18 | 113.869 | 1B | 228092679 |
| lg8 | bin8 | 114.372 | 1B | 220630876 |
| lg8 | bin17 | 114.577 | 1B | 227953847 |
| lg8 | bin6 | 114.71 | 1B | 217698626 |
| lg8 | bin16 | 114.767 | 1B | 227514543 |
| lg8 | bin13 | 114.928 | 1B | 226665005 |
| lg8 | bin29 | 115.121 | 1B | 239592252 |
| lg8 | bin31 | 115.238 | 1B | 240472198 |
| lg8 | bin27 | 115.454 | 1B | 233923514 |
| lg8 | bin21 | 115.769 | 1B | 228196373 |
| lg8 | bin19 | 116.027 | 1B | 228170949 |
| lg8 | bin12 | 116.374 | 1B | 222901509 |
| lg8 | bin20 | 116.612 | 1B | 228171286 |
| lg8 | scaf1 | 116.959 | IWGSC_CSS_1BL_scaff_3802970 | 1890 |
| lg8 | bin7 | 117.419 | 1B | 219422513 |
| lg8 | bin25 | 117.866 | 1B | 233046021 |
| lg8 | bin26 | 118.063 | 1B | 233046204 |
| lg8 | bin4 | 118.542 | 1B | 215601277 |
| lg8 | bin2 | 119.484 | 1B | 213800208 |
| lg8 | bin1 | 119.896 | 1B | 211838136 |
| lg8 | bin3 | 120.181 | 1B | 214877585 |
| lg9 | M1 | 0 | IWGSC_CSS_2BL_scaff_8085593 | 2916 |
| lg9 | M14 | 4.06 | IWGSC_CSS_2BL_scaff_7932724 | 4388 |
| lg9 | M15 | 4.98 | IWGSC_CSS_2BL_scaff_7932724 | 4528 |
| lg9 | M63 | 10.55 | 2B | 341876172 |
| lg9 | M58 | 13.17 | 2B | 341482421 |
| lg9 | M62 | 14.93 | 2B | 341842566 |
| lg9 | M61 | 14.93 | 2B | 341842297 |
| lg9 | M32 | 20.45 | 2B | 338280524 |
| lg9 | M33 | 20.45 | 2B | 338280614 |
| lg9 | M10 | 24.88 | IWGSC_CSS_2BL_scaff_7980703 | 2060 |
| lg9 | M11 | 24.88 | IWGSC_CSS_2BL_scaff_7980703 | 2091 |
| lg9 | M22 | 26.61 | IWGSC_CSS_2BL_scaff_8089744 | 32736 |
| lg9 | M60 | 29.05 | 2B | 341599738 |
| lg9 | M50 | 31.05 | 2B | 341261020 |
| lg9 | M52 | 31.85 | 2B | 341261505 |
| lg9 | M9 | 33.31 | IWGSC_CSS_2BL_scaff_7980703 | 1911 |
| lg9 | M48 | 35.24 | 2B | 341260346 |
| lg9 | M49 | 35.56 | 2B | 341260562 |
| lg9 | M55 | 37.13 | 2B | 341261869 |
| lg9 | M54 | 37.5 | 2B | 341261731 |
| lg9 | M64 | 39.13 | 2B | 342044681 |
| lg9 | M65 | 39.13 | 2B | 342044907 |
| lg9 | M42 | 40.8 | 2B | 339993375 |
| lg9 | M43 | 40.8 | 2B | 339993434 |
| lg9 | M38 | 41.65 | 2B | 339617507 |
| lg9 | M39 | 41.65 | 2B | 339617795 |
| lg9 | M31 | 42.67 | 2B | 338127535 |
| lg9 | M41 | 44.89 | 2B | 339632341 |
| lg9 | M67 | 48.23 | 2B | 342194083 |
| lg9 | M66 | 48.23 | 2B | 342193821 |
| lg9 | M47 | 51.26 | 2B | 340843352 |
| lg9 | M35 | 55.23 | 2B | 339409901 |
| lg9 | M34 | 55.23 | 2B | 339409863 |
| lg9 | M36 | 55.23 | 2B | 339410031 |
| lg9 | M45 | 60.5 | 2B | 340184955 |
| lg9 | M46 | 61.2 | 2B | 340185153 |
| lg9 | M27 | 65.47 | 2B | 337543922 |
| lg9 | M25 | 65.78 | 2B | 337543733 |
| lg10 | bin99 | 0 | 3B | 191813864 |
| lg10 | bin166 | 5.55 | 3B | 214602830 |
| lg10 | bin205 | 10.41 | 3B | 232005869 |
| lg10 | bin161 | 15.72 | 3B | 212926970 |
| lg10 | bin73 | 20.35 | 3B | 184036130 |
| lg10 | bin69 | 24.8 | 3B | 182644935 |
| lg10 | bin70 | 29.01 | 3B | 182669009 |
| lg10 | bin114 | 33.5 | 3B | 196380293 |
| lg10 | bin187 | 38.27 | 3B | 226835845 |
| lg10 | bin192 | 40.82 | 3B | 227518354 |
| lg10 | bin141 | 44.93 | 3B | 205256714 |
| lg10 | bin164 | 49.33 | 3B | 213054738 |
| lg10 | bin109 | 53.44 | 3B | 195483947 |
| lg10 | bin196 | 57.13 | 3B | 230316604 |
| lg10 | bin95 | 60.76 | 3B | 190124973 |
| lg10 | bin183 | 63.81 | 3B | 219578829 |
| lg10 | bin91 | 68.18 | 3B | 189250584 |
| lg10 | bin87 | 71.67 | 3B | 186974048 |
| lg10 | bin181 | 74.8 | 3B | 218378068 |
| lg10 | bin115 | 77.37 | 3B | 196959183 |
| lg10 | bin148 | 80.58 | 3B | 208202171 |
| lg10 | bin134 | 83.54 | 3B | 200508040 |
| lg10 | bin126 | 86.63 | 3B | 198991093 |
| lg10 | bin78 | 89.53 | 3B | 184951295 |
| lg10 | bin86 | 93.07 | 3B | 186205080 |
| lg10 | bin116 | 95.73 | 3B | 197028560 |
| lg10 | bin178 | 99.85 | 3B | 218071752 |
| lg10 | bin152 | 101.61 | 3B | 209530932 |
| lg10 | bin167 | 104.13 | 3B | 214675218 |
| lg10 | bin105 | 107.02 | 3B | 193441176 |
| lg10 | bin159 | 109.27 | 3B | 212923788 |
| lg10 | bin218 | 112.43 | 3B | 236021441 |
| lg10 | bin132 | 115.64 | 3B | 200354648 |
| lg10 | bin129 | 117.68 | 3B | 200183993 |
| lg10 | bin247 | 120.74 | 3B | 293625597 |
| lg10 | bin199 | 124.09 | 3B | 230779440 |
| lg10 | bin90 | 126.5 | 3B | 189217693 |
| lg10 | bin80 | 129.32 | 3B | 185414919 |
| lg10 | bin172 | 132.16 | 3B | 216396050 |
| lg10 | bin108 | 134.98 | 3B | 194557729 |
| lg10 | bin85 | 137.8 | 3B | 186202874 |
| lg10 | bin59 | 140.52 | 3B | 65824762 |
| lg10 | bin136 | 142.82 | 3B | 202315188 |
| lg10 | bin137 | 145.21 | 3B | 203713334 |
| lg10 | bin228 | 148.4 | 3B | 241293395 |
| lg10 | bin155 | 151.2 | 3B | 211349994 |
| lg10 | bin133 | 153.76 | 3B | 200493099 |
| lg10 | bin216 | 155.3 | 3B | 235506677 |
| lg10 | bin77 | 158.36 | 3B | 184186703 |
| lg10 | bin131 | 161.48 | 3B | 200351373 |
| lg10 | bin157 | 163.69 | 3B | 211776388 |
| lg10 | bin175 | 166.27 | 3B | 217192563 |
| lg10 | bin201 | 168.77 | 3B | 231046485 |
| lg10 | bin185 | 171.28 | 3B | 219905838 |
| lg10 | bin214 | 173.08 | 3B | 235255855 |
| lg10 | bin102 | 175.77 | 3B | 191901106 |
| lg10 | bin106 | 178.34 | 3B | 194264173 |
| lg10 | bin110 | 180.39 | 3B | 195618337 |
| lg10 | bin169 | 181.6 | 3B | 215644144 |
| lg10 | bin118 | 183.53 | 3B | 197162194 |
| lg10 | bin151 | 185.31 | 3B | 209478614 |
| lg10 | bin171 | 187.09 | 3B | 215940907 |
| lg10 | bin235 | 189.91 | 3B | 244319504 |
| lg10 | bin121 | 192.64 | 3B | 198368959 |
| lg10 | bin182 | 194.26 | 3B | 218850186 |
| lg10 | bin202 | 195.9 | 3B | 231141675 |
| lg10 | bin156 | 197.98 | 3B | 211550970 |
| lg10 | bin211 | 199.85 | 3B | 234305273 |
| lg10 | bin71 | 201.56 | 3B | 183249385 |
| lg10 | bin206 | 203.12 | 3B | 232136534 |
| lg10 | bin92 | 204.57 | 3B | 189706717 |
| lg10 | bin66 | 206.62 | 3B | 181753866 |
| lg10 | bin194 | 208.52 | 3B | 228097944 |
| lg10 | bin130 | 209.94 | 3B | 200208840 |
| lg10 | bin245 | 210.79 | 3B | 258346874 |
| lg10 | bin168 | 212.06 | 3B | 215368045 |
| lg10 | bin68 | 212.82 | 3B | 182377161 |
| lg10 | bin142 | 214.17 | 3B | 205679713 |
| lg10 | bin127 | 214.84 | 3B | 199959805 |
| lg10 | bin123 | 215.34 | 3B | 198845681 |
| lg10 | bin84 | 215.9 | 3B | 185979635 |
| lg10 | bin104 | 216.55 | 3B | 192325915 |
| lg10 | bin124 | 217.87 | 3B | 198850596 |
| lg10 | bin101 | 218.95 | 3B | 191883998 |
| lg10 | bin128 | 219.99 | 3B | 200128187 |
| lg10 | bin111 | 221.57 | 3B | 195971135 |
| lg10 | bin82 | 222.69 | 3B | 185741759 |
| lg10 | bin143 | 223.54 | 3B | 206912492 |
| lg10 | bin88 | 224.96 | 3B | 187248255 |
| lg10 | bin190 | 225.97 | 3B | 227083587 |
| lg10 | bin170 | 226.47 | 3B | 215880184 |
| lg10 | bin112 | 227.77 | 3B | 196000170 |
| lg10 | bin204 | 228.75 | 3B | 231742100 |
| lg10 | bin67 | 230.01 | 3B | 182065915 |
| lg10 | bin145 | 231.27 | 3B | 207229496 |
| lg10 | bin117 | 231.86 | 3B | 197032288 |
| lg10 | bin122 | 233.04 | 3B | 198475570 |
| lg10 | bin200 | 233.54 | 3B | 230958086 |
| lg10 | scaff0 | 234.2 | IWGSC_CSS_3B_scaff_10762116 | 882 |
| lg10 | bin177 | 234.61 | 3B | 217625090 |
| lg10 | bin147 | 235.01 | 3B | 207835032 |
| lg10 | bin203 | 235.81 | 3B | 231144488 |
| lg10 | bin188 | 236.82 | 3B | 227052328 |
| lg10 | bin154 | 237.32 | 3B | 211321578 |
| lg10 | bin100 | 238.24 | 3B | 191866192 |
| lg10 | bin195 | 239.48 | 3B | 230217206 |
| lg10 | scaff1 | 241.31 | IWGSC_CSS_3B_scaff_10762116 | 1052 |
| lg10 | bin207 | 242.3 | 3B | 232242868 |
| lg10 | bin120 | 243.63 | 3B | 198221579 |
| lg10 | bin215 | 245.2 | 3B | 235488804 |
| lg10 | bin184 | 246.43 | 3B | 219816751 |
| lg10 | bin93 | 247.83 | 3B | 189731703 |
| lg10 | bin176 | 249.06 | 3B | 217527826 |
| lg10 | bin125 | 250.49 | 3B | 198917567 |
| lg10 | bin180 | 251.42 | 3B | 218287189 |
| lg10 | bin186 | 252.97 | 3B | 220199364 |
| lg10 | bin153 | 253.78 | 3B | 209541366 |
| lg10 | bin144 | 254.54 | 3B | 207215070 |
| lg10 | bin149 | 255.53 | 3B | 209237966 |
| lg10 | bin213 | 256.67 | 3B | 235131571 |
| lg10 | bin58 | 258.08 | 3B | 65756964 |
| lg10 | bin135 | 259.67 | 3B | 200901996 |
| lg10 | bin226 | 261.48 | 3B | 240940432 |
| lg10 | bin191 | 263.6 | 3B | 227363030 |
| lg10 | bin212 | 265.63 | 3B | 234305762 |
| lg10 | bin89 | 266.9 | 3B | 188311599 |
| lg10 | bin198 | 269.19 | 3B | 230620157 |
| lg10 | bin146 | 271.37 | 3B | 207559122 |
| lg10 | bin225 | 272.91 | 3B | 240436841 |
| lg10 | bin119 | 275.31 | 3B | 197539357 |
| lg10 | bin173 | 277.25 | 3B | 216945161 |
| lg10 | bin160 | 278.95 | 3B | 212924753 |
| lg10 | bin223 | 280.12 | 3B | 236586719 |
| lg10 | bin174 | 282.31 | 3B | 216948161 |
| lg10 | bin208 | 285.51 | 3B | 233603613 |
| lg10 | bin139 | 287.74 | 3B | 204576308 |
| lg10 | bin244 | 290.31 | 3B | 258240008 |
| lg10 | bin189 | 292.44 | 3B | 227062545 |
| lg10 | bin79 | 294.97 | 3B | 185347896 |
| lg10 | bin94 | 297.04 | 3B | 190027210 |
| lg10 | bin210 | 299.39 | 3B | 234238626 |
| lg10 | bin113 | 302.16 | 3B | 196134117 |
| lg10 | bin81 | 304.92 | 3B | 185626908 |
| lg10 | bin103 | 307.39 | 3B | 192216902 |
| lg10 | bin221 | 310.1 | 3B | 236428254 |
| lg10 | bin217 | 310.78 | 3B | 235967182 |
| lg10 | bin97 | 311.75 | 3B | 190796320 |
| lg10 | bin193 | 314.21 | 3B | 227628868 |
| lg10 | bin165 | 316.92 | 3B | 214566299 |
| lg10 | bin150 | 318.65 | 3B | 209336572 |
| lg10 | bin72 | 321.48 | 3B | 183283176 |
| lg10 | bin233 | 324.68 | 3B | 244239012 |
| lg10 | bin45 | 325.96 | 3B | 31857137 |
| lg10 | bin234 | 326.62 | 3B | 244259171 |
| lg10 | bin243 | 327.82 | 3B | 249226315 |
| lg10 | bin224 | 331.52 | 3B | 240422565 |
| lg10 | bin163 | 334.85 | 3B | 213040138 |
| lg10 | bin231 | 339.04 | 3B | 244064901 |
| lg10 | bin232 | 339.9 | 3B | 244065026 |
| lg10 | bin239 | 342.1 | 3B | 245278285 |
| lg10 | bin98 | 344.38 | 3B | 190803132 |
| lg10 | bin238 | 346.03 | 3B | 244928614 |
| lg10 | bin51 | 347.76 | 3B | 33112130 |
| lg10 | bin96 | 350.12 | 3B | 190625570 |
| lg10 | bin158 | 352.7 | 3B | 211777478 |
| lg10 | bin138 | 356 | 3B | 204467543 |
| lg10 | bin237 | 359.83 | 3B | 244922415 |
| lg10 | bin230 | 361.91 | 3B | 244017384 |
| lg10 | bin197 | 365.84 | 3B | 230578644 |
| lg10 | bin242 | 369.28 | 3B | 249190118 |
| lg10 | bin162 | 373.34 | 3B | 212979851 |
| lg10 | bin222 | 376.17 | 3B | 236532271 |
| lg10 | bin140 | 379.57 | 3B | 204884898 |
| lg10 | bin240 | 383.56 | 3B | 248764628 |
| lg10 | bin50 | 385.63 | 3B | 32989191 |
| lg10 | bin219 | 388.35 | 3B | 236159311 |
| lg10 | bin241 | 391.73 | 3B | 248956141 |
| lg10 | bin227 | 394.11 | 3B | 241141887 |
| lg10 | bin46 | 396.41 | 3B | 31897672 |
| lg10 | bin47 | 399.89 | 3B | 32519857 |
| lg10 | bin236 | 403.49 | 3B | 244820463 |
| lg10 | bin179 | 408.21 | 3B | 218286933 |
| lg10 | bin220 | 412.71 | 3B | 236408531 |
| lg10 | bin229 | 416.14 | 3B | 241716855 |
| lg10 | bin107 | 421.14 | 3B | 194271587 |
| lg10 | bin48 | 425.84 | 3B | 32792934 |
| lg10 | bin209 | 430.82 | 3B | 233943310 |
| lg10 | bin76 | 434.97 | 3B | 184044818 |
| lg10 | bin74 | 435.24 | 3B | 184044644 |
| lg10 | bin75 | 436.57 | 3B | 184044673 |
| lg10 | bin49 | 444.11 | 3B | 32793100 |
| lg10 | bin255 | 454.55 | 3B | 469345491 |
| lg11 | M3 | 0 | 4B | 313641049 |
| lg11 | M1 | 10.131 | 4B | 309746438 |
| lg11 | M2 | 13.772 | 4B | 310454126 |
| lg12 | bin17 | 0 | 5B | 12498846 |
| lg12 | bin23 | 3.53 | 5B | 17175756 |
| lg12 | bin16 | 4.97 | 5B | 11575965 |
| lg12 | bin18 | 5.88 | 5B | 13516070 |
| lg12 | bin24 | 10.2 | 5B | 18121833 |
| lg12 | bin19 | 12.53 | 5B | 14093510 |
| lg12 | bin22 | 14.81 | 5B | 16439949 |
| lg12 | bin21 | 19.91 | 5B | 16436518 |
| lg12 | bin41 | 23.53 | 5B | 56275254 |
| lg12 | bin31 | 26.17 | 5B | 33051702 |
| lg12 | bin20 | 27.63 | 5B | 14373328 |
| lg12 | bin26 | 29 | 5B | 19685048 |
| lg12 | bin55 | 31.21 | 5B | 65290764 |
| lg12 | bin25 | 32.58 | 5B | 18545146 |
| lg12 | bin54 | 34.67 | 5B | 65290585 |
| lg12 | bin40 | 37.87 | 5B | 54810093 |
| lg12 | bin34 | 39.88 | 5B | 38675279 |
| lg12 | bin106 | 42.77 | 5B | 124476515 |
| lg12 | bin113 | 48.47 | 5B | 132025768 |
| lg12 | bin57 | 53.11 | 5B | 66553266 |
| lg12 | bin76 | 57.2 | 5B | 93985806 |
| lg12 | bin32 | 61.81 | 5B | 36373685 |
| lg12 | bin33 | 63.86 | 5B | 36890480 |
| lg12 | bin107 | 67.24 | 5B | 124912989 |
| lg12 | bin152 | 71.12 | 5B | 168687076 |
| lg12 | bin70 | 74.52 | 5B | 88150401 |
| lg12 | bin109 | 77.33 | 5B | 128520905 |
| lg12 | bin90 | 79.4 | 5B | 111744685 |
| lg12 | bin67 | 81.58 | 5B | 81881287 |
| lg12 | bin73 | 83.81 | 5B | 91590615 |
| lg12 | bin59 | 86.52 | 5B | 68437320 |
| lg12 | bin78 | 88.74 | 5B | 96470098 |
| lg12 | bin94 | 91.42 | 5B | 114527630 |
| lg12 | bin44 | 94.08 | 5B | 59234245 |
| lg12 | bin62 | 96.65 | 5B | 73492428 |
| lg12 | bin71 | 98.05 | 5B | 89967586 |
| lg12 | bin72 | 99.71 | 5B | 91144146 |
| lg12 | bin53 | 100.42 | 5B | 64369133 |
| lg12 | bin65 | 102.37 | 5B | 79678066 |
| lg12 | bin86 | 104.86 | 5B | 108884996 |
| lg12 | bin28 | 106.49 | 5B | 23391709 |
| lg12 | bin116 | 107.56 | 5B | 136529952 |
| lg12 | bin126 | 110.3 | 5B | 147889915 |
| lg12 | bin58 | 112.69 | 5B | 67771403 |
| lg12 | bin112 | 113.51 | 5B | 130370220 |
| lg12 | bin127 | 116.36 | 5B | 149057003 |
| lg12 | bin37 | 118.36 | 5B | 48174076 |
| lg12 | bin135 | 120.29 | 5B | 156023145 |
| lg12 | bin64 | 124.3 | 5B | 78631476 |
| lg12 | bin30 | 127.5 | 5B | 31383870 |
| lg12 | bin27 | 129.44 | 5B | 22501978 |
| lg12 | bin60 | 132.1 | 5B | 72393826 |
| lg12 | bin97 | 135.26 | 5B | 114855437 |
| lg12 | bin114 | 137.86 | 5B | 133529808 |
| lg12 | bin85 | 139.46 | 5B | 108028808 |
| lg12 | bin43 | 141.81 | 5B | 57908268 |
| lg12 | bin89 | 143.49 | 5B | 111628139 |
| lg12 | bin42 | 144.67 | 5B | 57856325 |
| lg12 | bin68 | 147.99 | 5B | 81930163 |
| lg12 | bin100 | 151.67 | 5B | 116765338 |
| lg12 | bin83 | 155.18 | 5B | 105606999 |
| lg12 | bin98 | 157.35 | 5B | 115297218 |
| lg12 | bin105 | 158.82 | 5B | 123656215 |
| lg12 | bin69 | 161.32 | 5B | 83001299 |
| lg12 | bin117 | 163.97 | 5B | 144292623 |
| lg12 | bin49 | 165.78 | 5B | 61030898 |
| lg12 | bin81 | 167.8 | 5B | 100645332 |
| lg12 | bin79 | 169.81 | 5B | 99714041 |
| lg12 | bin96 | 171.51 | 5B | 114789980 |
| lg12 | bin50 | 172.32 | 5B | 62066171 |
| lg12 | bin47 | 173.73 | 5B | 60442081 |
| lg12 | bin38 | 174.62 | 5B | 49535511 |
| lg12 | bin93 | 176.4 | 5B | 113913158 |
| lg12 | bin108 | 178.18 | 5B | 125468840 |
| lg12 | bin104 | 180.58 | 5B | 122818240 |
| lg12 | bin75 | 182.48 | 5B | 92426249 |
| lg12 | bin74 | 183.49 | 5B | 92426046 |
| lg12 | bin99 | 186.22 | 5B | 116699039 |
| lg12 | bin48 | 189.17 | 5B | 60756936 |
| lg12 | bin45 | 190.8 | 5B | 59883290 |
| lg12 | bin111 | 192.33 | 5B | 128721833 |
| lg12 | bin87 | 193.56 | 5B | 109455912 |
| lg12 | bin102 | 195.13 | 5B | 122415151 |
| lg12 | bin66 | 196.93 | 5B | 81169390 |
| lg12 | bin140 | 198.57 | 5B | 158178552 |
| lg12 | bin51 | 199.32 | 5B | 62746675 |
| lg12 | bin84 | 201.41 | 5B | 107162154 |
| lg12 | bin39 | 203.95 | 5B | 53876600 |
| lg12 | bin61 | 206.78 | 5B | 73431112 |
| lg12 | bin101 | 209.33 | 5B | 121650391 |
| lg12 | bin82 | 212.62 | 5B | 104513024 |
| lg12 | bin80 | 215.13 | 5B | 100411167 |
| lg12 | bin110 | 217.68 | 5B | 128721567 |
| lg12 | bin92 | 220.84 | 5B | 113291253 |
| lg12 | bin103 | 224.43 | 5B | 122648088 |
| lg12 | bin91 | 228.66 | 5B | 111912252 |
| lg12 | bin77 | 231.8 | 5B | 95321075 |
| lg12 | bin29 | 235.24 | 5B | 29256450 |
| lg12 | bin88 | 238 | 5B | 111509012 |
| lg12 | bin56 | 239.5 | 5B | 65561277 |
| lg12 | bin131 | 244.05 | 5B | 154968841 |
| lg12 | bin139 | 248.09 | 5B | 157927328 |
| lg12 | bin95 | 250.12 | 5B | 114714073 |
| lg12 | bin63 | 252.02 | 5B | 78558744 |
| lg12 | bin52 | 254.96 | 5B | 63570969 |
| lg12 | bin36 | 256.95 | 5B | 45494891 |
| lg12 | bin150 | 258.67 | 5B | 165761339 |
| lg12 | bin119 | 261.04 | 5B | 145808584 |
| lg12 | bin115 | 262.84 | 5B | 135901317 |
| lg12 | bin134 | 264.05 | 5B | 155578635 |
| lg12 | bin151 | 264.88 | 5B | 167612275 |
| lg12 | bin46 | 266.84 | 5B | 60014841 |
| lg12 | bin118 | 268.24 | 5B | 144686871 |
| lg12 | bin35 | 270.66 | 5B | 44726108 |
| lg12 | bin120 | 272.81 | 5B | 146130076 |
| lg12 | bin148 | 275.12 | 5B | 164787177 |
| lg12 | bin124 | 277.84 | 5B | 146752956 |
| lg12 | bin149 | 280.17 | 5B | 165761085 |
| lg12 | bin143 | 281.73 | 5B | 160150050 |
| lg12 | bin122 | 283.74 | 5B | 146510960 |
| lg12 | bin147 | 285.41 | 5B | 164346462 |
| lg12 | bin123 | 287.25 | 5B | 146511170 |
| lg12 | bin137 | 289.79 | 5B | 157256885 |
| lg12 | bin136 | 291.75 | 5B | 156220277 |
| lg12 | bin133 | 292.67 | 5B | 155401408 |
| lg12 | bin146 | 294.85 | 5B | 163443244 |
| lg12 | bin145 | 296.64 | 5B | 161438777 |
| lg12 | bin154 | 298.73 | 5B | 172177408 |
| lg12 | bin142 | 300.36 | 5B | 159610694 |
| lg12 | bin141 | 301.37 | 5B | 159610556 |
| lg12 | bin138 | 303.77 | 5B | 157790204 |
| lg12 | bin125 | 305.9 | 5B | 147780009 |
| lg12 | bin144 | 309.45 | 5B | 160234992 |
| lg12 | bin130 | 311.93 | 5B | 154415905 |
| lg12 | bin129 | 314.18 | 5B | 154060156 |
| lg12 | bin156 | 317.4 | 5B | 176434845 |
| lg12 | bin121 | 321.04 | 5B | 146414248 |
| lg12 | bin153 | 323.36 | 5B | 170496794 |
| lg12 | bin132 | 326.98 | 5B | 155401153 |
| lg12 | bin128 | 330.54 | 5B | 153467624 |
| lg12 | bin155 | 334.62 | 5B | 176189490 |
| lg12 | bin157 | 337.81 | 5B | 177134042 |
| lg12 | bin167 | 342.26 | 5B | 188246505 |
| lg12 | bin163 | 344.6 | 5B | 184864122 |
| lg12 | bin158 | 347.69 | 5B | 179386957 |
| lg12 | bin169 | 349.1 | 5B | 190438507 |
| lg12 | bin166 | 350.79 | 5B | 187895630 |
| lg12 | bin159 | 353.19 | 5B | 181646094 |
| lg12 | bin161 | 354.73 | 5B | 182575058 |
| lg12 | bin164 | 359.16 | 5B | 185401765 |
| lg12 | bin160 | 361.04 | 5B | 182078321 |
| lg12 | bin162 | 365.28 | 5B | 183189871 |
| lg12 | bin168 | 366.93 | 5B | 189918637 |
| lg12 | bin165 | 370.51 | 5B | 187832191 |
| lg13 | bin152 | 0 | 6B | 165998312 |
| lg13 | bin154 | 1.439 | 6B | 180379020 |
| lg13 | scaff28 | 2.409 | IWGSC_CSS_6BL_scaff_4258849 | 3477 |
| lg13 | scaff27 | 2.779 | IWGSC_CSS_6BL_scaff_4258849 | 3265 |
| lg13 | scaff8 | 3.703 | IWGSC_CSS_6BL_scaff_4288714 | 241 |
| lg13 | scaff21 | 4.711 | IWGSC_CSS_6BL_scaff_4327014 | 1674 |
| lg13 | bin153 | 5.452 | 6B | 179104570 |
| lg13 | scaff23 | 6.414 | IWGSC_CSS_6BL_scaff_4401011 | 1382 |
| lg13 | bin146 | 14.627 | 6B | 141127306 |
| lg13 | bin67 | 18.206 | 6B | 44271947 |
| lg13 | bin149 | 19.784 | 6B | 148786690 |
| lg13 | bin74 | 21.165 | 6B | 71401111 |
| lg13 | bin75 | 21.805 | 6B | 71401163 |
| lg13 | bin150 | 22.68 | 6B | 154952377 |
| lg13 | bin148 | 23.573 | 6B | 147122820 |
| lg13 | bin85 | 23.9 | 6B | 111825737 |
| lg13 | bin73 | 24.158 | 6B | 71400964 |
| lg13 | bin151 | 24.396 | 6B | 163649144 |
| lg13 | bin145 | 24.681 | 6B | 138245819 |
| lg13 | bin147 | 25.023 | 6B | 145002574 |
| lg13 | bin134 | 25.208 | 6B | 134149919 |
| lg13 | scaff29 | 25.323 | IWGSC_CSS_6BL_scaff_4368975 | 4867 |
| lg13 | bin83 | 25.484 | 6B | 107767456 |
| lg13 | bin86 | 25.624 | 6B | 112061347 |
| lg13 | bin72 | 25.89 | 6B | 71400426 |
| lg13 | bin80 | 26.07 | 6B | 74039511 |
| lg13 | bin87 | 26.165 | 6B | 113800935 |
| lg13 | bin125 | 26.277 | 6B | 131410258 |
| lg13 | bin78 | 26.423 | 6B | 73724169 |
| lg13 | bin76 | 26.591 | 6B | 72516654 |
| lg13 | bin79 | 26.716 | 6B | 73724199 |
| lg13 | bin77 | 26.802 | 6B | 73723984 |
| lg13 | bin82 | 26.974 | 6B | 104164986 |
| lg13 | bin68 | 27.204 | 6B | 49662464 |
| lg13 | bin71 | 27.5 | 6B | 69305867 |
| lg13 | bin69 | 27.865 | 6B | 64534895 |
| lg13 | bin70 | 28.154 | 6B | 64535057 |
| lg13 | bin108 | 28.519 | 6B | 121905128 |
| lg13 | bin81 | 29.671 | 6B | 80432374 |
| lg13 | bin88 | 33.779 | 6B | 115351642 |
| lg13 | bin89 | 34.945 | 6B | 115351674 |
| lg13 | bin107 | 35.607 | 6B | 120951534 |
| lg13 | bin127 | 36.223 | 6B | 132485715 |
| lg13 | bin128 | 36.899 | 6B | 132485912 |
| lg13 | bin133 | 37.396 | 6B | 133814310 |
| lg13 | bin112 | 37.687 | 6B | 123310831 |
| lg13 | bin132 | 37.761 | 6B | 133715659 |
| lg13 | bin106 | 37.989 | 6B | 119970883 |
| lg13 | bin129 | 38.209 | 6B | 132771463 |
| lg13 | bin131 | 38.441 | 6B | 133189793 |
| lg13 | bin144 | 38.646 | 6B | 138116434 |
| lg13 | bin84 | 38.965 | 6B | 110493093 |
| lg13 | bin117 | 39.288 | 6B | 126095955 |
| lg13 | bin139 | 39.472 | 6B | 136007305 |
| lg13 | bin114 | 39.619 | 6B | 124899908 |
| lg13 | bin120 | 39.76 | 6B | 128160331 |
| lg13 | bin113 | 39.965 | 6B | 124766199 |
| lg13 | bin123 | 40.093 | 6B | 129099366 |
| lg13 | bin121 | 40.259 | 6B | 128430173 |
| lg13 | bin90 | 40.372 | 6B | 115352360 |
| lg13 | bin122 | 40.534 | 6B | 128457977 |
| lg13 | bin99 | 40.729 | 6B | 117634715 |
| lg13 | scaff18 | 40.877 | IWGSC_CSS_6BS_scaff_2981232 | 1129 |
| lg13 | bin94 | 41.09 | 6B | 116100589 |
| lg13 | bin136 | 41.291 | 6B | 135402537 |
| lg13 | bin116 | 41.421 | 6B | 126038662 |
| lg13 | scaff17 | 41.605 | IWGSC_CSS_6BS_scaff_2969383 | 1662 |
| lg13 | scaff14 | 41.777 | IWGSC_CSS_6BS_scaff_2969383 | 1602 |
| lg13 | bin91 | 42.076 | 6B | 115352977 |
| lg13 | bin92 | 42.365 | 6B | 115513964 |
| lg13 | bin98 | 42.687 | 6B | 117358831 |
| lg13 | bin115 | 42.929 | 6B | 126012260 |
| lg13 | bin135 | 43.301 | 6B | 135033908 |
| lg13 | bin96 | 43.511 | 6B | 117056251 |
| lg13 | bin126 | 43.664 | 6B | 132404683 |
| lg13 | bin97 | 43.77 | 6B | 117056483 |
| lg13 | scaff6 | 43.916 | IWGSC_CSS_6BS_scaff_2946651 | 565 |
| lg13 | bin111 | 44.068 | 6B | 123112406 |
| lg13 | bin124 | 44.237 | 6B | 130572762 |
| lg13 | bin103 | 44.346 | 6B | 118306128 |
| lg13 | bin118 | 44.487 | 6B | 126692088 |
| lg13 | bin138 | 44.536 | 6B | 135642013 |
| lg13 | scaff38 | 44.696 | IWGSC_CSS_6BS_scaff_3045697 | 6895 |
| lg13 | scaff37 | 44.722 | IWGSC_CSS_6BS_scaff_3045697 | 6718 |
| lg13 | bin102 | 44.855 | 6B | 118305885 |
| lg13 | bin141 | 44.919 | 6B | 137700241 |
| lg13 | bin143 | 45.09 | 6B | 137967887 |
| lg13 | bin130 | 45.241 | 6B | 132860557 |
| lg13 | bin119 | 45.371 | 6B | 127539978 |
| lg13 | bin109 | 45.5 | 6B | 122019051 |
| lg13 | bin104 | 45.621 | 6B | 119843910 |
| lg13 | bin110 | 45.774 | 6B | 123066380 |
| lg13 | scaff4 | 45.909 | IWGSC_CSS_6BS_scaff_3021820 | 7613 |
| lg13 | bin93 | 46.045 | 6B | 116065427 |
| lg13 | bin95 | 46.233 | 6B | 116377341 |
| lg13 | bin101 | 46.428 | 6B | 117782911 |
| lg13 | scaff2 | 46.693 | IWGSC_CSS_6BS_scaff_1575979 | 925 |
| lg13 | bin105 | 46.832 | 6B | 119844297 |
| lg13 | scaff25 | 47.085 | IWGSC_CSS_6BS_scaff_3038471 | 499 |
| lg13 | scaff22 | 47.282 | IWGSC_CSS_6BS_scaff_3044964 | 1578 |
| lg13 | bin140 | 47.614 | 6B | 137497465 |
| lg13 | bin137 | 48.043 | 6B | 135514853 |
| lg13 | bin100 | 48.476 | 6B | 117782202 |
| lg13 | bin142 | 49.671 | 6B | 137724194 |
| lg13 | bin48 | 52.094 | 6B | 24544789 |
| lg13 | bin53 | 52.834 | 6B | 29351236 |
| lg13 | bin62 | 53.511 | 6B | 34008576 |
| lg13 | bin65 | 54.118 | 6B | 36005777 |
| lg13 | bin63 | 54.554 | 6B | 35305533 |
| lg13 | bin60 | 55.155 | 6B | 33345151 |
| lg13 | bin43 | 58.847 | 6B | 22885063 |
| lg13 | bin35 | 60.214 | 6B | 20431494 |
| lg13 | bin56 | 60.659 | 6B | 31784848 |
| lg13 | bin28 | 61.138 | 6B | 18176058 |
| lg13 | bin26 | 61.284 | 6B | 18175838 |
| lg13 | bin39 | 61.656 | 6B | 21424132 |
| lg13 | bin38 | 61.861 | 6B | 20700873 |
| lg13 | scaff0 | 62.077 | IWGSC_CSS_6BS_scaff_2949179 | 2492 |
| lg13 | bin27 | 62.275 | 6B | 18176054 |
| lg13 | bin40 | 62.427 | 6B | 21544580 |
| lg13 | bin44 | 62.624 | 6B | 22901026 |
| lg13 | bin34 | 62.722 | 6B | 20220524 |
| lg13 | bin54 | 62.838 | 6B | 30353824 |
| lg13 | bin23 | 62.922 | 6B | 16974222 |
| lg13 | bin30 | 63.033 | 6B | 19320068 |
| lg13 | bin46 | 63.179 | 6B | 23547221 |
| lg13 | bin32 | 63.271 | 6B | 20099550 |
| lg13 | bin36 | 63.417 | 6B | 20431699 |
| lg13 | bin58 | 63.512 | 6B | 32577846 |
| lg13 | bin25 | 63.602 | 6B | 17354114 |
| lg13 | bin61 | 63.719 | 6B | 33529302 |
| lg13 | bin50 | 63.764 | 6B | 25549974 |
| lg13 | bin45 | 63.96 | 6B | 23242621 |
| lg13 | bin47 | 63.999 | 6B | 23862085 |
| lg13 | bin57 | 64.107 | 6B | 32407827 |
| lg13 | bin29 | 64.177 | 6B | 19265202 |
| lg13 | bin66 | 64.371 | 6B | 36794515 |
| lg13 | bin31 | 64.445 | 6B | 19726967 |
| lg13 | bin20 | 64.581 | 6B | 11361472 |
| lg13 | bin41 | 64.765 | 6B | 22019714 |
| lg13 | bin37 | 64.982 | 6B | 20647863 |
| lg13 | bin19 | 65.128 | 6B | 11361022 |
| lg13 | bin52 | 65.283 | 6B | 28847305 |
| lg13 | bin49 | 65.423 | 6B | 24675330 |
| lg13 | bin33 | 65.611 | 6B | 20189502 |
| lg13 | bin51 | 65.717 | 6B | 25643371 |
| lg13 | bin24 | 65.95 | 6B | 17032613 |
| lg13 | bin55 | 66.183 | 6B | 31382586 |
| lg13 | bin59 | 66.448 | 6B | 32598232 |
| lg13 | bin22 | 66.827 | 6B | 16613121 |
| lg13 | bin21 | 67.277 | 6B | 15055415 |
| lg13 | bin64 | 67.531 | 6B | 35645468 |
| lg13 | scaff24 | 70.049 | IWGSC_CSS_6BS_scaff_3010756 | 3686 |
| lg13 | bin18 | 70.884 | 6B | 9024442 |
| lg13 | bin42 | 72.768 | 6B | 22019768 |
| lg13 | scaff3 | 74.426 | IWGSC_CSS_6BS_scaff_2973796 | 2772 |
| lg13 | bin17 | 75.162 | 6B | 7982636 |
| lg13 | bin16 | 79.553 | 6B | 7056863 |
| lg13 | bin15 | 84.856 | 6B | 6998013 |
| lg13 | bin14 | 88.712 | 6B | 5913831 |
| lg13 | scaff26 | 89.658 | IWGSC_CSS_6BS_scaff_1462959 | 1729 |
| lg13 | bin12 | 102.1 | 6B | 3691809 |
| lg13 | scaff9 | 102.672 | IWGSC_CSS_6BS_scaff_733818 | 375 |
| lg13 | bin13 | 103.408 | 6B | 4095321 |
| lg13 | bin9 | 104.46 | 6B | 3470733 |
| lg13 | bin8 | 105.295 | 6B | 3375800 |
| lg13 | bin11 | 105.725 | 6B | 3645353 |
| lg13 | bin10 | 106.728 | 6B | 3570144 |
| lg13 | bin5 | 113.652 | 6B | 1930822 |
| lg13 | bin3 | 114.681 | 6B | 873767 |
| lg13 | bin4 | 115.333 | 6B | 1122045 |
| lg13 | bin6 | 115.861 | 6B | 2241215 |
| lg13 | bin7 | 117.596 | 6B | 2689047 |
| lg13 | bin1 | 118.221 | 6B | 781715 |
| lg13 | bin2 | 118.737 | 6B | 781898 |
| lg13 | scaff34 | 119.703 | IWGSC_CSS_6BS_scaff_2960555 | 3064 |
| lg13 | scaff32 | 120.884 | IWGSC_CSS_6BS_scaff_2960555 | 2951 |
| lg13 | scaff33 | 122.824 | IWGSC_CSS_6BS_scaff_2960555 | 2969 |
| lg13 | scaff30 | 122.931 | IWGSC_CSS_6BS_scaff_2960555 | 2823 |
| lg13 | scaff31 | 123.485 | IWGSC_CSS_6BS_scaff_2960555 | 2862 |
| lg13 | scaff1 | 128.904 | IWGSC_CSS_6BS_scaff_2931263 | 9327 |
| lg14 | bin184 | 0 | 7B | 232503567 |
| lg14 | bin183 | 2.392 | 7B | 231641027 |
| lg14 | bin182 | 21 | 7B | 217651112 |
| lg14 | bin181 | 22.768 | 7B | 215566725 |
| lg14 | bin180 | 33.806 | 7B | 206943875 |
| lg14 | bin179 | 42.584 | 7B | 206191695 |
| lg14 | bin170 | 44.585 | 7B | 182506294 |
| lg14 | bin171 | 44.988 | 7B | 183289813 |
| lg14 | bin173 | 45.428 | 7B | 183695807 |
| lg14 | bin172 | 46.113 | 7B | 183616600 |
| lg14 | bin178 | 46.477 | 7B | 197313643 |
| lg14 | bin160 | 46.77 | 7B | 172853656 |
| lg14 | bin175 | 47.127 | 7B | 194986988 |
| lg14 | bin152 | 47.364 | 7B | 162935981 |
| lg14 | bin164 | 47.709 | 7B | 174987003 |
| lg14 | bin154 | 47.985 | 7B | 167324548 |
| lg14 | bin158 | 48.253 | 7B | 171890545 |
| lg14 | bin176 | 48.56 | 7B | 195856407 |
| lg14 | bin162 | 48.738 | 7B | 174713112 |
| lg14 | bin177 | 48.958 | 7B | 195864919 |
| lg14 | bin157 | 49.262 | 7B | 169725873 |
| lg14 | bin156 | 49.479 | 7B | 169725748 |
| lg14 | bin169 | 49.642 | 7B | 181900612 |
| lg14 | bin168 | 49.712 | 7B | 181862274 |
| lg14 | bin163 | 49.936 | 7B | 174713222 |
| lg14 | bin161 | 49.942 | 7B | 174712914 |
| lg14 | bin155 | 50.286 | 7B | 169119661 |
| lg14 | bin166 | 50.654 | 7B | 177131941 |
| lg14 | bin159 | 51 | 7B | 172846390 |
| lg14 | bin167 | 51.758 | 7B | 178849878 |
| lg14 | bin165 | 58.49 | 7B | 175817837 |
| lg14 | bin118 | 59.475 | 7B | 128651479 |
| lg14 | bin114 | 60.02 | 7B | 125177147 |
| lg14 | bin174 | 60.601 | 7B | 184165789 |
| lg14 | bin122 | 60.885 | 7B | 131767976 |
| lg14 | bin87 | 61.082 | 7B | 86613906 |
| lg14 | bin115 | 61.379 | 7B | 125340901 |
| lg14 | bin148 | 61.491 | 7B | 153582211 |
| lg14 | bin137 | 61.751 | 7B | 143565648 |
| lg14 | bin106 | 62.171 | 7B | 104403961 |
| lg14 | bin113 | 62.658 | 7B | 118707521 |
| lg14 | bin43 | 63.125 | 7B | 37054872 |
| lg14 | bin142 | 63.423 | 7B | 147756621 |
| lg14 | bin136 | 63.963 | 7B | 143360008 |
| lg14 | bin67 | 64.18 | 7B | 61319672 |
| lg14 | bin71 | 64.457 | 7B | 67496838 |
| lg14 | bin70 | 64.622 | 7B | 63359718 |
| lg14 | bin77 | 64.841 | 7B | 74320551 |
| lg14 | bin100 | 64.977 | 7B | 94058384 |
| lg14 | bin101 | 65.208 | 7B | 95489467 |
| lg14 | bin51 | 65.405 | 7B | 45241998 |
| lg14 | bin88 | 65.542 | 7B | 87243111 |
| lg14 | bin62 | 65.598 | 7B | 54197861 |
| lg14 | bin91 | 65.718 | 7B | 87614668 |
| lg14 | bin85 | 65.781 | 7B | 85469656 |
| lg14 | bin57 | 65.836 | 7B | 51511807 |
| lg14 | bin109 | 65.924 | 7B | 108386628 |
| lg14 | bin82 | 65.996 | 7B | 80189174 |
| lg14 | bin108 | 66.083 | 7B | 106726805 |
| lg14 | bin97 | 66.179 | 7B | 92841097 |
| lg14 | bin74 | 66.25 | 7B | 72141544 |
| lg14 | bin92 | 66.302 | 7B | 87614900 |
| lg14 | bin90 | 66.388 | 7B | 87563721 |
| lg14 | bin116 | 66.522 | 7B | 126481002 |
| lg14 | bin55 | 66.547 | 7B | 49159671 |
| lg14 | bin99 | 66.618 | 7B | 93569055 |
| lg14 | bin81 | 66.685 | 7B | 79884094 |
| lg14 | bin135 | 66.754 | 7B | 142382134 |
| lg14 | bin125 | 66.864 | 7B | 133186390 |
| lg14 | bin83 | 66.954 | 7B | 82349801 |
| lg14 | bin139 | 67.043 | 7B | 146527887 |
| lg14 | bin132 | 67.142 | 7B | 138477046 |
| lg14 | bin86 | 67.258 | 7B | 86230446 |
| lg14 | bin63 | 67.349 | 7B | 57401559 |
| lg14 | bin96 | 67.441 | 7B | 92531181 |
| lg14 | bin138 | 67.574 | 7B | 144110071 |
| lg14 | bin69 | 67.676 | 7B | 62681385 |
| lg14 | bin111 | 67.755 | 7B | 110470884 |
| lg14 | bin105 | 67.874 | 7B | 103412173 |
| lg14 | bin124 | 67.916 | 7B | 132575798 |
| lg14 | bin146 | 68 | 7B | 152548281 |
| lg14 | bin129 | 68.021 | 7B | 134412687 |
| lg14 | bin37 | 68.11 | 7B | 28876818 |
| lg14 | bin75 | 68.185 | 7B | 72585225 |
| lg14 | bin80 | 68.23 | 7B | 78753070 |
| lg14 | bin94 | 68.299 | 7B | 90787368 |
| lg14 | bin89 | 68.331 | 7B | 87523424 |
| lg14 | bin141 | 68.374 | 7B | 147664845 |
| lg14 | bin153 | 68.41 | 7B | 163175320 |
| lg14 | bin78 | 68.457 | 7B | 78053316 |
| lg14 | bin76 | 68.521 | 7B | 73435657 |
| lg14 | bin73 | 68.579 | 7B | 71760834 |
| lg14 | bin102 | 68.624 | 7B | 96040138 |
| lg14 | bin130 | 68.655 | 7B | 134508212 |
| lg14 | bin59 | 68.733 | 7B | 52020202 |
| lg14 | bin112 | 68.794 | 7B | 111490734 |
| lg14 | bin52 | 68.839 | 7B | 45255692 |
| lg14 | bin104 | 68.851 | 7B | 102872557 |
| lg14 | bin140 | 68.88 | 7B | 146696752 |
| lg14 | bin144 | 68.946 | 7B | 150383116 |
| lg14 | bin117 | 68.989 | 7B | 127643719 |
| lg14 | bin149 | 69.092 | 7B | 156752593 |
| lg14 | bin98 | 69.158 | 7B | 93275682 |
| lg14 | bin61 | 69.231 | 7B | 53956298 |
| lg14 | bin143 | 69.374 | 7B | 149523572 |
| lg14 | bin84 | 69.496 | 7B | 83273464 |
| lg14 | bin103 | 69.55 | 7B | 97456547 |
| lg14 | bin110 | 69.626 | 7B | 109525891 |
| lg14 | bin60 | 69.834 | 7B | 53392101 |
| lg14 | bin133 | 69.892 | 7B | 142107899 |
| lg14 | bin127 | 69.98 | 7B | 133577142 |
| lg14 | bin121 | 70.059 | 7B | 131379935 |
| lg14 | bin120 | 70.162 | 7B | 130433993 |
| lg14 | bin93 | 70.21 | 7B | 88145661 |
| lg14 | bin126 | 70.293 | 7B | 133393399 |
| lg14 | bin119 | 70.358 | 7B | 128827667 |
| lg14 | bin145 | 70.436 | 7B | 150748247 |
| lg14 | bin53 | 70.594 | 7B | 46918491 |
| lg14 | bin79 | 70.683 | 7B | 78663886 |
| lg14 | bin65 | 70.792 | 7B | 60164860 |
| lg14 | bin66 | 70.84 | 7B | 60165092 |
| lg14 | bin45 | 70.941 | 7B | 37689492 |
| lg14 | bin68 | 71.05 | 7B | 61574158 |
| lg14 | bin54 | 71.129 | 7B | 48624254 |
| lg14 | bin147 | 71.278 | 7B | 153254395 |
| lg14 | bin95 | 71.333 | 7B | 91105924 |
| lg14 | bin64 | 71.473 | 7B | 58009112 |
| lg14 | bin150 | 71.628 | 7B | 156798665 |
| lg14 | bin48 | 71.773 | 7B | 40977938 |
| lg14 | bin128 | 71.939 | 7B | 133935480 |
| lg14 | bin107 | 72.251 | 7B | 105882145 |
| lg14 | bin44 | 72.345 | 7B | 37213638 |
| lg14 | bin58 | 72.534 | 7B | 51617917 |
| lg14 | bin50 | 72.66 | 7B | 45126784 |
| lg14 | bin131 | 72.86 | 7B | 137728672 |
| lg14 | bin46 | 73.103 | 7B | 37689599 |
| lg14 | bin56 | 73.496 | 7B | 51335832 |
| lg14 | bin123 | 73.809 | 7B | 132232691 |
| lg14 | bin72 | 74.335 | 7B | 70843865 |
| lg14 | bin134 | 79.427 | 7B | 142273904 |
| lg14 | bin49 | 80.615 | 7B | 42583631 |
| lg14 | bin151 | 81.363 | 7B | 161850402 |
| lg14 | bin39 | 81.522 | 7B | 32537182 |
| lg14 | bin42 | 82.229 | 7B | 35003927 |
| lg14 | bin47 | 82.705 | 7B | 39218622 |
| lg14 | bin41 | 83.098 | 7B | 33157413 |
| lg14 | bin38 | 83.832 | 7B | 30400924 |
| lg14 | bin40 | 85.796 | 7B | 32732135 |
| lg14 | bin20 | 91.207 | 7B | 11487516 |
| lg14 | bin36 | 93.141 | 7B | 27730190 |
| lg14 | bin13 | 93.698 | 7B | 5959782 |
| lg14 | bin32 | 94.513 | 7B | 20776680 |
| lg14 | bin23 | 94.824 | 7B | 16065492 |
| lg14 | bin22 | 95.001 | 7B | 14125796 |
| lg14 | bin34 | 95.209 | 7B | 23297686 |
| lg14 | bin25 | 95.448 | 7B | 17004422 |
| lg14 | bin30 | 95.695 | 7B | 19096692 |
| lg14 | bin16 | 96.124 | 7B | 9524990 |
| lg14 | bin14 | 97.267 | 7B | 6463267 |
| lg14 | bin17 | 97.656 | 7B | 9794617 |
| lg14 | bin35 | 97.884 | 7B | 27031805 |
| lg14 | bin19 | 98.17 | 7B | 9840804 |
| lg14 | bin21 | 98.397 | 7B | 13823658 |
| lg14 | bin24 | 98.682 | 7B | 16738514 |
| lg14 | bin31 | 98.93 | 7B | 19893549 |
| lg14 | bin18 | 99.127 | 7B | 9832608 |
| lg14 | bin28 | 99.337 | 7B | 18055006 |
| lg14 | bin33 | 99.654 | 7B | 21851043 |
| lg14 | bin15 | 100.132 | 7B | 8397295 |
| lg14 | bin26 | 100.578 | 7B | 17939053 |
| lg14 | bin27 | 100.912 | 7B | 17939249 |
| lg14 | bin29 | 101.992 | 7B | 18626885 |
| lg15 | M21 | 0 | 1D | 64156587 |
| lg15 | M19 | 3.11 | 1D | 56199257 |
| lg15 | M17 | 9 | 1D | 46148486 |
| lg15 | M7 | 14.66 | IWGSC_CSS_1DS_scaff_1881788 | 1148 |
| lg15 | M12 | 19.09 | IWGSC_CSS_1DS_scaff_1896364 | 794 |
| lg15 | M20 | 21.61 | 1D | 63279196 |
| lg15 | M2 | 23.84 | IWGSC_CSS_1DS_scaff_1897562 | 2079 |
| lg15 | M23 | 25.24 | 1D | 67772492 |
| lg15 | M18 | 26.31 | 1D | 51587410 |
| lg15 | M11 | 27.71 | IWGSC_CSS_1DS_scaff_1908462 | 3635 |
| lg15 | M8 | 30.06 | IWGSC_CSS_1DS_scaff_1896213 | 4544 |
| lg15 | M15 | 32.68 | 1D | 34006774 |
| lg15 | M24 | 36.28 | 1D | 73286109 |
| lg15 | M16 | 37.22 | 1D | 39297213 |
| lg15 | M6 | 40.5 | IWGSC_CSS_1DL_scaff_2254278 | 6494 |
| lg16 | M33 | 0 | 2D | 43902562 |
| lg16 | M38 | 3.202 | 2D | 61136477 |
| lg16 | M37 | 7.514 | 2D | 59752617 |
| lg16 | M32 | 11.624 | 2D | 41608714 |
| lg16 | M36 | 12.061 | 2D | 56382794 |
| lg16 | M34 | 13.095 | 2D | 44990863 |
| lg16 | M87 | 13.594 | IWGSC_CSS_2DS_scaff_5363553 | 1438 |
| lg16 | M41 | 14.155 | 2D | 67750398 |
| lg16 | M58 | 14.882 | 2D | 107436443 |
| lg16 | M63 | 15.888 | 2D | 117948428 |
| lg16 | M60 | 16.903 | 2D | 116677716 |
| lg16 | M61 | 17.073 | 2D | 117203998 |
| lg16 | M86 | 18.374 | IWGSC_CSS_2DS_scaff_5329660 | 2265 |
| lg16 | M57 | 19.043 | 2D | 102836751 |
| lg16 | M44 | 19.751 | 2D | 73096323 |
| lg16 | M66 | 20.056 | 2D | 126379909 |
| lg16 | M35 | 20.349 | 2D | 45310483 |
| lg16 | M59 | 20.754 | 2D | 108527839 |
| lg16 | M64 | 21.26 | 2D | 121909709 |
| lg16 | M52 | 21.485 | 2D | 87531611 |
| lg16 | M45 | 21.921 | 2D | 74286525 |
| lg16 | M53 | 22.202 | 2D | 90424633 |
| lg16 | M65 | 22.951 | 2D | 124234488 |
| lg16 | M54 | 23.121 | 2D | 95262156 |
| lg16 | M83 | 23.545 | IWGSC_CSS_2DL_scaff_9891765 | 1988 |
| lg16 | M49 | 24.5 | 2D | 85231960 |
| lg16 | M67 | 25.819 | 2D | 130317770 |
| lg16 | M39 | 27.833 | 2D | 63755606 |
| lg16 | M56 | 27.981 | 2D | 96660558 |
| lg16 | M47 | 28.543 | 2D | 80375192 |
| lg16 | M42 | 29.402 | 2D | 69591237 |
| lg16 | M40 | 31.854 | 2D | 65101369 |
| lg16 | M51 | 32.596 | 2D | 87103442 |
| lg16 | M43 | 34.417 | 2D | 72380314 |
| lg16 | M48 | 35.243 | 2D | 81175559 |
| lg16 | M28 | 40.231 | 2D | 25242890 |
| lg16 | M31 | 41.178 | 2D | 40959822 |
| lg16 | M29 | 41.512 | 2D | 28922915 |
| lg16 | M30 | 42.31 | 2D | 32903958 |
| lg16 | M55 | 43.331 | 2D | 96482893 |
| lg16 | M68 | 46.96 | 2D | 131272425 |
| lg16 | M46 | 47.722 | 2D | 76358162 |
| lg16 | M84 | 49.484 | IWGSC_CSS_2DL_scaff_9850560 | 117 |
| lg16 | M69 | 53.402 | 2D | 132294739 |
| lg16 | M70 | 56.95 | 2D | 136805221 |
| lg16 | M71 | 61.124 | 2D | 140258883 |
| lg16 | M74 | 64.007 | 2D | 142300676 |
| lg16 | M75 | 65.157 | 2D | 143901713 |
| lg16 | M76 | 65.281 | 2D | 143901888 |
| lg16 | M79 | 65.982 | 2D | 146627704 |
| lg16 | M80 | 66.185 | 2D | 146627895 |
| lg16 | M73 | 67.61 | 2D | 141642109 |
| lg16 | M72 | 68.46 | 2D | 140819189 |
| lg16 | M1 | 77.66 | 2D | 4622693 |
| lg16 | M27 | 78.546 | 2D | 8975038 |
| lg16 | M26 | 78.752 | 2D | 8975004 |
| lg16 | M85 | 82.068 | IWGSC_CSS_2DL_scaff_9908256 | 8989 |
| lg16 | M2 | 83.325 | 2D | 6762531 |
| lg16 | M18 | 83.901 | 2D | 7716107 |
| lg16 | M82 | 84.14 | 2D | 148111292 |
| lg16 | M23 | 84.391 | 2D | 8367060 |
| lg16 | M24 | 84.417 | 2D | 8367295 |
| lg16 | M22 | 84.927 | 2D | 8118168 |
| lg16 | M19 | 85.361 | 2D | 7716108 |
| lg16 | M5 | 85.715 | 2D | 6994408 |
| lg16 | M6 | 85.908 | 2D | 6994491 |
| lg16 | M7 | 85.949 | 2D | 6994526 |
| lg16 | M3 | 85.976 | 2D | 6994293 |
| lg16 | M25 | 86.148 | 2D | 8561931 |
| lg16 | M11 | 86.469 | 2D | 7072242 |
| lg16 | M12 | 86.58 | 2D | 7072455 |
| lg16 | M81 | 86.924 | 2D | 147027872 |
| lg16 | M8 | 87.723 | 2D | 7065673 |
| lg16 | M10 | 87.824 | 2D | 7065947 |
| lg16 | M17 | 88.715 | 2D | 7559359 |
| lg16 | M14 | 88.896 | 2D | 7559128 |
| lg16 | M16 | 89.065 | 2D | 7559302 |
| lg17 | M12 | 0 | 3D | 19155016 |
| lg17 | M39 | 5.66 | 3D | 74566413 |
| lg17 | M31 | 8.469 | 3D | 60024938 |
| lg17 | M33 | 8.996 | 3D | 62813601 |
| lg17 | M10 | 9.378 | 3D | 14579391 |
| lg17 | M26 | 9.929 | 3D | 50918642 |
| lg17 | M19 | 10.237 | 3D | 30304701 |
| lg17 | M37 | 10.429 | 3D | 69360556 |
| lg17 | M35 | 10.606 | 3D | 66006402 |
| lg17 | M28 | 10.81 | 3D | 53210735 |
| lg17 | M36 | 11.07 | 3D | 69058075 |
| lg17 | M11 | 11.168 | 3D | 16636770 |
| lg17 | M34 | 11.387 | 3D | 63541480 |
| lg17 | M42 | 11.541 | 3D | 78603167 |
| lg17 | M24 | 11.552 | 3D | 39321141 |
| lg17 | M21 | 11.6 | 3D | 32286195 |
| lg17 | M25 | 11.628 | 3D | 41499077 |
| lg17 | M20 | 11.784 | 3D | 30853620 |
| lg17 | M17 | 11.87 | 3D | 27528337 |
| lg17 | M23 | 12.026 | 3D | 36174421 |
| lg17 | M40 | 12.13 | 3D | 74600951 |
| lg17 | M27 | 12.411 | 3D | 51494991 |
| lg17 | M14 | 12.741 | 3D | 21722853 |
| lg17 | M30 | 13.087 | 3D | 59668462 |
| lg17 | M13 | 13.348 | 3D | 19656730 |
| lg17 | M38 | 13.568 | 3D | 70619427 |
| lg17 | M22 | 13.948 | 3D | 34020070 |
| lg17 | M15 | 15.903 | 3D | 23063889 |
| lg17 | M9 | 16.457 | 3D | 14239919 |
| lg17 | M16 | 17.061 | 3D | 26364520 |
| lg17 | M32 | 17.378 | 3D | 61133099 |
| lg17 | M5 | 24.256 | IWGSC_CSS_3DL_scaff_6894116 | 3738 |
| lg17 | M43 | 28.634 | 3D | 88254035 |
| lg17 | M18 | 31.077 | 3D | 27636626 |
| lg17 | M41 | 33.111 | 3D | 77951174 |
| lg17 | M45 | 51.179 | 3D | 94133337 |
| lg17 | M47 | 54.518 | 3D | 96012965 |
| lg17 | M44 | 56.643 | 3D | 93403783 |
| lg17 | M46 | 60.903 | 3D | 95390089 |
| lg17 | M49 | 73.803 | 3D | 101608048 |
| lg17 | M50 | 75.438 | 3D | 102964928 |
| lg17 | M48 | 76.626 | 3D | 97829076 |
| lg17 | M51 | 78.584 | 3D | 104976989 |
| lg17 | M52 | 84.334 | 3D | 107290608 |
| lg17 | M57 | 92.581 | IWGSC_CSS_3DL_scaff_6956041 | 2726 |
| lg17 | M53 | 111.33 | 3D | 112607720 |
| lg17 | M54 | 123.156 | 3D | 114653148 |
| lg18 | M8 | 0 | 4D | 116434711 |
| lg18 | M9 | 18.355 | 4D | 120084176 |
| lg19 | M2 | 0 | 5D | 18733588 |
| lg19 | M27 | 12.81 | 5D | 99784041 |
| lg19 | M12 | 18.06 | 5D | 38903770 |
| lg19 | M31 | 19.92 | IWGSC_CSS_5DS_scaff_2762168 | 1773 |
| lg19 | M34 | 23.32 | IWGSC_CSS_5DS_scaff_2743721 | 3428 |
| lg19 | M10 | 24.73 | 5D | 36068625 |
| lg19 | M26 | 25.95 | 5D | 98018784 |
| lg19 | M15 | 28.42 | 5D | 59112161 |
| lg19 | M35 | 30.87 | IWGSC_CSS_5DL_scaff_4510364 | 688 |
| lg19 | M1 | 32.92 | IWGSC_CSS_5DS_scaff_2772308 | 788 |
| lg19 | M11 | 34.16 | 5D | 38733746 |
| lg19 | M16 | 35.32 | 5D | 70034112 |
| lg19 | M5 | 35.65 | 5D | 25835299 |
| lg19 | M4 | 36.77 | 5D | 24238426 |
| lg19 | M9 | 37.64 | 5D | 34022989 |
| lg19 | M36 | 39.4 | IWGSC_CSS_5DS_scaff_2738369 | 791 |
| lg19 | M37 | 40.44 | IWGSC_CSS_5DS_scaff_2776154 | 2210 |
| lg19 | M13 | 42.14 | 5D | 55947011 |
| lg19 | M19 | 44.76 | 5D | 80132394 |
| lg19 | M38 | 47.96 | IWGSC_CSS_5DL_scaff_4562635 | 3521 |
| lg19 | M32 | 50.72 | IWGSC_CSS_5DL_scaff_4572240 | 5135 |
| lg19 | M3 | 53.41 | 5D | 20975356 |
| lg19 | M20 | 58.64 | 5D | 81321826 |
| lg19 | M6 | 61.77 | 5D | 28478197 |
| lg19 | M24 | 63.56 | 5D | 96676230 |
| lg19 | M17 | 65.92 | 5D | 72344639 |
| lg19 | M23 | 67.42 | 5D | 95350055 |
| lg19 | M22 | 68.72 | 5D | 84432985 |
| lg19 | M14 | 70.78 | 5D | 57571239 |
| lg19 | M25 | 75.25 | 5D | 96947276 |
| lg19 | M18 | 80.52 | 5D | 77106984 |
| lg19 | M21 | 82.96 | 5D | 82798052 |
| lg20 | M2 | 0 | 6D | 71917333 |
| lg20 | M4 | 2.644 | IWGSC_CSS_6DS_scaff_2072219 | 564 |
| lg21 | M19 | 0 | 7D | 94540107 |
| lg21 | M7 | 0.94 | IWGSC_CSS_7DS_scaff_3909854 | 6452 |
| lg21 | M23 | 1.184 | 7D | 111973331 |
| lg21 | M34 | 1.435 | 7D | 129076800 |
| lg21 | M45 | 2.578 | 7D | 157964270 |
| lg21 | M31 | 2.977 | 7D | 124597842 |
| lg21 | M28 | 3.761 | 7D | 118190440 |
| lg21 | M30 | 3.807 | 7D | 124083579 |
| lg21 | M32 | 4.019 | 7D | 128456073 |
| lg21 | M46 | 4.276 | 7D | 160703531 |
| lg21 | M38 | 5.468 | 7D | 136851985 |
| lg21 | M17 | 5.748 | 7D | 87376381 |
| lg21 | M42 | 6.031 | 7D | 149021281 |
| lg21 | M18 | 6.502 | 7D | 92560961 |
| lg21 | M1 | 6.615 | IWGSC_CSS_7DL_scaff_3323959 | 724 |
| lg21 | M37 | 6.713 | 7D | 136144865 |
| lg21 | M55 | 6.749 | 7D | 184432981 |
| lg21 | M27 | 6.807 | 7D | 117887473 |
| lg21 | M43 | 7.144 | 7D | 150417385 |
| lg21 | M33 | 7.223 | 7D | 128459823 |
| lg21 | M24 | 7.323 | 7D | 113057617 |
| lg21 | M11 | 7.42 | 7D | 53645452 |
| lg21 | M57 | 7.499 | 7D | 185915538 |
| lg21 | M35 | 7.603 | 7D | 132255927 |
| lg21 | M44 | 7.793 | 7D | 152365103 |
| lg21 | M25 | 7.997 | 7D | 116052300 |
| lg21 | M29 | 8.1 | 7D | 120022078 |
| lg21 | M13 | 8.209 | 7D | 67135047 |
| lg21 | M22 | 8.335 | 7D | 109144363 |
| lg21 | M21 | 8.608 | 7D | 108967591 |
| lg21 | M15 | 8.851 | 7D | 72880688 |
| lg21 | M39 | 9.083 | 7D | 139127325 |
| lg21 | M5 | 9.316 | IWGSC_CSS_7DL_scaff_3342159 | 4931 |
| lg21 | M53 | 9.531 | 7D | 174520690 |
| lg21 | M36 | 9.737 | 7D | 132596261 |
| lg21 | M54 | 10.149 | 7D | 181103489 |
| lg21 | M52 | 10.623 | 7D | 172003836 |
| lg21 | M69 | 11.765 | IWGSC_CSS_7DL_scaff_3392963 | 5745 |
| lg21 | M16 | 16.502 | 7D | 84514133 |
| lg21 | M4 | 17.498 | IWGSC_CSS_7DL_scaff_3395963 | 3343 |
| lg21 | M41 | 19.007 | 7D | 147707612 |
| lg21 | M14 | 19.829 | 7D | 70049232 |
| lg21 | M40 | 20.554 | 7D | 140615554 |
| lg21 | M47 | 21.769 | 7D | 169879703 |
| lg21 | M20 | 28.362 | 7D | 103969213 |
| lg21 | M12 | 30.577 | 7D | 57794242 |
| lg21 | M56 | 36.109 | 7D | 184969992 |
| lg21 | M68 | 37.488 | IWGSC_CSS_7DL_scaff_3316500 | 2790 |
| lg21 | M59 | 38.736 | 7D | 192699923 |
| lg21 | M58 | 39.238 | 7D | 187553253 |
| lg21 | M60 | 39.701 | 7D | 192753678 |
| lg21 | M61 | 40.222 | 7D | 193614633 |
| lg21 | M51 | 40.793 | 7D | 170450437 |
| lg21 | M48 | 41.051 | 7D | 170450237 |
| lg21 | M3 | 42.851 | IWGSC_CSS_7DL_scaff_3348774 | 517 |
| lg21 | M2 | 53.54 | IWGSC_CSS_7DL_scaff_3296517 | 6517 |
| lg21 | M67 | 54.288 | IWGSC_CSS_7DL_scaff_3317099 | 629 |
| lg21 | M63 | 54.993 | 7D | 202607025 |
| lg21 | M6 | 55.499 | IWGSC_CSS_7DL_scaff_3354666 | 776 |
| lg21 | M65 | 64.159 | 7D | 213092903 |
| lg21 | M64 | 68.76 | 7D | 212747809 |

Table S2 Genes located in the intervals of *Pis1*

| No. | start position | end position | BLAST matching accession No. | Annotation | Sequence identity (%) | *E* value |
| --- | --- | --- | --- | --- | --- | --- |
| 1 | 136830471 | 136831272 | KD550407 | N/A | 100 | 0 |
| 2 | 136833019 | 136834097 | AAT06527 | polyphenol oxidase [*T. aestivum*] | 100 | 0 |
| 3 | 136901712 | 136902385 | XP_008663459 | Probable purine permease 11 [*Zea mays*] | 71% | 5e-48 |
| 4 | 136914077 | 136914958 | XP_003576007 | N/A |  |  |
| 5 | 136925310 | 136930180 | EMT00889 | Lupus brain antigen 1-like protein [*Aegilops tauschii*] | 81% | 0 |
| 6 | 136934295 | 136935713 | ABF50675 | vacuolar ATPase subunit G [*Triticum aestivum*] | 99% | 1e-69 |
| 7 | 136939535 | 136940151 | XP_010236956 | 60 kDa jasmonate-induced protein-like [*Brachypodium distachyon*] | 68% | 2e-40 |
| 8 | 136965144 | 136966051 | EMT00293 | N/A |  |  |
| 9 | 136975967 | 136980867 | EMS59123 | putative polyamine oxidase 2 [*Triticum urartu*] | 100% | 0 |
| 10 | 136996439 | 136996945 | XP_010230178 | N/A |  |  |
| 11 | 137009261 | 137012184 | XP_010240336 | alpha-1,3/1,6-mannosyltransferase ALG2 [*Brachypodium distachyon*] | 97% | 0 |
| 12 | 137060004 | 137066286 | EMS53270 | Putative glucose-6-phosphate  1-epimerase [*Triticum urartu*] | 99% | 0 |
| 13 | 137066916 | 137070253 | EMS53269 | E3 ubiquitin ligase BIG BROTHER-related protein [*Triticum urartu*] | 96% | 0 |
| 14 | 137111448 | 137113227 | EMT24888 | E3 ubiquitin-protein ligase RNF14 [*Aegilops tauschii*] | 97% | 0 |
| 15 | 137149364 | 137154504 | EMT06562 | Disease resistance protein RGA2 [*Aegilops tauschii*] | 95% | 0 |
| 16 | 137172404 | 137172606 | XP_010239711 | N/A |  |  |
| 17 | 137175076 | 137178894 | XP_015692361 | altered inheritance rate of mitochondria protein 25 [*Oryza brachyantha*] | 91% | 3e-132 |
| 18 | 137191749 | 137192043 | A[BA97240](http://www.ncbi.nlm.nih.gov/protein/77554444?report=genbank&log$=protalign&blast_rank=2&RID=HDU9GKEP013) | transposon protein, putative, Mutator sub-class [*Oryza sativa Japonica Group*] | 62% | 3e-37 |
| 19 | 137194431 | 137196413 | EMS62021 | Anthocyanidin reductase [*Triticum urartu*] | 94% | 0 |
| 20 | 137226086 | 137230251 | AAS48876 | expansin EXPA7 [*Triticum aestivum*] | 99% | 0 |
| 21 | 137230939 | 137231469 | ACG30677 | histone H4 [*Zea mays*], | 83% | 6e-47 |
| 22 | 137247044 | 137248957 | EMT23289 | Lupus brain antigen 1-like protein [*Aegilops tauschii*] | 99% | 0 |
| 23 | 137262979 | 137276670 | EMT00889 | Lupus brain antigen 1-like protein [*Aegilops tauschii*] | 96% | 0 |
| 24 | 137317948 | 137318676 | [EMT21921](http://www.ncbi.nlm.nih.gov/protein/475591926?report=genbank&log$=protalign&blast_rank=1&RID=HEAA7C1H016) | N/A | 100% | 4e-50 |
| 25 | 137333645 | 137334476 | EMT21701 | N/A | 88% | 7e-38 |
| 26 | 137364070 | 137366051 | [EMS57067](http://www.ncbi.nlm.nih.gov/protein/474154141?report=genbank&log$=protalign&blast_rank=1&RID=HEC7TYD7013) | Adenosylhomocysteinase [*Triticum urartu*] | 93% | 0 |
| 27 | 137462135 | 137466398 | [EMS50970](http://www.ncbi.nlm.nih.gov/protein/473957469?report=genbank&log$=protalign&blast_rank=1&RID=HECE2TMM013) | Protein pelota [*Triticum urartu*] | 99% | 8e-161 |
| 28 | 137486971 | 137491091 | EMT19251 | Putative serine/  threonine-protein kinase receptor [*Aegilops tauschii*] | 94% | 0 |
| 29 | 137495058 | 137495909 | [CDM81269](http://www.ncbi.nlm.nih.gov/protein/669027720?report=genbank&log$=prottop&blast_rank=1&RID=HHNKPJPU015) | [unnamed protein product [*Triticum aestivum*]](http://blast.ncbi.nlm.nih.gov/Blast.cgi#alnHdr_669027720) | 77% | 2e-42 |
| 30 | 137508510 | 137514506 | [XP_003563214](http://www.ncbi.nlm.nih.gov/protein/357123026?report=genbank&log$=protalign&blast_rank=3&RID=HEHP5DEF013) | zinc finger matrin-type protein 2 [*Brachypodium distachyon*] | 95% | 1e-118 |
| 31 | 137529097 | 137531310 | CAA77236 | amylogenin [*Triticum aestivum*] | 100% | 0 |
| 32 | 137552816 | 137559309 | [EMT11270](http://www.ncbi.nlm.nih.gov/protein/475549533?report=genbank&log$=prottop&blast_rank=1&RID=HF1R9N2D015) | [Putative protein phosphatase](http://blast.ncbi.nlm.nih.gov/Blast.cgi" \l "alnHdr_475549533" \o "Go to alignment for Putative protein phosphatase 2C 45 [Aegilops tauschii])  [2C 45 [](http://blast.ncbi.nlm.nih.gov/Blast.cgi" \l "alnHdr_475549533" \o "Go to alignment for Putative protein phosphatase 2C 45 [Aegilops tauschii])*[Aegilops tauschii](http://blast.ncbi.nlm.nih.gov/Blast.cgi" \l "alnHdr_475549533" \o "Go to alignment for Putative protein phosphatase 2C 45 [Aegilops tauschii])*[]](http://blast.ncbi.nlm.nih.gov/Blast.cgi" \l "alnHdr_475549533" \o "Go to alignment for Putative protein phosphatase 2C 45 [Aegilops tauschii]) | 90% | 4e-157 |
| 33 | 137594293 | 137595552 | [AAM90696](http://www.ncbi.nlm.nih.gov/protein/22086626?report=genbank&log$=protalign&blast_rank=7&RID=HG5VZ4FU01R) | S-locus receptor-like kinase RLK11 [*Oryza sativa*] | 81% | 4e-140 |
| 34 | 137618648 | 137619244 | [EMT02575](http://www.ncbi.nlm.nih.gov/protein/475475406?report=genbank&log$=protalign&blast_rank=1&RID=HG6CMH6101R) | N/A |  |  |
| 35 | 137635810 | 137644474 | [EMT24018](http://www.ncbi.nlm.nih.gov/protein/475598720?report=genbank&log$=protalign&blast_rank=1&RID=HG708PXS01R) | Vacuolar proton translocating ATPase 100 kDa subunit [*Aegilops tauschii*] | 98% | 0 |
| 36 | 137655461 | 137656429 | [NP_001150854](http://www.ncbi.nlm.nih.gov/protein/226499738?report=genbank&log$=protalign&blast_rank=8&RID=HG7DA5AS014) | sulfate transporter [*Zea mays*] | 87% | 4e-68 |
| 37 | 137662097 | 137662216 | [YP_008239143](http://www.ncbi.nlm.nih.gov/protein/525778556?report=genbank&log$=prottop&blast_rank=1&RID=HGE96K0W01R) | ribosomal protein L32 (chloroplast) [*Triticum monococcum*] | 100% | 1e-19 |
| 38 | 137681344 | 137684889 | [EMT32642](http://www.ncbi.nlm.nih.gov/protein/475624494?report=genbank&log$=prottop&blast_rank=1&RID=HGFUFUXN01R) | Protein IQ-DOMAIN 32 [*Aegilops tauschii*] | 97% | 0 |
| 39 | 137686199 | 137690721 | [EMT32642](http://www.ncbi.nlm.nih.gov/protein/475624494?report=genbank&log$=prottop&blast_rank=1&RID=HGFUFUXN01R) | [Protein IQ-DOMAIN 32 [*Aegilops tauschii*]](http://blast.ncbi.nlm.nih.gov/Blast.cgi#alnHdr_475624494) | 99% | 0 |
| 40 | 137700889 | 137701342 | [EMT01687](http://www.ncbi.nlm.nih.gov/protein/475450916?report=genbank&log$=prottop&blast_rank=1&RID=HGGUCSB9015) | [Tropinone reductase-like protein [*Aegilops tauschii*]](http://blast.ncbi.nlm.nih.gov/Blast.cgi#alnHdr_475450916) | 91% | 3e-39 |
| 41 | 137721835 | 137727231 | [EMS60314](http://www.ncbi.nlm.nih.gov/protein/474253768?report=genbank&log$=prottop&blast_rank=1&RID=HGHZHU4M01R) | [Putative glucose-6-phosphate 1-epimerase [*Triticum urartu*]](http://blast.ncbi.nlm.nih.gov/Blast.cgi#alnHdr_474253768) | 94% | 0 |
| 42 | 137751982 | 137752611 | [ADG43138](http://www.ncbi.nlm.nih.gov/protein/295844282?report=genbank&log$=prottop&blast_rank=1&RID=HGU33A0M01R) | [auxin response factor 4 [*Zea mays*]](http://blast.ncbi.nlm.nih.gov/Blast.cgi#alnHdr_295844282) | 76% | 4e-13 |
| 43 | 137763450 | 137774947 | [BAK04770](http://www.ncbi.nlm.nih.gov/protein/326529647?report=genbank&log$=prottop&blast_rank=1&RID=HGUAKRKC015) | [Serine/threonine-protein kinase sepA [*Triticum urartu*]](http://blast.ncbi.nlm.nih.gov/Blast.cgi#alnHdr_474350547) | 98% | 0 |
| 44 | 137789607 | 137790891 | [EMS61905](http://www.ncbi.nlm.nih.gov/protein/474311274?report=genbank&log$=prottop&blast_rank=2&RID=HGUFU3DD01R) | [Ribosomal RNA processing protein 1-like protein [*Triticum urartu*]](http://blast.ncbi.nlm.nih.gov/Blast.cgi#alnHdr_474311274) | 98% | 1e-163 |
| 45 | 137823004 | 137824495 | [EMT21602](http://www.ncbi.nlm.nih.gov/protein/475590936?report=genbank&log$=prottop&blast_rank=1&RID=HGUW3U10015) | [Peroxidase 12 [*Aegilops tauschii*]](http://blast.ncbi.nlm.nih.gov/Blast.cgi#alnHdr_475590936) | 81% | 2e-120 |
| 46 | 137872389 | 137874247 | [EMT32985](http://www.ncbi.nlm.nih.gov/protein/475625487?report=genbank&log$=prottop&blast_rank=1&RID=HGV29ND1014) | [N/A](http://blast.ncbi.nlm.nih.gov/Blast.cgi#alnHdr_475625487) |  |  |
| 47 | 138004303 | 138011130 | [NP_001147064](http://www.ncbi.nlm.nih.gov/protein/226529882?report=genbank&log$=prottop&blast_rank=7&RID=HGVCG7T0014) | [heat shock protein binding protein [*Zea mays*]](http://blast.ncbi.nlm.nih.gov/Blast.cgi#alnHdr_226529882) | 79% | 0 |
| 48 | 138046105 | 138047133 | [EMT13358](http://www.ncbi.nlm.nih.gov/protein/475559508?report=genbank&log$=prottop&blast_rank=1&RID=HGW86BJK014) | [Squamosa promoter-binding-like protein 8 [*Aegilops tauschii*]](http://blast.ncbi.nlm.nih.gov/Blast.cgi#alnHdr_475559508) | 100% | 9e-77 |
| 49 | 138069336 | 138069545 | [EMS58119](http://www.ncbi.nlm.nih.gov/protein/474190678?report=genbank&log$=prottop&blast_rank=3&RID=HGWCY131014) | [Indole-3-glycerol phosphate lyase, chloroplastic [*Triticum urartu*]](http://blast.ncbi.nlm.nih.gov/Blast.cgi#alnHdr_474190678) | 86% | 3e-28 |
| 50 | 138085071 | 138085982 | [EMT22445](http://www.ncbi.nlm.nih.gov/protein/475593668?report=genbank&log$=prottop&blast_rank=1&RID=HGWGKSSV01R) | [Dehydration-responsive element-binding protein 2A [*Aegilops tauschii*]](http://blast.ncbi.nlm.nih.gov/Blast.cgi#alnHdr_475593668) | 98% | 9e-135 |
| 51 | 138151483 | 138157024 | [EMT31378](http://www.ncbi.nlm.nih.gov/protein/475621181?report=genbank&log$=prottop&blast_rank=1&RID=HGWMYUCN01R) | [Zinc finger CCCH domain-containing protein 30 [*Aegilops tauschii*]](http://blast.ncbi.nlm.nih.gov/Blast.cgi#alnHdr_475621181) | 99% | 0 |
| 52 | 138157668: | 138158787 | [CDX97861](http://www.ncbi.nlm.nih.gov/protein/674935611?report=genbank&log$=prottop&blast_rank=1&RID=HGWTFKV001R) | [N/A](http://blast.ncbi.nlm.nih.gov/Blast.cgi" \l "alnHdr_674935611" \o "Go to alignment for BnaC04g40780D [Brassica napus]) |  |  |
| 53 | 138159736 | 138162171 | [EMT05261](http://www.ncbi.nlm.nih.gov/protein/475510268?report=genbank&log$=prottop&blast_rank=1&RID=HGX0X63A01R) | [Solute carrier family 22 member 15-like protein [*Aegilops tauschii*]](http://blast.ncbi.nlm.nih.gov/Blast.cgi#alnHdr_475510268) | 99% | 0 |
| 54 | 138165221 | 138165405 | [CAH67649](http://www.ncbi.nlm.nih.gov/protein/90265178?report=genbank&log$=prottop&blast_rank=23&RID=HGX75YU201R) | N/A |  |  |
| 55 | 138169606 | 138169893 | [CAE03004](http://www.ncbi.nlm.nih.gov/protein/38344803?report=genbank&log$=prottop&blast_rank=27&RID=HGXDMFCN01R) | N/A |  |  |
| 56 | 138225176 | 138227489 | [EMT01687](http://www.ncbi.nlm.nih.gov/protein/475450916?report=genbank&log$=prottop&blast_rank=1&RID=HGY0RC0901R) | [Tropinone reductase-like protein [*Aegilops tauschii*]](http://blast.ncbi.nlm.nih.gov/Blast.cgi#alnHdr_475450916) | 91% | 1e-50 |
| 57 | 138348362 | 138351513 | [EMT28400](http://www.ncbi.nlm.nih.gov/protein/475612417?report=genbank&log$=prottop&blast_rank=3&RID=HGY772R401R) | [Cell division protein ftsZ-like protein, chloroplastic [*Aegilops tauschii*]](http://blast.ncbi.nlm.nih.gov/Blast.cgi#alnHdr_475612417) | 99% | 0 |
| 58 | 138379976 | 138381933 | [EMS59176](http://www.ncbi.nlm.nih.gov/protein/474219391?report=genbank&log$=prottop&blast_rank=9&RID=HGYDSTMY01R) | [DEAD-box ATP-dependent RNA helicase 48 [*Triticum urartu*]](http://blast.ncbi.nlm.nih.gov/Blast.cgi#alnHdr_474219391) | 65% | 8e-34 |
| 59 | 138467493 | 138467999 | [AAM13439](http://www.ncbi.nlm.nih.gov/protein/20152973?report=genbank&log$=prottop&blast_rank=1&RID=HGYMR6WT01R) | [similar to putative receptor protein kinase from A. thaliana [*Hordeum vulgare subsp. vulgare*]](http://blast.ncbi.nlm.nih.gov/Blast.cgi#alnHdr_20152973) | 100% | 5e-43 |
| 60 | 138618534 | 138619371 | [NP_001136882](http://www.ncbi.nlm.nih.gov/protein/219363133?report=genbank&log$=prottop&blast_rank=5&RID=HGYXKS9J01R) | [putative TLD family protein [*Zea mays*]](http://blast.ncbi.nlm.nih.gov/Blast.cgi#alnHdr_219363133) | 83% | 3e-45 |
| 61 | 138644215 | 138645100 |  | N/A |  |  |
| 62 | 138658877 | 138663380 | [ABD37622](http://www.ncbi.nlm.nih.gov/protein/87312440?report=genbank&log$=prottop&blast_rank=1&RID=HH0WWZJH01R) | [serine-threonine protein kinase [*Triticum aestivum*]](http://blast.ncbi.nlm.nih.gov/Blast.cgi#alnHdr_87312440) | 99% | 0 |
| 63 | 138672248 | 138673503 | [EMT02313](http://www.ncbi.nlm.nih.gov/protein/475469510?report=genbank&log$=prottop&blast_rank=1&RID=HH15061F01R) | [Farnesyl pyrophosphate synthetase [*Aegilops tauschii*]](http://blast.ncbi.nlm.nih.gov/Blast.cgi#alnHdr_475469510) | 100% | 6e-77 |
| 64 | 138693118 | 138696007 | [EMT06016](http://www.ncbi.nlm.nih.gov/protein/475516680?report=genbank&log$=prottop&blast_rank=1&RID=HH202CN601R) | [Zinc finger CCCH domain-containing protein 31 [*Aegilops tauschii*]](http://blast.ncbi.nlm.nih.gov/Blast.cgi#alnHdr_475516680) | 100% | 0 |
| 65 | 138698132 | 138699056 | [EMT02313](http://www.ncbi.nlm.nih.gov/protein/475469510?report=genbank&log$=prottop&blast_rank=8&RID=HH29XWVT014) | [Farnesyl pyrophosphate synthetase [*Aegilops tauschii*]](http://blast.ncbi.nlm.nih.gov/Blast.cgi#alnHdr_475469510) | 100% | 2e-84 |
| 66 | 138719180 | 138721624 | [DAA60495](http://www.ncbi.nlm.nih.gov/protein/414884481?report=genbank&log$=prottop&blast_rank=22&RID=HH2GJCRP015) | [TPA: putative CRAL/TRIO domain containing, Sec14p-like phosphatidylinositol transfer family protein [*Zea mays*]](http://blast.ncbi.nlm.nih.gov/Blast.cgi#alnHdr_414884481) | 86% | 2e-29 |
| 67 | 138795143 | 138796013 | [EMT14831](http://www.ncbi.nlm.nih.gov/protein/475565588?report=genbank&log$=prottop&blast_rank=1&RID=HH2S1PW601R) | [Putative serine/threonine-protein kinase receptor [*Aegilops tauschii*]](http://blast.ncbi.nlm.nih.gov/Blast.cgi#alnHdr_475565588) | 99% | 6e-82 |
| 68 | 138806663 | 138807989 | [XP_007041489](http://www.ncbi.nlm.nih.gov/protein/590682988?report=genbank&log$=prottop&blast_rank=8&RID=HH31N5D401R) | [Oxidoreductase, putative [*Theobroma cacao*]](http://blast.ncbi.nlm.nih.gov/Blast.cgi#alnHdr_590682988) | 66% | 5e-93 |
| 69 | 138877064 | 138879966 | [EMT26270](http://www.ncbi.nlm.nih.gov/protein/475606048?report=genbank&log$=prottop&blast_rank=1&RID=HH3GH2T301R) | [Plasma membrane ATPase [*Aegilops tauschii*]](http://blast.ncbi.nlm.nih.gov/Blast.cgi#alnHdr_475606048) | 100% | 0 |
| 70 | 138932286 | 138938149 | EMT20109 | [N/A](http://blast.ncbi.nlm.nih.gov/Blast.cgi#alnHdr_475585624) |  |  |
| 71 | 138940723 | 138947002 | [EMT31390](http://www.ncbi.nlm.nih.gov/protein/475621193?report=genbank&log$=prottop&blast_rank=1&RID=HH41AFBK01R) | [Auxin response factor 11 [*Aegilops tauschii*]](http://blast.ncbi.nlm.nih.gov/Blast.cgi#alnHdr_475621193) | 96% | 0 |
| 72 | 139064780 | 139069156 | EMS50969 | 2-methoxy-6-polyprenyl-1,4-benzoquinol methylase, mitochondrial [*Triticum urartu*] | 99% | 1e-167 |
| 73 | 139067252 | 139071406 | BAF20825 | N/A | 89% | 2e-60 |
| 74 | 139131052 | 139131663 | [EMT13733](http://www.ncbi.nlm.nih.gov/protein/475561219?report=genbank&log$=prottop&blast_rank=1&RID=HKDGK6CA01R) | N/A | 98% | 2e-110 |
| 75 | 139145087 | 139151128 | EMT18108 | Disease resistance protein RPM1 [*Aegilops tauschii*] | 71% | 6e-11 |
| 76 | 139153802 | 139154200 | [EMT09554](http://www.ncbi.nlm.nih.gov/protein/475540161?report=genbank&log$=prottop&blast_rank=1&RID=HGW18WED014) | [Putative ribonuclease H protein [*Aegilops tauschii*]](http://blast.ncbi.nlm.nih.gov/Blast.cgi#alnHdr_475540161) | 74% | 3e-60 |
| 77 | 139192657 | 139192977 | EMS60315 | N/A |  |  |
| 78 | 139193673 | 139194048 | EMS60316 | N/A |  |  |
| 79 | 139200235 | 139206671 | [CAH65993](http://www.ncbi.nlm.nih.gov/protein/116308856?report=genbank&log$=prottop&blast_rank=5&RID=HGYEDRKE01R) | N/A |  |  |
| 80 | 139206824 | 139207787 | XP_014750930 | transcription factor IBH1 [*Brachypodium distachyon*] | 97% | 7e-15 |
| 81 | 139244852 | 139245784 | EMT28401 | N/A |  |  |
| 82 | 139342698 | 139344779 | ACT22500 | plastid glutamine synthetase 2 [*Triticum aestivum*] | 98% | 9e-66 |
| 83 | 139348585 | 139351702 | EMS55336 | DNA topoisomerase 1 [*Triticum urartu*] | 80% | 0 |
| 84 | 139364799 | 139366357 | [EMS51822](http://www.ncbi.nlm.nih.gov/protein/473989908?report=genbank&log$=prottop&blast_rank=8&RID=HGXVW3Y701R) | [Transmembrane 9 superfamily member 4 [*Triticum urartu*]](http://blast.ncbi.nlm.nih.gov/Blast.cgi#alnHdr_473989908) | 97% | 0 |
| 85 | 139369519 | 139370195 | EMT08392 | Tropinone reductase-like protein [*Aegilops tauschii*] | 88% | 4e-53 |
| 86 | 139380317 | 139381181 | EMT03631 | N/A |  |  |
| 87 | 139385118 | 139386329 | [EMS51820](http://www.ncbi.nlm.nih.gov/protein/473989906?report=genbank&log$=prottop&blast_rank=13&RID=HGXF9NZH01R) | N/A |  |  |
| 88 | 139393899 | 139394513 | EMT03801 | Transmembrane 9 superfamily member 2 [*Aegilops tauschii*] | 98% | 1e-93 |
| 89 | 139406363 | 139408447 | EMT09611 | U3 small nucleolar ribonucleoprotein IMP3 [*Aegilops tauschii*] | 99% | 2e-130 |
| 90 | 139416283 | 139418780 | [NP_001150358](http://www.ncbi.nlm.nih.gov/protein/226509390?report=genbank&log$=prottop&blast_rank=6&RID=HKF2DSKD01R) | N/A |  |  |
| 91 | 139418870 | 139423384 | [BAB78487](http://www.ncbi.nlm.nih.gov/protein/17297979?report=genbank&log$=prottop&blast_rank=10&RID=HGX40F4K01R) | [26S proteasome regulatory particle non-ATPase subunit8 [*Oryza sativa Japonica Group*]](http://blast.ncbi.nlm.nih.gov/Blast.cgi#alnHdr_17297979) | 96% | 0 |
| 92 | 139423383 | 139425862 | NP_001148631 | [cofactor required for Sp1 transcriptional activation subunit 9 [*Zea mays*]](http://blast.ncbi.nlm.nih.gov/Blast.cgi#alnHdr_226504170) | 84% | 2e-90 |
| 93 | 139426313 | 139429032 | [EAY80119](http://www.ncbi.nlm.nih.gov/protein/125533571?report=genbank&log$=prottop&blast_rank=3&RID=HGNZK24801R) | N/A | 70% | 0 |
| 94 | 139433507 | 139438076 | EMT27206 | N/A | 99% | 0 |
| 95 | 139451254 | 139452007 | [EMT02314](http://www.ncbi.nlm.nih.gov/protein/475469511?report=genbank&log$=protalign&blast_rank=1&RID=HESB9NY3016) | N/A | 100% | 0 |
| 96 | 139459736 | 139460629 | [EMT22169](http://www.ncbi.nlm.nih.gov/protein/475592708?report=genbank&log$=protalign&blast_rank=1&RID=HESJU085016) | N/A | 100% | 2e-104 |
| 97 | 139462450 | 139467993 | EMT22168 | Inositol-pentakisphosphate 2-kinase [*Aegilops tauschii*] | 99% | 0 |
| 98 | 139468506 | 139473220 | NP_001149849 | ATP/GTP binding protein [*Zea mays*] | 86% | 7e-171 |
| 99 | 139481285 | 139483959 | EMS49112 | Kinesin-like calmodulin-binding protein-like protein [*Triticum urartu*] | 96% | 4e-172 |
| 100 | 139529527 | 139529688 | EMT19210 | N/A | 94% | 2e-26 |
| 101 | 139540992 | 139543460 | EMT00056\| | Cysteine-rich receptor-like protein kinase 10 [*Aegilops tauschii*] | 85% | 0 |
| 102 | 139603555 | 139609219 | EMS61907 | Ribulose bisphosphate carboxylase/oxygenase activase, chloroplastic [*Triticum urartu*] | 906% |  |
| 103 | 139613120 | 139617105 | EMT03164 | Molybdopterin biosynthesis protein CNX1 [*Aegilops tauschii*] | 99% | 0 |
| 104 | 139621388 | 139623345 | EMT16430 | Putative serine/threonine-protein kinase receptor [*Aegilops tauschii*] | 86% | 0 |
| 105 | 139623450 | 139623847 | EMT14107 | N/A | 99% | 2e-35 |
| 106 | 139629414 | 139630020 | [[EMT12050](http://www.ncbi.nlm.nih.gov/protein/475582694?report=genbank&log$=protalign&blast_rank=1&RID=HETXP84K016)](http://www.ncbi.nlm.nih.gov/protein/474311276?report=genbank&log$=protalign&blast_rank=1&RID=HEV730YC01N) | N/A | 84% | 3e-45 |
| 107 | 139641504 | 139648468 | EMT32641 | Protein RCC2 [*Aegilops tauschii*] | 94% | 0 |
| 108 | 139666505 | 139673836 | EMT11405 | Palmitoyltransferase akr1 [*Aegilops tauschii*] | 74% | 0 |
| 109 | 139700814 | 139705221 | CAH67057 | N/A |  |  |
| 110 | 139725509 | 139730656 | EMT16310 | Putative serine/threonine-protein kinase [*Aegilops tauschii*] | 81% | 1e-100 |
| 111 | 139765002 | 139766618 | [CDJ26576](http://www.ncbi.nlm.nih.gov/protein/475582694?report=genbank&log$=protalign&blast_rank=1&RID=HETXP84K016) | N/A | 87% | 8e-25 |
| 112 | 139767125 | 139772663 | [CDJ26577](http://www.ncbi.nlm.nih.gov/protein/475582694?report=genbank&log$=protalign&blast_rank=1&RID=HETXP84K016) | N/A | 95% | 0 |
| 113 | 139848432 | 139849569 | AAL73394 | glutathione transferase [*Hordeum vulgare subsp. vulgare*] | 82% | 3e-88 |
| 114 | 139923246 | 139927017 | EMT25260 | Glutamyl-tRNA(Gln) amidotransferase subunit A [*Aegilops tauschii*] | 99% | 0 |
| 115 | 139955802 | 139957104 | BAS77745 | N/A | 74% | 6e-55 |
| 116 | 139996503 | 140005206 | DAA35664 | TPA: hypothetical protein ZEAMMB73_337226 [*Zea mays*] | 87% | 0 |
| 117 | 140005754 | 140007594 | EMS67906 | D-3-phosphoglycerate dehydrogenase, chloroplastic [*Triticum urartu*] | 99% | 0 |
| 118 | 140048086 | 140052118 | CAJ86155 | N/A | 85% | 0 |
| 119 | 140072326 | 140077817 | EMT13303 | N/A | 66% | 0 |
| 120 | 140159413 | 140160293 | EMS65931 | Lipoxygenase 1 [*Triticum urartu*] | 63% | 8e-42 |
| 121 | 140170664 | 140171994 | EMS52484 | Xyloglucan endotransglucosylase/hydrolase protein 9 [*Triticum urartu*] | 97% | 2e-147 |
| 122 | 140172601 | 140175090 | EMT1127 | JmjC domain-containing protein [*Aegilops tauschii*] | 94% | 0 |
| 123 | 140185224 | 140186833 | ACT32453 | C2 domain-containing protein [*Triticum aestivum*] | 98% | 1e-115 |
| 124 | 140187071 | 140188039 | EMT32980 | Cytochrome P450 84A1 [*Aegilops tauschii*] | 97% | 5e-151 |
| 125 | 140207374 | 140208602 | EMT30728 | Ubiquitin carboxyl-terminal hydrolase 22 [*Aegilops tauschii*] | 98% | 5e-127 |
| 126 | 140239129 | 140240185 | EMT24426 | Putative lipoate-protein ligase A [*Aegilops tauschii*] | 100% | 2e-52 |
| 127 | 140254246 | 140256709 | EMT14537 | Universal stress protein A-like protein [*Aegilops tauschii*] | 100% | 6e-115 |


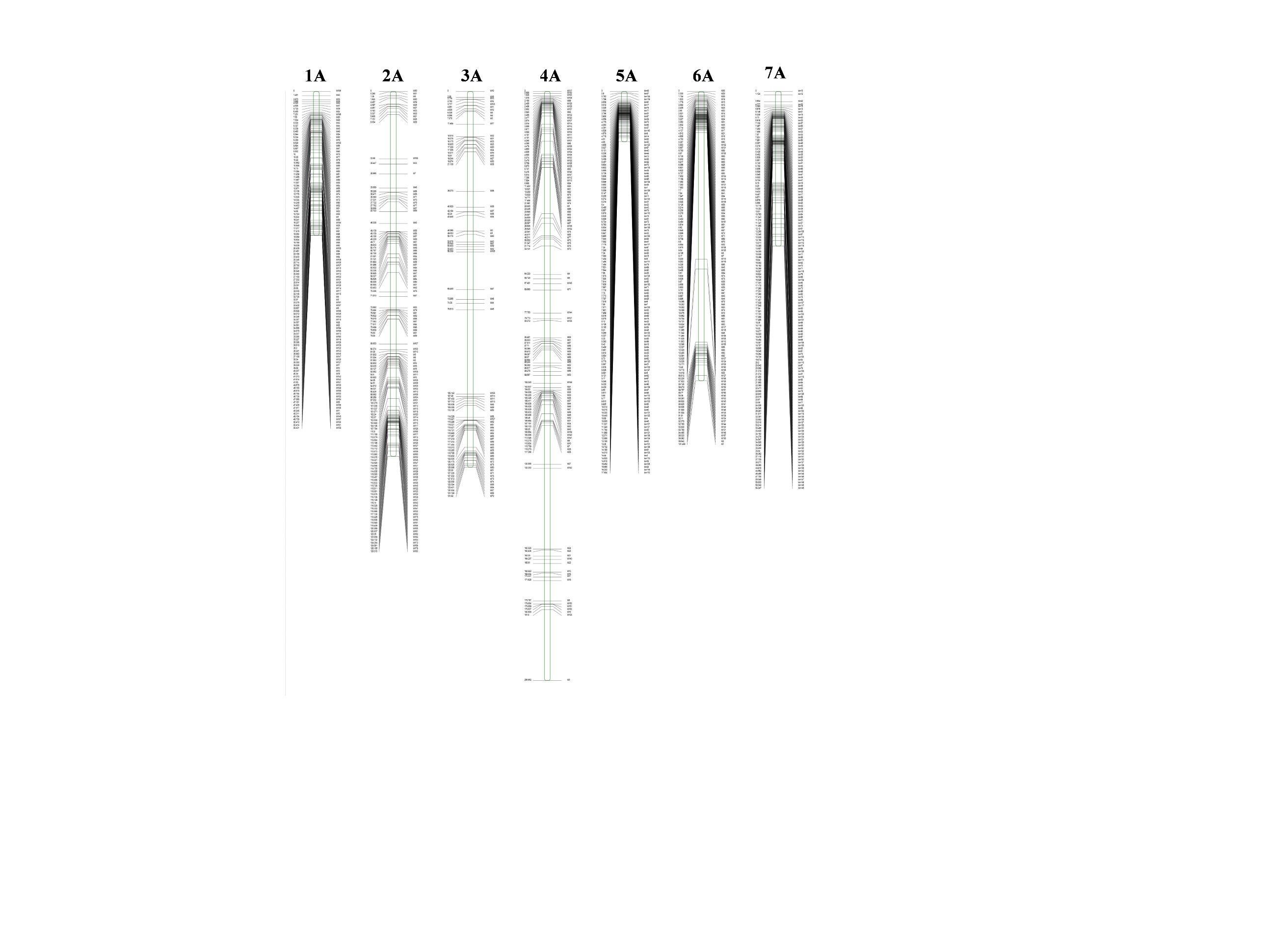


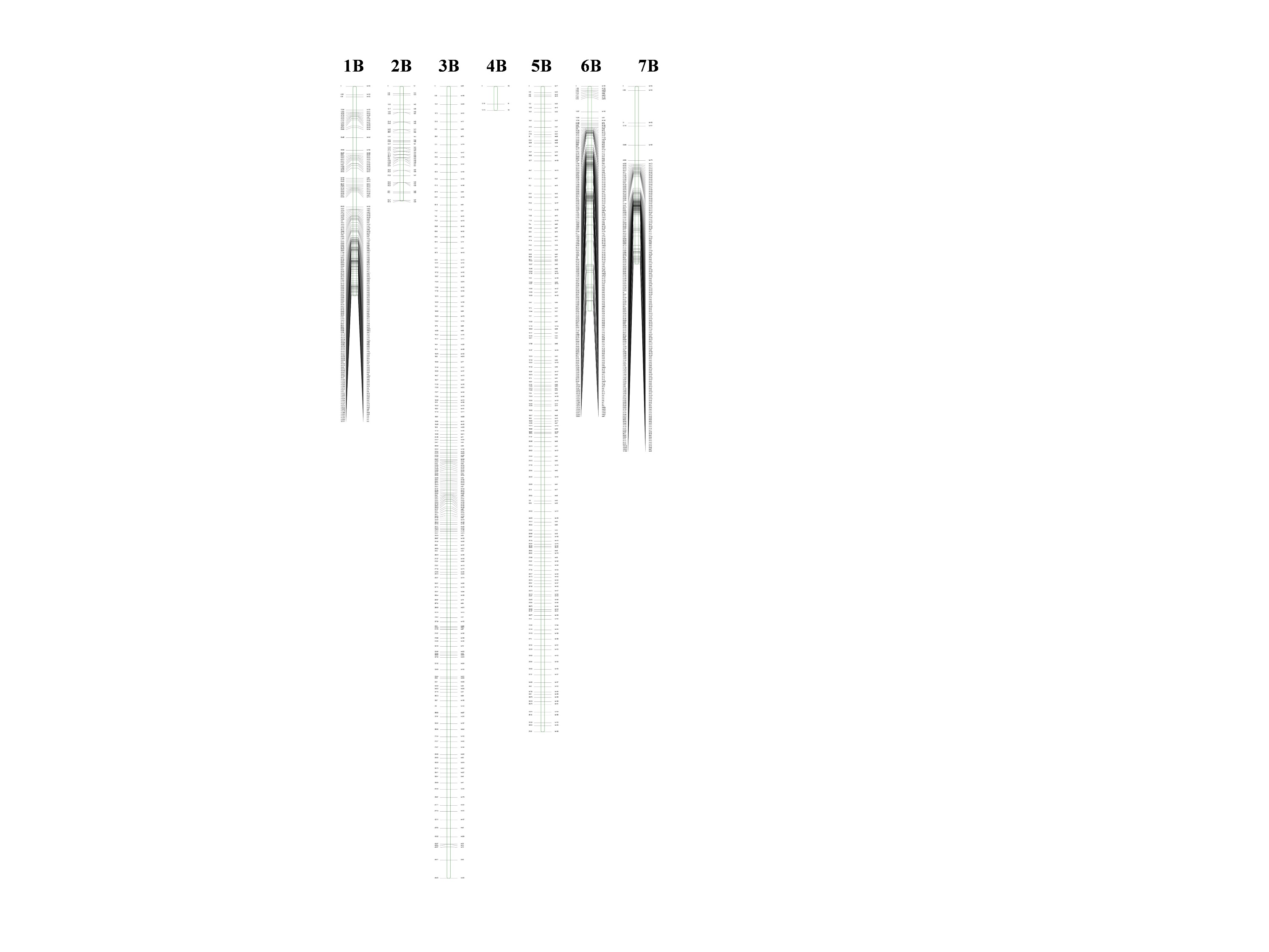


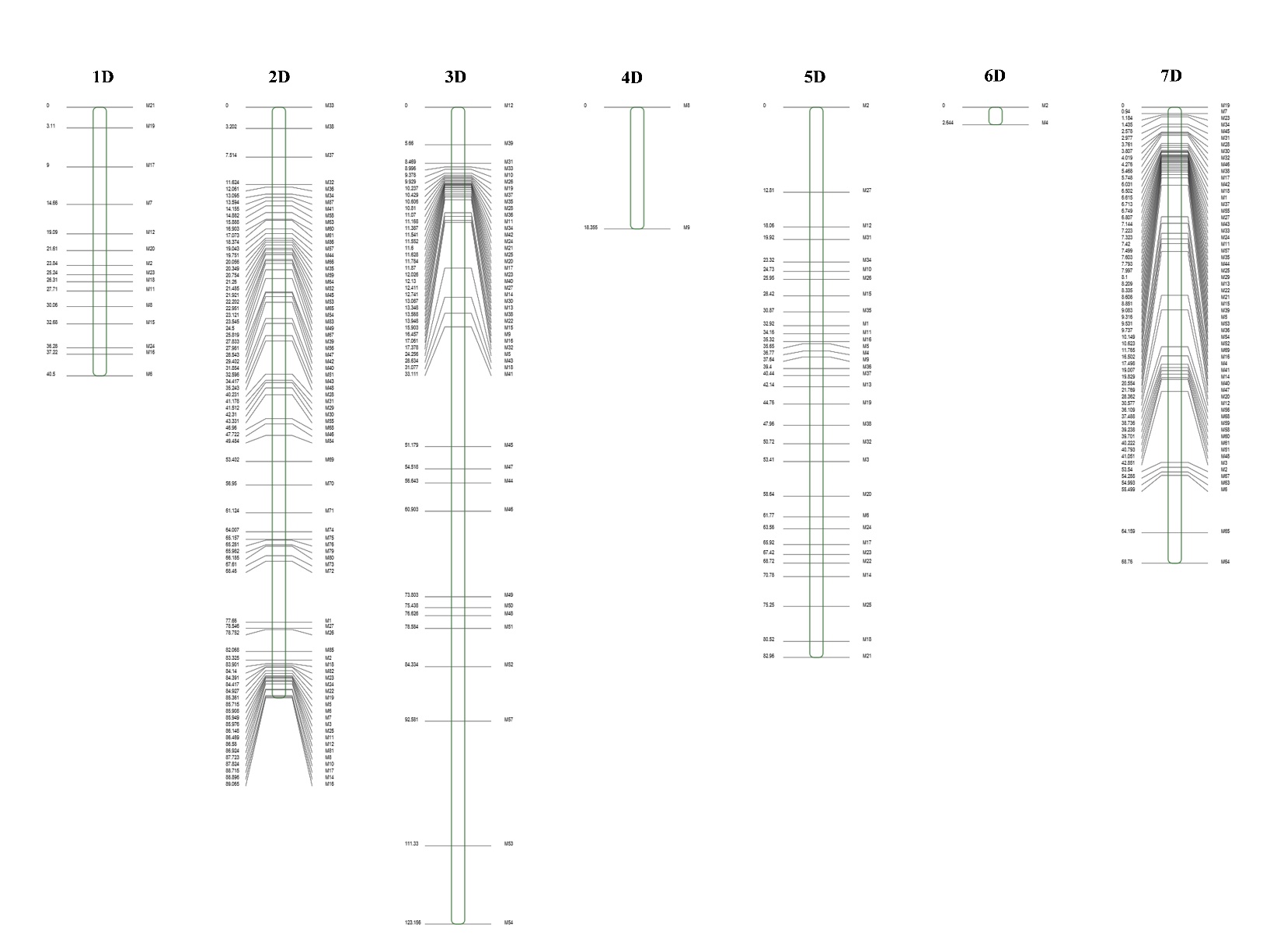
Figure S1 High-density genetic map marker information
